# Seed-coat protective neolignans are produced by the dirigent protein AtDP1 and the laccase AtLAC5 in Arabidopsis

**DOI:** 10.1093/plcell/koaa014

**Published:** 2020-11-27

**Authors:** Keiko Yonekura-Sakakibara, Masaomi Yamamura, Fumio Matsuda, Eiichiro Ono, Ryo Nakabayashi, Satoko Sugawara, Tetsuya Mori, Yuki Tobimatsu, Toshiaki Umezawa, Kazuki Saito

**Affiliations:** 1 RIKEN Center for Sustainable Resource Science, 1-7-22, Suehiro-cho, Tsurumi-ku, Yokohama 230-0045, Japan; 2 Research Institute for Sustainable Humanosphere, Kyoto University, Gokasho, Uji, Kyoto 611-0011, Japan; 3 Research Institute, Suntory Global Innovation Center Ltd., 8-1-1 Seikadai, Seika, Soraku-gun, Kyoto 619-0284, Japan; 4 Research Unit for Development of Global Sustainability, Kyoto University, Gokasho, Uji, Kyoto 611-0011, Japan; 5 Plant Molecular Science Center, Chiba University, 1-8-1, Inohana, Chuo-ku, Chiba 260-8675, Japan

## Abstract

Lignans/neolignans are generally synthesized from coniferyl alcohol (CA) in the cinnamate/monolignol pathway by oxidation to generate the corresponding radicals with subsequent stereoselective dimerization aided by dirigent proteins (DIRs). Genes encoding oxidases and DIRs for neolignan biosynthesis have not been identified previously. In *Arabidopsis thaliana*, the DIR AtDP1/AtDIR12 plays an essential role in the 8-*O*-4′ coupling in neolignan biosynthesis by unequivocal structural determination of the compound missing in the *atdp1* mutant as a sinapoylcholine (SC)-conjugated neolignan, *erythro*-3-{4-[2-hydroxy-2-(4-hydroxy-3-methoxyphenyl)-1-hydroxymethylethoxy]-3,5-dimethoxyphenyl}acryloylcholine. Phylogenetic analyses showed that AtDP1/AtDIR12 belongs to the DIR-a subfamily composed of DIRs for 8-8′ coupling of monolignol radicals. *AtDP1/AtDIR12* is specifically expressed in outer integument 1 cells in developing seeds. As a putative oxidase for neolignan biosynthesis, we focused on *AtLAC5*, a laccase gene coexpressed with *AtDP1/AtDIR12*. In *lac5* mutants, the abundance of feruloylcholine (FC)-conjugated neolignans decreased to a level comparable to those in the *atdp1* mutant. In addition, SC/FC-conjugated neolignans were missing in the seeds of mutants defective in SCT/SCPL19, an enzyme that synthesizes SC. These results strongly suggest that AtDP1/AtDIR12 and AtLAC5 are involved in neolignan biosynthesis via SC/FC. A tetrazolium penetration assay showed that seed coat permeability increased in *atdp1* mutants, suggesting a protective role of neolignans in *A*. *thaliana* seeds.

## Teaser

The dirigent protein AtDP1/AtDIR12 and the laccase AtLAC5 play essential roles in the biosynthesis of seed-coat protective neolignans via sinapoylcholine/feruloylcholine in Arabidopsis.

## Introduction

Phenolic compounds including lignins, lignans, neolignans, and flavonoids are major plant secondary metabolites (also known as specialized metabolites; Petersen et al., 2010; Ralph et al., 2019). These metabolites are derived from the phenylpropanoid metabolic pathway and are widely distributed in the plant kingdom (Weng and Chapple, 2010). Phenolic compounds play an important role in the structural enhancement of the plant body, biological defense, environmental stress tolerance, and interactions between plants and other organisms, and also contribute to human health as pharmaceuticals, dietary supplements, flavors, and pigments (Vogt, 2010).

**Figure:**
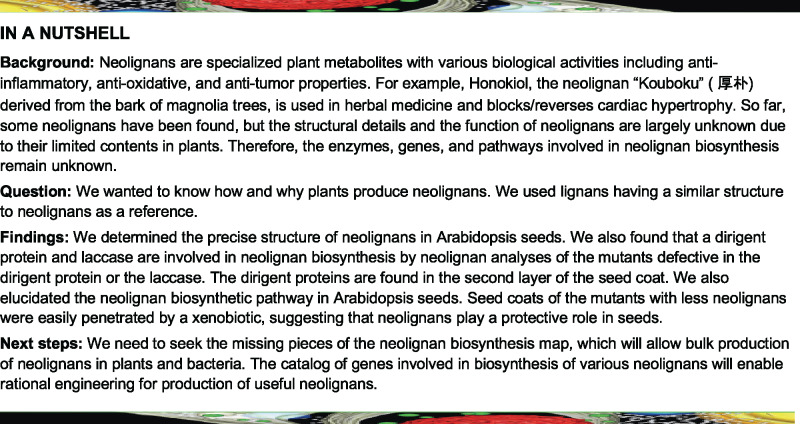


**Figure 1:**
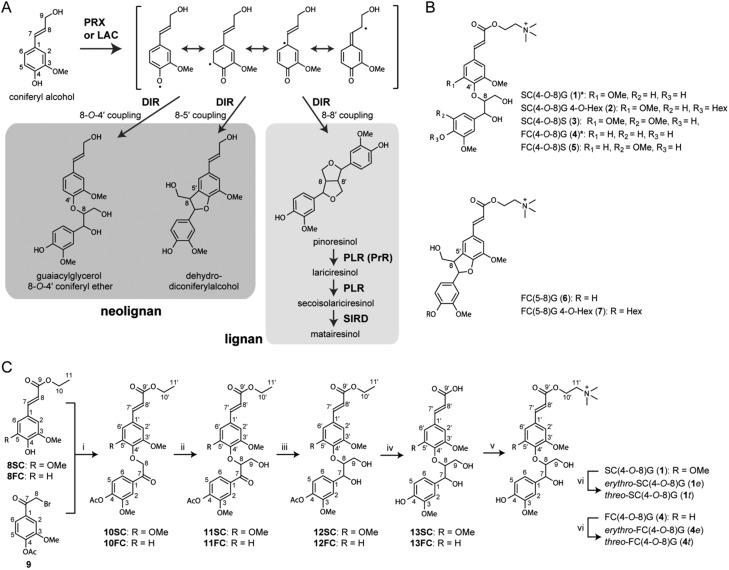
Lignans and neolignans. (A) The biosynthetic pathways of lignans and neolignans. DIR, dirigent protein; LAC, laccase; PLR, pinoresinol/lariciresinol reductase; PrR, pinoresinol reductase; PRX, peroxidase; SIRD, secoisolariciresinol dehydrogenase. (B) Structures of neolignans in Arabidopsis seeds (see also Supplemental Table 1). G, Guaiacyl moiety; S, Syringyl moiety; SC, sinapoylcholine; FC, feruloylcholine; Hex, hexose. Asterisks indicate the compounds authenticated with chemically synthesized standards. (C) Synthetic scheme for neolignans SC(4-*O*-8)G (**1**) and FC(4-*O*-8)G (**4**). Reaction conditions: (i) acetone/K_2_CO_3_/reflux; (ii) dioxane/K_2_CO_3_/formaldehyde/r.t.; (iii) ethanol/NaBH_4_/r.t.; (iv) NaOH aq./r.t.; (v) DMF/bromocholine bromide/NaOH/90°C; (vi) preparative HPLC. r.t., room temperature.

Lignans are a class of dimeric phenylpropanoid metabolites [(C_6_C_3_)_2_] with a C8-C8′ linkage; compounds with all other types of linkages are called neolignans (Moss, 2000; Umezawa, 2003a, 2003b). The biosynthesis of lignans such as pinoresinol, matairesinol, lariciresinol, and secoisolariciresinol is initiated with coniferyl alcohol (CA) in the cinnamate/monolignol pathway and has been well-studied. CA is oxidized by oxidases such as peroxidases and laccases (LACs) to give rise to the corresponding radical, which is then dimerized stereoselectively at the C8 and C8′ positions to a lignan, pinoresinol, with the aid of dirigent proteins (DIRs; Davin and Lewis, 2003; Umezawa, 2003a; Suzuki and Umezawa, 2007; Figure 1A). Pinoresinol is further converted to matairesinol via lariciresinol and secoisolariciresinol by pinoresinol/lariciresinol reductase and secoisolariciresinol dehydrogenase (Davin and Lewis, 2003; Umezawa, 2003a; Suzuki and Umezawa, 2007; Figure 1A). In Arabidopsis (*Arabidopsis thaliana*), pinoresinol is converted to lariciresinol by pinoresinol reductase (Nakatsubo et al., 2008). The biosynthetic pathway from CA to matairesinol is common to a number of plant species, and a wide variety of lignans are derived from pinoresinol, lariciresinol, secoisolariciresinol, and matairesinol by aromatic substituent modification (Umezawa, 2003a).

In contrast, the biosynthetic pathway for neolignans remains largely unknown. Neolignans are also derived from monolignols generated in the cinnamate/monolignol pathway (Umezawa et al., 2011). The biosynthetic pathways for producing lignan and neolignans diverge at the step for oxidative coupling of monolignols (Figure 1A). Several studies dealing with crude enzyme preparations mediating the formation of neolignans from phenylpropanoid monomers have been reported (Orr and Lynn, 1992; Katayama and Kado, 1998; Sartorelli et al., 2001; Lourith et al., 2005; Umezawa et al., 2011); however, there are no published reports of genes encoding oxidases and DIRs involved in neolignan biosynthesis.

DIR was originally identified as a protein for guiding the stereoselective coupling of CA radicals in the production of (+)-pinoresinol, a lignan, in *Forsythia* sp. (Davin et al., 1997). Subsequently, genes encoding (+)- or (−)-pinoresinol-forming DIRs were found in a variety of seed plants including Arabidopsis (Davin et al., 1997; Kim et al., 2002, 2012, 2015; Ralph et al., 2007; Pickel et al., 2010; Dalisay et al., 2015; Seneviratne et al., 2015; Gasper et al., 2016). DIR-mediated enantioselective bimolecular coupling is not restricted to the CA radical. Atroposelective formation of (+)-gossypol from hemigossypol, a sesquiterpene phenol, is also mediated by DIRs in *Gossypium* spp. (Effenberger et al., 2015, 2017). Moreover, pterocarpan synthases with dirigent-like domains have also been reported to occur in several plants (Uchida et al., 2017; Meng et al., 2020).

DIRs belong to a multi-gene family that is classified into six subfamilies (DIR-a, DIR-b/d, DIR-c, DIR-e, DIR-f, and DIR-g) based on phylogenetic analyses (Ralph et al., 2007). The DIRs involved in lignan biosynthesis (guiding 8-8′ coupling) belong to the DIR-a subfamily and are functionally conserved among plant species.

In Arabidopsis, lignans and neolignans are localized in leaves and roots (Böttcher et al., 2008; Nakatsubo et al., 2008; Okazawa et al., 2011; Dima et al., 2015) and neolignans are also present in seeds (Böttcher et al., 2008). Arabidopsis contains 25 DIR genes (*AtDIR1*–*AtDIR25*) that are distributed in the DIR-a, DIR-b/d, and DIR-e subfamilies in addition to an atypical type, *OVULE ABORTION 2* (At5g49030.3; Paniagua et al., 2017). AtDIR6 in the DIR-a subfamily was identified as a (−)-pinoresinol-forming DIR based on overexpression experiments, RNAi approaches, and recombinant protein assays (Pickel et al., 2010; Kim et al., 2012). Recombinant AtDIR5 protein (DIR-a subfamily) also has a (−)-pinoresinol-forming activity *in vitro* (Kim et al., 2012). *ENHANCED SUBERIN 1* (*ESB1*, *AtDIR10*) in the DIR-e subfamily is essential for building the extracellular lignin-based Casparian strip (Hosmani et al., 2013; Barbosa et al., 2019); however, the physiological functions of the other Arabidopsis DIRs are still unknown.

Based on the correlation analyses of gene expression and metabolite accumulation, we found an orphan DIR gene, *AtDP1*/*AtDIR12* (At4g11180, DIR-a subfamily), that showed no significant correlation with putative lignans and neolignans that accumulate in roots and other DIR genes (Matsuda et al., 2010). The preliminary data suggested that the T-DNA inserted *atdp1* mutant lacked a putative neolignan compound with a peak at *m*/*z* 668 (Matsuda et al., 2010); however, the chemical structure of the compound missing in the mutant and AtDP1/AtDIR12 function remained to be verified.

Here, we report the identification of a sinapoylcholine (SC)-conjugated neolignan, *erythro*-3-{4-[2-hydroxy-2-(4-hydroxy-3-methoxyphenyl)-1-hydroxymethylethoxy]-3,5-dimethoxyphenyl}acryloylcholine, SC(4-*O*-8)G (**1***e*; Figure 1) as the compound missing in the *atdp1* mutant. The chemically synthesized compound was used for confirmation of this result. A complementation test of the *atdp1* mutant with *AtDP1/AtDIR12* genomic clones demonstrated that AtDP1/AtDIR12 is essential for the biosynthesis of 8-*O*-4′-type neolignans in seeds. Transgenic plants expressing *AtDP1/AtDIR12* promoter–reporter constructs show that AtDP1/AtDIR12 is localized in the outer integument 1 (oi1) cells of seeds. In addition, we found that mutants deficient in *AtLAC5*, a *LAC* co-expressed with *AtDP1/AtDIR12* showed significant metabolic changes among feruloylcholine (FC)-conjugated neolignans, including *erythro*-3-{4-[2-hydroxy-2-(4-hydroxy-3-methoxyphenyl)-1-hydroxymethylethoxy]-3-methoxyphenyl}acryloylcholine, FC(4-*O*-8)G (**4***e* in Figure 1). Neolignan analysis of the mutants defective in the SC biosynthesis ability showed that SC- and FC-conjugated neolignans are synthesized from SC/FC and CA. A tetrazolium salt assay showed that seed coat permeability was increased in the *atdp1* mutants. These data indicate that AtDP1/AtDIR12 and AtLAC5 are involved in the neolignan biosynthetic pathway and suggest that neolignans play an important role in protection from external factors.

## Results

### The *atdp1* mutants lack putative neolignans

**Figure 2:**
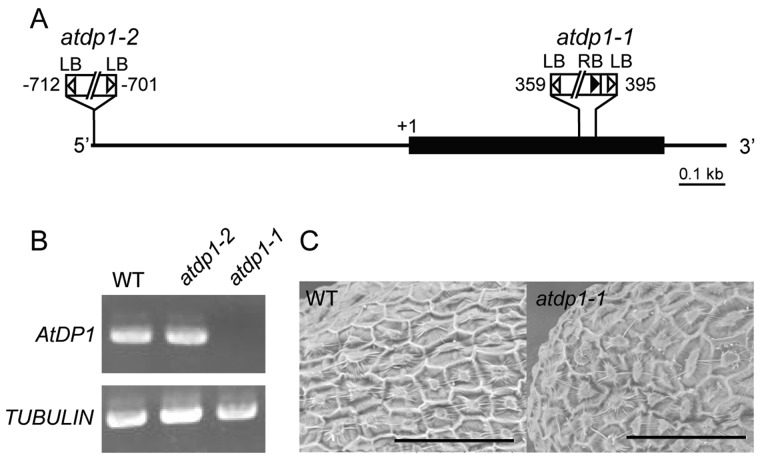
T-DNA insertion mutants of At*DP1/AtDIR12*. (A) Schematic representation of *AtDP1/AtDIR12* with two T-DNA insertion mutants used in this study. The thick line indicates the coding region and the thinner line indicates the 5′- and 3′-untranslated regions. *AtDP1/AtDIR12* has no introns. White and black triangles show the left and right borders, respectively. Numbers indicate the position of the T-DNA insertion. LB, left border; RB, right border. (B) RT-PCR analysis of transcripts in wild-type (WT, Col-0) and two independent homozygous mutant lines (*atdp1-1* and *atdp1-2*). C, Scanning electron micrograph of epidermal cells from a dry seed of WT (Col-0) and the homozygous *atdp1-1* mutant. Scale bars = 100 µm.

We previously reported that the T-DNA insertion mutant designated *dp1* (SAIL_60_D04) lacked a compound having a peak at *m*/*z* 668 in seeds (Matsuda et al., 2010). An additional homozygous T-DNA insertion mutant, SALK_062238, was isolated and designated as *atdp1-2*, and *dp1* (SAIL_60_D04) was renamed *atdp1-1* (Figure 2A). The T-DNA was inserted between −711 and −702 base pairs of the *AtDP1/AtDIR12* promoter region in *atdp1-2*. Reverse transcription-polymerase chain reaction (RT-PCR) analysis detected *AtDP1/AtDIR12* transcripts in *atdp1-2*, whereas the transcript was not detected in *atdp1-1* (Figure 2B), indicating that *atdp1-1* is a null allele of the *AtDP1/AtDIR12* gene. No obvious phenotypic differences were observed in the appearances of wild-type and *atdp1-1* mutant seeds by scanning electron microscopy (Figure 2C).

**Figure 3:**
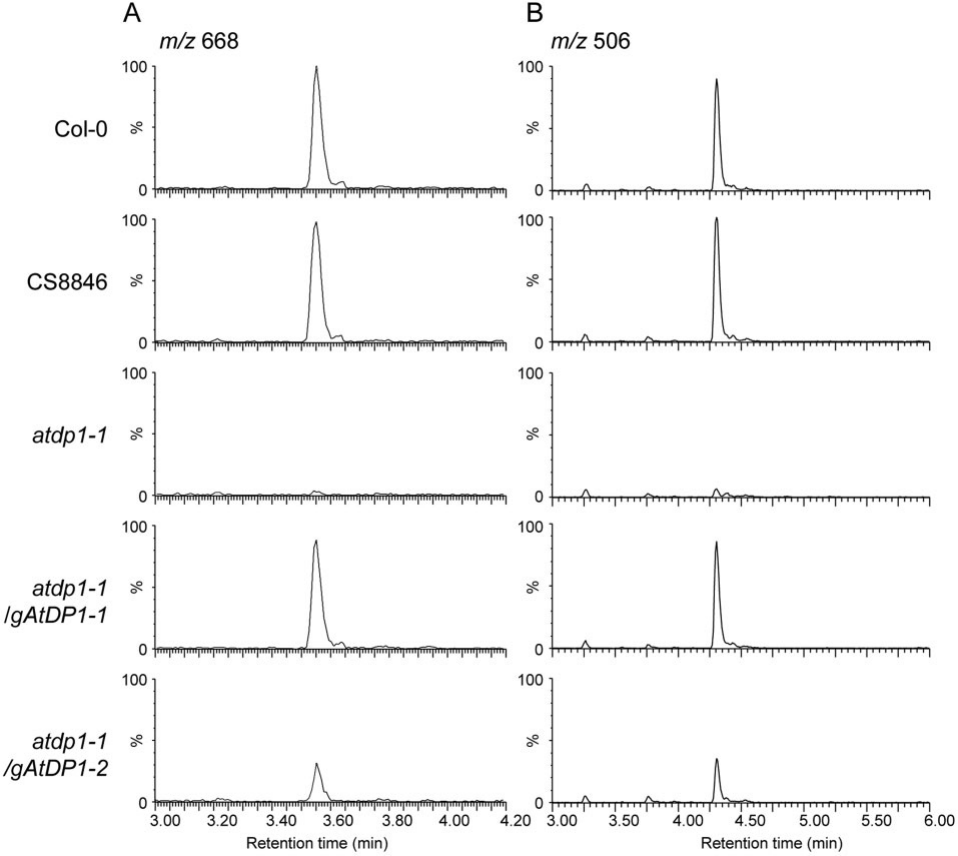
UPLC-MS analyses of the *atdp1* mutant lines. Extracted ion chromatograms at *m/z* 668 (A) and 506 (B) of wild-type (Col-0), a parental line of *atdp1-1* (CS8846), the *atdp1*-deficient mutant (*atdp1-1*), and the *atdp1*-deficient mutant complemented with 3.0-kb and 1.9-kb *DP1* genomic fragments (*atdp1-1*/*gAtDP1-1* and *atdp1-1*/*gAtDP1-2*, respectively).

Metabolome analyses showed that a compound with a peak at *m*/*z* 506 corresponding to a new putative neolignan compound and the previously reported putative neolignan compound (*m*/*z *=* *668; Matsuda et al., 2010) were both missing in seeds of the *atdp1-1* mutant (Figure 3). To assess whether the *AtDP1/AtDIR12* mutation was responsible for the absence of the two compounds observed in the mutant, we performed a complementation test of the *atdp1-1* mutant. The mutant was transformed with two types of *AtDP1/AtDIR12* genomic clones (*gAtDP1-1* and *gAtDP1-2*, 3.0 kb/1.9 kb *AtDP1/AtDIR12* genomic fragments containing 1.8-kb/0.72-kb promoter regions, respectively). Independent transgenic lines carrying *gAtDP1-1* and *gAtDP1-2* had essentially the same metabolic profile as wild-type plants, although the levels of the target compound were lower in transgenic lines carrying *gAtDP1-2* (Figure 3). These data demonstrated that *AtDP1/AtDIR12* is crucial for the biosynthesis of these two putative neolignan compounds in seeds, and that a 1.9-kb *AtDP1/AtDIR12* genomic fragment containing 0.72 kb of the promoter region contains at least the minimum region necessary for functional complementation of the metabolic defect in *atdp1-1*.

### Structural identification of a seed-specific neolignan missing in the *atdp1* mutant

The structure of the putative neolignan compound (*m*/*z *=* *668) missing in the *atdp1-1* mutant was proposed to be 3-{4-[2-hydroxy-2-(4-hexosyloxy-3-methoxyphenyl)-1-hydroxymethylethoxy]-3,5-dimethoxyphenyl}acryloylcholine, SC(4-*O*-8)G 4-*O*-Hex (**2**; Figure 1B), based on mass spectral (MS/MS) data of a chemical class of compounds and/or spectral similarity to known compounds of a chemical class (Matsuda et al., 2010). Similarly, the other missing compound (*m*/*z *=* *506) was proposed to be 3-{4-[2-hydroxy-2-(4-hydroxy-3-methoxyphenyl)-1-hydroxymethylethoxy]-3,5-dimethoxyphenyl}acryloylcholine, SC(4-*O*-8)G (**1**; Figure 1B; Matsuda et al., 2010).

**Figure 4:**
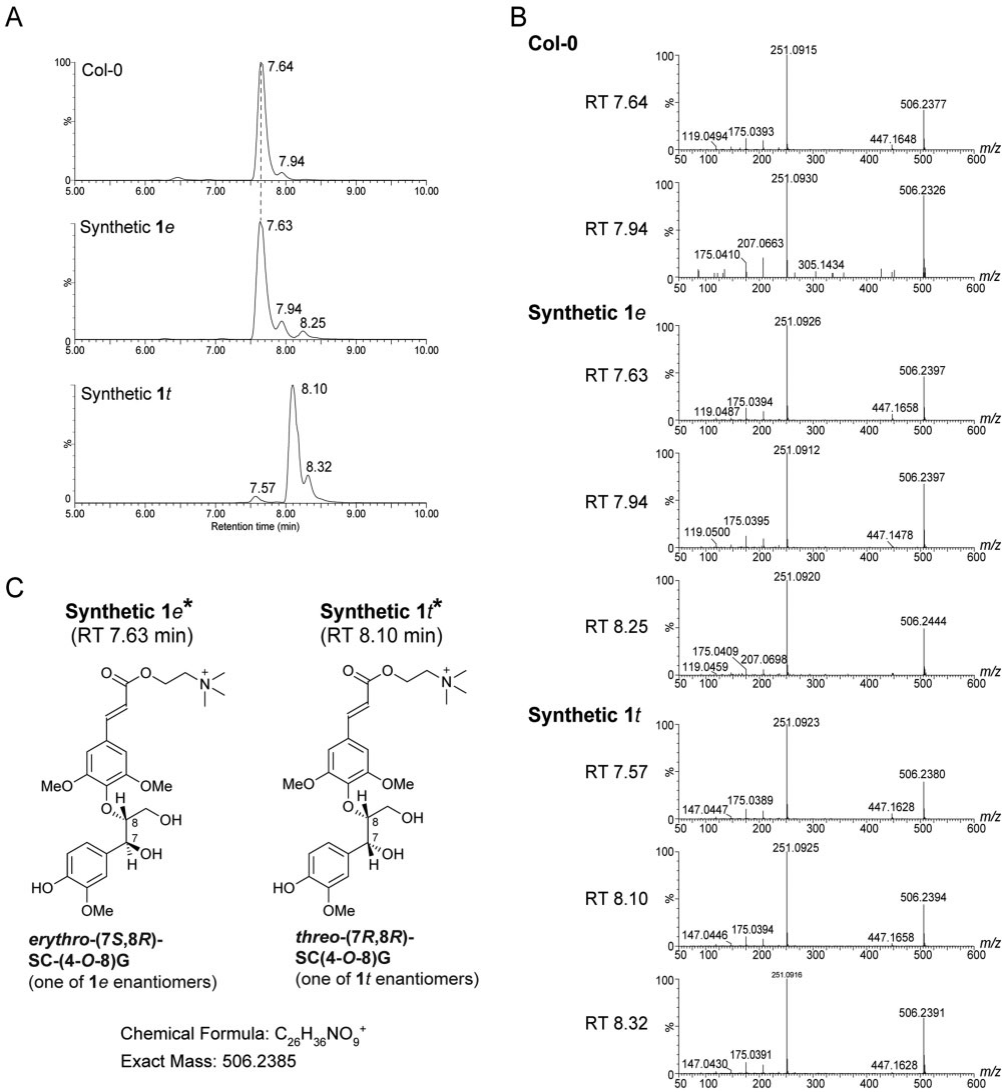
Identification of neolignan SC(4-*O*-8)G (**1**) in Arabidopsis seeds. UPLC chromatograms (A) and MS/MS spectra (B) of synthetic standards of *erythro*-SC(4-*O*-8)G (**1***e*) and *threo*-SC(4-*O*-8)G (**1***t*) and the corresponding product from Col-0 are shown. The structural interpretation of fragment ions was shown in Böttcher et al. (2008). (C) Structures of synthetic **1***e* and **1***t*. *Synthetic **1***e* (RT 7.63 min) and **1***t* (RT 8.10 min) are each racemic mixtures. Only the (7*S*, 8*R*) enantiomer of **1***e* and the (7*R*, 8*R*) enantiomer of **1***t* are depicted. RT, retention time.

To determine the structures of the putatively annotated neolignan compounds missing in the *atdp1* mutant, we chemically synthesized authentic standards (racemates) for the two stereoisomers of neolignan **1**, namely *erythro*-SC(4-*O*-8)G (**1***e*) and *threo*-SC(4-*O*-8)G (**1***t*), as shown in Figure 1C and further described in Materials and methods section. The structural identities of the synthesized compounds were validated by nuclear magnetic resonance (NMR) spectroscopy with 1D ^1^H and ^13^C and 2D correlation spectroscopy (COSY), heteronuclear single quantum correlation (HSQC), and heteronuclear multiple bond coherence (HMBC) pulse schemes (see Materials and methods section and Supplemental Data Set 1). The stereochemistry of **1***e* and **1***t* were based on close comparisons of their NMR chemical shift and coupling constant data with those reported in the literature (Ralph and Helm, 1991; Helm and Ralph, 1992; Wolfram et al., 2010). The retention time on ultra performance liquid chromatography (UPLC; 7.64 min), mass and MS/MS spectra of the missing compound (*m*/*z *=* *506) in the *atdp1-1* mutant were identical to those of the authentic standard **1***e* (Figure 4). In addition, we confirmed that MS/MS spectra of the products (Figure 4) were identical with those of SC(4-*O*-8)G (**1**) previously isolated from transgenic *Brassica napus* seeds (Wolfram et al., 2010). UPLC–MS/MS data suggested that there is no detectable amount of the *threo* isomer **1***t* in the Col-0 control seeds. A minor peak (retention time 7.94 min; Figure 4) with MS/MS spectra identical with that of **1***e* may correspond to the *cis-*form of **1***e* because *trans*-cinnamic acid derivatives are known to isomerize to the *cis*-form after exposure to sunlight or UV light (Turner et al., 1993; Yang et al., 1999; Wong et al., 2005), and the ratio of the minor peak increased during the purification process in the light.

**Figure 5:**
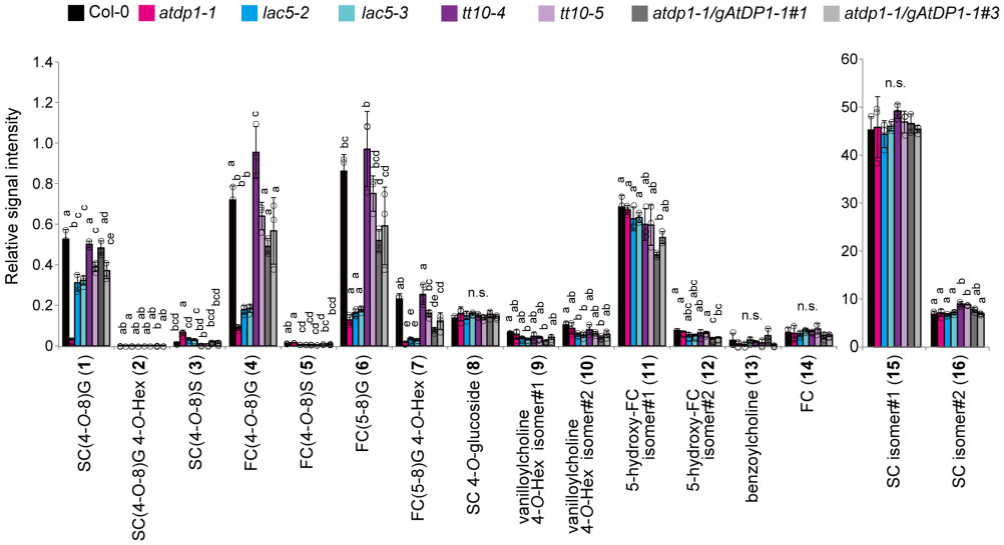
UPLC–QTOF–MS analyses of neolignans and choline derivatives in the mutants. The structures of compounds **1**–**7** are shown in Figure 1. Data represent the means ± sd (three biological replicates per sample). The means were compared by a one-way analysis of variance (ANOVA). Statistically significant differences (*P* < 0.05) were identified by Tukey’s test and are indicated by lowercase letters to represent differences between groups. G, guaiacyl moiety; S, syringyl moiety; SC, sinapoylcholine; FC, feruloylcholine; Hex, hexose; n.s., not significant.

Further comprehensive analyses of neolignans, and SC and FC derivatives suggested that, along with the SC-conjugated neolignan **1***e*, the level of several FC-conjugated neolignans [FC(4-*O*-8)G (**4**), FC(5-8)G (**6**), and FC(5-8)G 4-*O*-Hex (**7**; Figure 1B)] in the *atdp1-1* mutant were significantly less than those in the wild type (Figure 5;  Supplemental Table 1, Supplemental Data Set 2). Accordingly, we chemically synthesized the authentic *erythro*- and *threo*-isomers of neolignan **4**, i.e. *erythro*-FC(4-*O*-8)G **(4***e***)** and *threo*-FC(4-*O*-8)G (**4***t*), respectively, in a manner similar to that described above for the SC(4-*O*-8)G isomers **1***e* and **1***t* (Figure 1C, and also see the Materials and methods section). As a consequence, we established that FC(4-*O*-8)G depleted in the *atdp1-1* mutant was the *erythro* isomer **4***e* (Böttcher et al., 2008; Supplemental Figure 1). As we observed for SC(4-*O*-8)G, the Col-0 seeds exclusively contain the *erythro* isomer **4***e* with no detectable *threo* isomer **4***t* (Supplemental Figure 1). A putative *cis-*form of **4***e* **(**retention time 6.91 min and 6.90 min, Supplemental Figure 1, A, B, and F**)** was also detected. Taken together, our results indicate that AtDP1/AtDIR12 specifically mediates the formation of SC- and FC-conjugated 8-*O*-4′-type neolignans **1***e* and **4***e* in Arabidopsis. In addition, the depletion of FC(5-8)G (**6**) and FC(5-8)G 4-*O*-Hex (**7**) in the *atdp1-1* mutant suggested that AtDP1/AtDIR12 may also be involved in the formation of FC-conjugated 8-5′-type neolignans. Interestingly, the level of SC(4-*O*-8)S (**3**) is higher in the *atdp1-1* mutant (Figure 5;  Supplemental Table 1, Supplemental Data Set 2). This finding suggests that, in the *atdp1-1* mutant, random coupling of SC radicals with CA/sinapyl alcohol radicals may occur and/or excess amounts of substrates such as SC radicals may be utilized by other putative DIR(s) involved in SC(4-*O*-8)S biosynthesis.

### Phylogenetic analysis of AtDP1/AtDIR12

**Figure 6:**
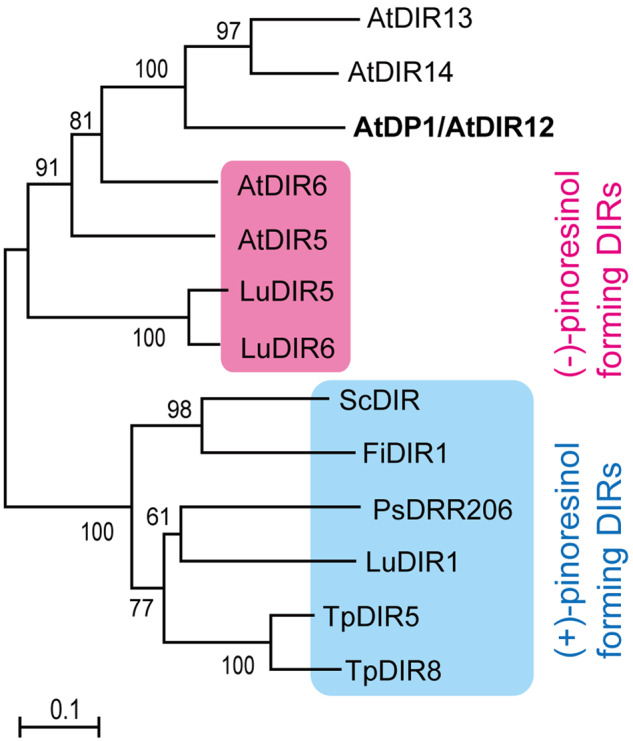
Nonrooted molecular phylogenetic tree of functionally identified DIRs. (+)- and (−)-pinoresinol-forming DIRs are shaded by blue and magenta, respectively. The tree was constructed as described in the Materials and methods section. The alignment used for this analysis is shown in Supplemental Figure 2. Bar = 0.1 amino acid substitutions per site. The GenBank accession numbers for the sequences are shown in parentheses: AtDP1/AtDIR12 (At4g11180, BT004016); AtDIR5 (At1g64160, BT010486); AtDIR6 (At4g23690, BT002439); AtDIR13 (At4g11190, BT015628); AtDIR14 (At4g11210, BT015626); FiDIR1 (AF210061); LuDIR1 (KM433751); LuDIR5 (KM433753); LuDIR6 (KM433752); PsDRR206 (U11716); ScDIR (HQ428029); TpDIR5 (AF210067); TpDIR8 (AF210070). At, *Arabidopsis thaliana*; Fi, *Forsythia intermedia*; Lu, *Linum usitatissimum*; Ps, *Pisum sativum*; Sc, *Schizandra chinensis*; Tp, *Thuja plicata*.

DIRs from *Forsythia intermedia*, western red cedar (*Thuja plicata*), Arabidopsis, *Schizandra chinensis*, pea (*Pisum sativa*), and flax (*Linum usitatissimum*) were previously shown to be involved in the biosynthesis of lignans, (+)- or (−)-pinoresinol, via 8-8′ phenoxy radical coupling and belong to the DIR-a subfamily (Davin et al., 1997; Gang et al., 1999; Kim et al., 2002, 2012, 2015; Ralph et al., 2007; Pickel et al., 2010; Dalisay et al., 2015). The phylogenetic tree of the functionally identified DIRs indicated that these DIRs can be classified into two clusters based on their stereoselectivity, i.e. (+)- and (−)-pinoresinol-forming DIRs; AtDP1/AtDIR12 belongs to the cluster of (−)-pinoresinol-forming DIRs (Figure 6;  Supplemental Figure 2). The sequence identities between (+)- or (−)-pinoresinol-forming DIRs are 57%–67% or 55%–63% at the amino acid level, respectively, except in case of genes from the same plant species. AtDP1/AtDIR12 had a lower sequence identity with (+)- and (−)-pinoresinol-forming DIRs (40%–48% and 47%–50%, respectively, at the amino acid level), but had higher sequence identities with functionally unidentified DIR-a genes (63% and 68% for AtDIR13 and AtDIR14, respectively) and (−)-pinoresinol-forming DIRs (62% and 56% for AtDIR6 and AtDIR5, respectively) from Arabidopsis.

### Structural comparison of AtDP1/AtDIR12 and DIRs for lignan biosynthesis

**Figure 7:**
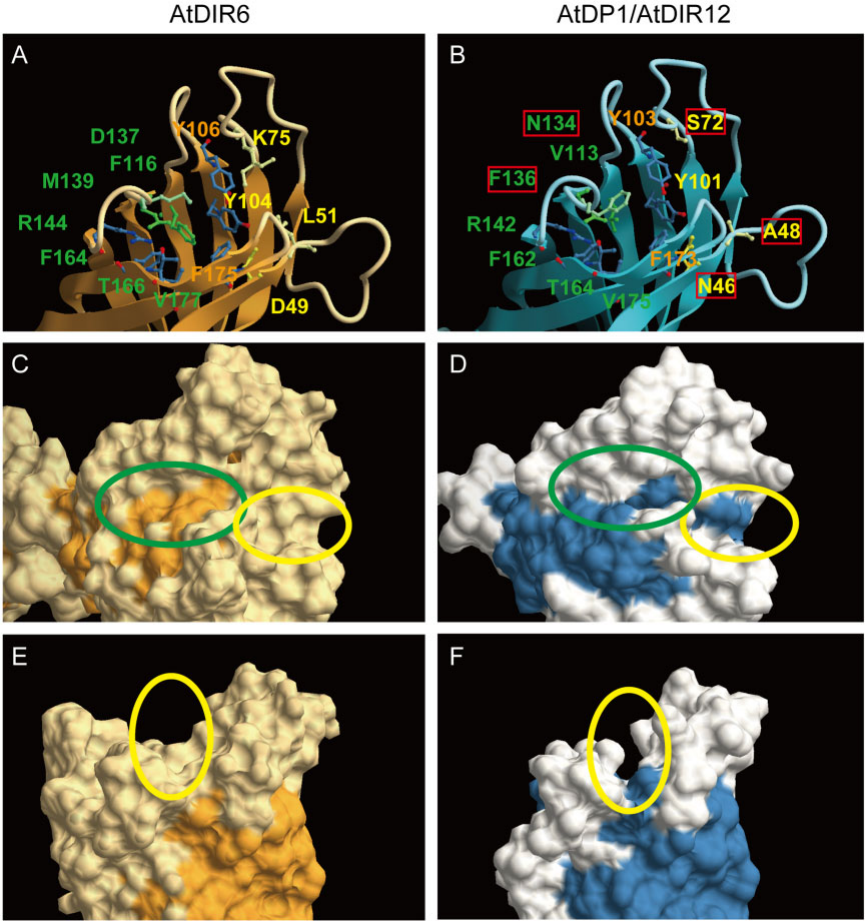
Homology modeling of AtDP1/AtDIR12 based on the crystal structure of AtDIR6. Active site of AtDIR6 (A) and AtDP1/AtDIR12 (B). Amino acids residues for pocket A (green), pocket B (yellow), and the region between the pockets (orange) are shown. Residues unique to AtDP1/AtDIR12 are shown in red boxes. Molecular surface structures of AtDIR6 (C, E) and AtDP1/AtDIR12 (D, F) are shown. Pockets A and B are shown within green and yellow ovals, respectively. Homology modeling was conducted using the SWISS-MODEL Web site (https://swissmodel.expasy.org/) with AtDIR6 (PDB: 5LAL) as a template. CueMol2 was used for the macromolecular structure visualization (http://www.cuemol.org/en/).

Multiple alignments of DIRs suggest the amino acids in AtDP1/AtDIR12 that may partly contribute to catalysis for neolignan biosynthesis. Several amino acid residues unique to AtDP1/AtDIR12 but differently conserved in (+)- and/or (−)-pinoresinol-forming DIRs (Asn46, Ile79, Asn80, Asn104, Val113, Trp115, Asp132, Asn134, Leu154, Lys165, and Cys174) were found (Supplemental Figure 2). Of the five polar residues (corresponding to His39, Asp40, Thr84, Ser91, and Arg141 in PsDRR206) that are highly conserved in both (+)- and (−)-pinoresinol-forming DIRs and lined the edge of the putative substrate binding sites (Kim et al., 2015), four residues (His45, Thr84, Ser91, and Arg142 in AtDP1/AtDIR12) are also conserved, but Asp40 (Asn46 in AtDP1/AtDIR12) is unique to AtDP1/AtDIR12 (Supplemental Figure 2). Crystal structures of a (−)-pinoresinol-forming AtDIR6 showed that active sites are formed by two lobes (pockets A and B; Gasper et al., 2016). Seven of the 13 amino acid residues forming pocket A (Phe116, Asp137, Met139, Arg144, Phe164, Thr166, and Val177), pocket B (Asp49, Leu51, Lys75, and Tyr104), and a region between the pockets (Tyr106 and Phe175) are conserved in AtDP1/AtDIR12 (Gasper et al., 2016; Paniagua et al., 2017; Figure 7). However, two of the six residues proposed to be essential for the activity through hydrogen bond formation or acid catalysis in AtDIR6 (Asp137 and Asp49 of Asp137, Arg144, Thr166, Asp49, Tyr104, and Tyr106) are altered to Asn134 and Asn46 in AtDP1/AtDIR12, respectively. Homology modeling of AtDP1/AtDIR12 based on the AtDIR6 structure showed that substitution of the residues in pocket A (Phe116, Asp137, and Met139 in AtDIR6 to Val113, Asn134, and Phe136 in AtDP1) and pocket B (Asp49, Leu51, and Lys75 in AtDIR6 to Asn46, Ala48, and Ser72 in AtDP1/AtDIR12) conferred a larger and deeper cavity for catalysis (Figure 7; Gasper et al., 2016; Paniagua et al., 2017). Substitutions to Ser72 and Ala48 in AtDP1/AtDIR12 also contribute to slit formation between pockets A and B (Figure 7, E and F). These results may reflect the ability of AtDP1/AtDIR12 to accept bulkier substrates than AtDIR5 and AtDIR6.

### *AtDP1/AtDIR12* is expressed in the outer integument cells of seeds

**Figure 8:**
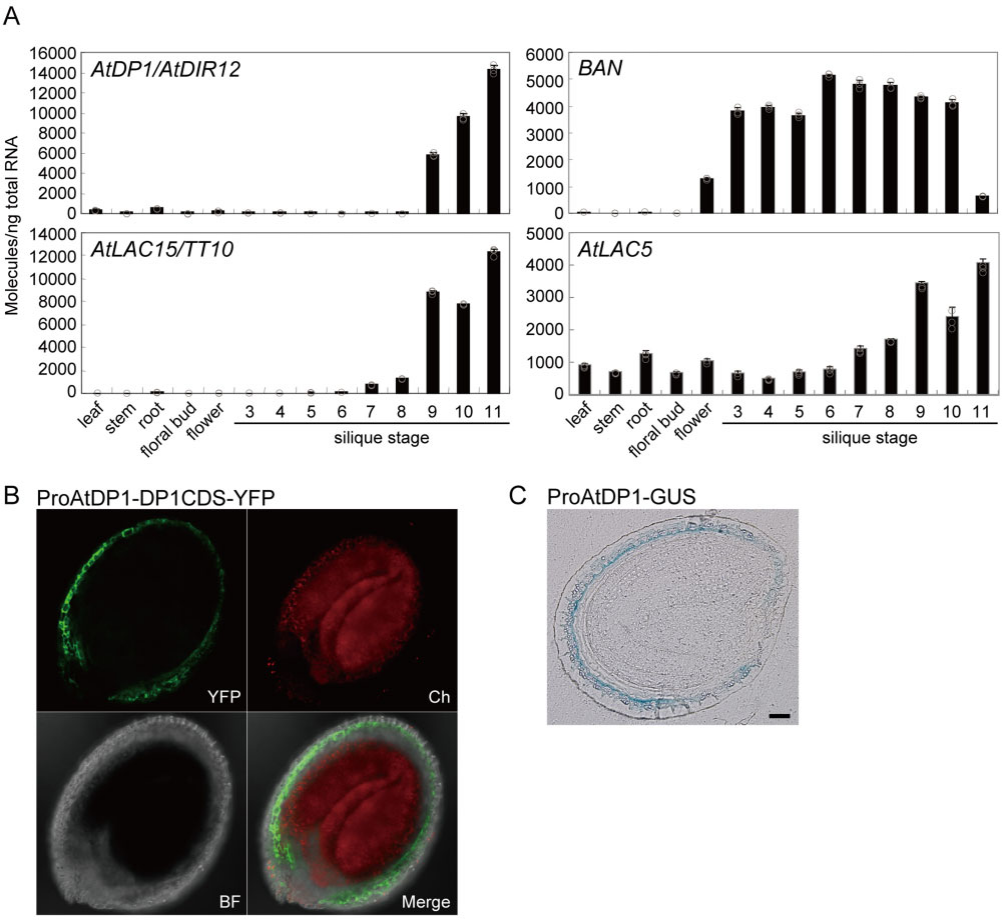
Expression pattern of *AtDP1/AtDIR12*. (A) Transcript abundance of *AtDP1/AtDIR12*, *BAN*, *AtLAC15*/*TT10*, and *AtLAC5* in organs of Arabidopsis wild type (Col-0). Error bars represent sd of three technical replicates per sample. BAN, anthocyanin reductase. (B) Confocal microscopy images depicting the localization of YFP in transgenic plants transformed with a 720-bp promoter of *AtDP1* fused to AtDP1 cDNA with YFP (ProAtDP1-AtDP1CDS-YFP). YFP fluorescence (YFP); chlorophyll autofluorescence (Ch); a bright-field image (BF) and a merged image of the three channels (merge) are shown. Seed stage, 7 days after flowering (corresponding to silique stage 11). (C) Sections (5 µm) of GUS-stained seeds expressing the GUS reporter gene driven by the *AtDP1* promoter. Seed stage, 7 days after flowering (corresponding to silique stage 11). Scale bars = 50 µm.

The accumulation pattern of *AtDP1/AtDIR12* transcripts in various organs including seeds at nine developmental stages was investigated by reverse transcription quantitative PCR (RT-qPCR). *AtDP1/AtDIR12* transcripts predominantly accumulated in seeds at developmental stages 9 to 11, but not in other organs (Figure 8A). These data are consistent with the seed-specific accumulation of *erythro*-SC(4-*O*-8)G (**1***e*) and the expression profile of *AtDP1/AtDIR12* archived on the Arabidopsis eFP browser (http://bar.utoronto.ca/efp/cgi-bin/efpWeb.cgi). The expression profile of anthocyanidin reductase encoded by *BANYULS* (*BAN*), a key enzyme in the proanthocyanidin (PA) biosynthetic pathway, was clearly distinct from that of *AtDP1/AtDIR12* during seed development. Compared to *AtDP1/AtDIR12, BAN* transcripts accumulated in seeds across a broader range of developmental stages (stages 3–10; Figure 8A), suggesting that the two phenylpropanoid-derivative biosynthetic pathways for neolignan and PA are distinctively regulated at the transcriptional level in Arabidopsis seeds.

To assess the cellular localization patterns of AtDP1/AtDIR12 in developing seeds, transgenic plants harboring a 720-bp promoter region of *AtDP1/AtDIR12* fused to *AtDP1/AtDIR12* cDNA with yellow fluorescent protein (YFP; ProAtDP1-AtDP1CDS-YFP) were generated. The YFP fluorescence was specifically observed in the outer integument 1 (oi1) of seeds expressing ProAtDP1-AtDP1CDS-YFP (Figure 8B). Additionally, we generated transgenic plants harboring the 720-bp promoter of AtDP1 fused to GUS (ProAtDP1-GUS). The blue coloration from GUS staining was observed in seed oi1 cells of plants expressing ProAtDP1-GUS (Figure 8C). These data indicate that AtDP1/AtDIR12 localized in oi1 cells of developing seeds in which flavonols also accumulated (Pourcel et al., 2005). TargetP-2.0 (http://www.cbs.dtu.dk/services/TargetP/) predicts the presence of an N-terminal signal peptide in AtDP1/AtDIR12 (likelihood 0.9957), suggesting that AtDP1/AtDIR12 may sort to compartments in the secretory pathway.

### Oxidases for neolignan biosynthesis

Next, we focused on identifying oxidases required for the formation of the monolignol radicals. LACs and/or peroxidases have been suggested to generate the monolignol radicals for lignan/neolignan; however, no specific genes for neolignans were known. Davin et al. reported that a LAC-like oxidase mediated the formation of enantioselective (+)-pinoresinol in the presence of *Forsythia* DIR (Davin et al., 1997; Gang et al., 1999). Therefore, we gave priority to LACs. We hypothesized that the target LAC(s) would need to be simultaneously expressed with *AtDP1/AtDIR12* to perform the function. Among 17 Arabidopsis *LAC*s, positive correlations (*r *>* *0.3) of coordinated expression were noted between *AtDP1/AtDIR12* and two *LAC*s, i.e. *AtLAC15*/*TRANSPARENT TESTA 10* (*TT10*; At5g48100, *r *=* *0.708) and *AtLAC5* (At2g40370, *r *=* *0.399). *AtLAC15*/*TT10* and *AtLAC5* are major *LAC*s expressed in seeds (Schmid et al., 2005). RT-qPCR showed that transcripts of *AtLAC15*/*TT10* and *AtLAC5* are abundant in seeds at developmental stages 9 to 11 as with *AtDP1/AtDIR12*. The abundance of *AtLAC5* transcripts was relatively low compared to those of *AtDP1/AtDIR12* and *AtLAC15*/*TT10* (Figure 8A).

**Figure 9:**
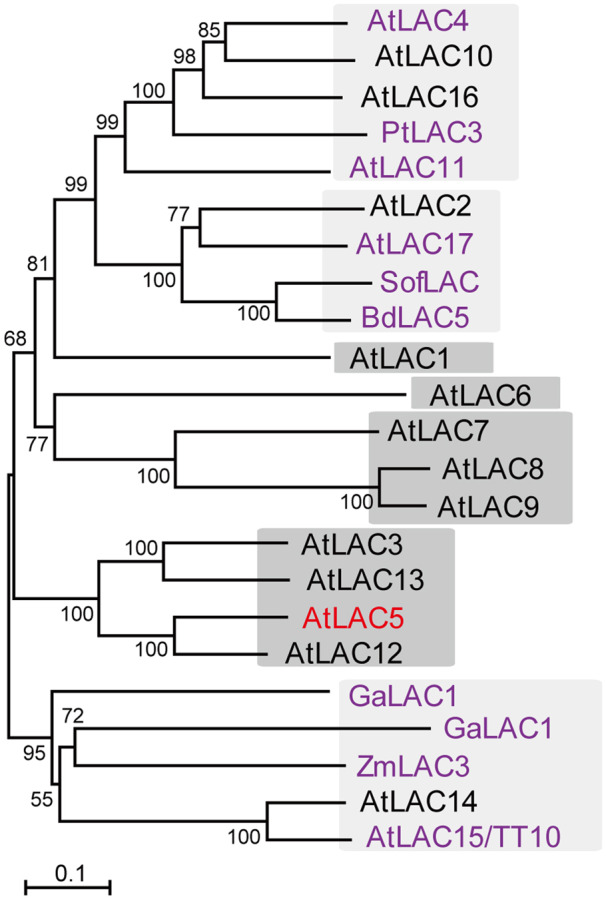
Nonrooted molecular phylogenetic tree of Arabidopsis LACs and functionally identified LACs. Functionally identified LACs are shown in purple. The clusters with unknown LACs only are shown with a dark grey background. The tree was constructed as described in Materials and methods section. Bar = 0.1 amino acid substitutions per site. The GenBank accession numbers for the sequences are shown in parentheses: AtLAC1 (At1g18140, NP_173252); AtLAC2 (At2g29130, NP_180477); AtLAC3 (At2g30210, NP_180580); AtLAC4 (At2g38380, NP_565881); AtLAC5 (At2g40370, NP_181568); AtLAC6 (At46570, NP_182180); AtLAC7 (At3g09220, NP_187533); AtLAC8 (At5g01040, NP_195724); AtLAC9 (At5g01050, NP_195725); AtLAC10 (At5g01190, NP_195739); AtLAC11 (At5g03260, NP_195946); AtLAC12 (At5g05390, NP_196158); AtLAC13 (At5g07130, NP_196330); AtLAC14 (At5g09360, NP_196498); AtLAC15 (At5g48100, NP_199621); AtLAC16 (At5g58910, NP_200699); AtLAC17 (At5g60020, NP_200810); BdLAC5 (Bradi1g66720, XP_003558240); BnTT10 (NP_001302959); GaLAC1 (NP_001316972); PtLAC3 (CAC14719); SofLAC (SCVPRZ3027A08.g and SCUTST3084C11.g); ZmLAC3 (CAJ30499). At, *Arabidopsis thaliana*; Bd, *Brachypodium distachyon*; Bn, *Brassica napus*; Ga, *Gossypium arboreum*; Pt, *Populus trichocarpa*; Sof, *Saccharum officinarum*; *Zm, Zea mays.* The alignment used for this analysis is shown in Supplemental Figure 3.

In Arabidopsis, AtLAC15/TT10 is involved in the oxidative polymerization of flavonoids/PAs and lignins (Pourcel et al., 2005; Liang et al., 2006). To date, several plant *LAC*s have been functionally identified as LACs that polymerize lignin (*AtLAC4*, *AtLAC11*, and *AtLAC17*; Berthet et al., 2011; Zhao et al., 2013; Tobimatsu and Schuetz, 2019), PAs and/or phenolic compounds (*BnTT10*, *GaLAC1*, *PtLAC3*, *SofLAC*, and *ZmLAC3*; Ranocha et al., 2002; Caparros-Ruiz et al., 2006; Wang et al., 2008; Cesarino et al., 2013; Zhang et al., 2013). Phylogenetic comparison of the 17 *AtLAC*s with functionally identified *LAC*s from other plants showed that *AtLAC15/TT10* belongs to a cluster that includes *BnTT10*, *ZmLAC3*, and *GaLAC1*. *AtLAC5* belongs to another cluster with *AtLAC*s of unknown function that includes *AtLAC3*, *AtLAC12*, and *AtLAC13* (Figure 9;  Supplemental Figure 3).

To elucidate the function of *AtLAC15*/*TT10* and *AtLAC5*, T-DNA inserted knockout lines for *AtLAC15*/*TT10*, SALK_002972 (*tt10-4*), and SALK_128292 (*tt10-5*; Pang et al., 2013) and those for *AtLAC5*, SALK_092440 (*lac5-2*), and SALK_093534 (*lac5-3*), were isolated. No transcripts of *AtLAC5* in the *lac5-2* and *lac5-3* lines were found by RT-PCR analysis (Supplemental Figure 4).

The analyses of neolignans, and SC and FC derivatives showed that the abundance of FC-conjugated neolignans [*erythro*-FC(4-*O*-8)G (**4***e*), FC(5-8)G (**6**), and FC(5-8)G 4-*O*-Hex (**7**; Figure 1B;  Supplemental Figure 1)] in the *lac5* mutants was as low as those in the *atdp1-1* mutant (Figure 5;  Supplemental Table 1, Supplemental Data Set 2). Interestingly, the abundance of *erythro*-SC(4-*O*-8)G (**1***e*) in the *lac5* mutants may be slightly lower than that in wild type, but the accumulation level was much higher than that in the *atdp1* mutant. Neolignans in *tt10* mutants showed no significant changes in abundance. The content of choline derivatives (**8–16**, Figure 5) was similar in wild type, and the *lac5* and *tt10* mutants. These data suggested that AtLAC5 is predominantly involved in the formation of FC-conjugated neolignans, despite the linkage types between monolignol and choline derivatives, but is not required for SC-conjugated neolignan biosynthesis.

### Localization of *AtLAC5* in seeds

**Figure 10:**
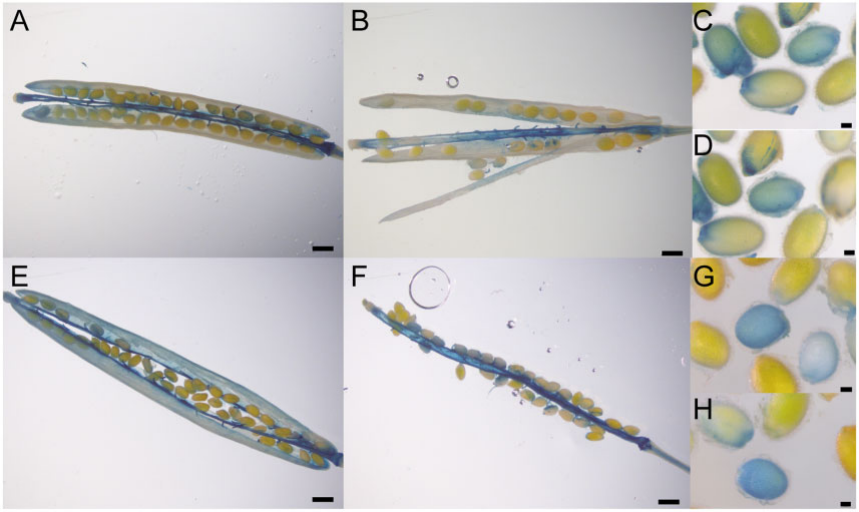
Expression pattern of *AtLAC5*. GUS staining of transgenic plants harboring the 4,140-bp (A–D) or 2,000-bp (E–H) promoter region of *AtLAC5* fused to GUS. Siliques at 7 (B), 8 (F), 9 (A and E) days after flowering and immature seeds at 10 days after flowering (C, D, G, and H) were used for the analyses. Scale bars=0.64 mm (A, B, E, and F) or 0.1 mm (C, D, G, and H).

Experiments using the *AtLAC5* promoter (2,358 bp)-GUS fusion construct suggested that AtLAC5 is localized in the replum and abscission zone in siliques and seed embryos (Turlapati et al., 2011). As the *AtLAC5* promoter region was estimated to be 4,140 bp (Turlapati et al., 2011), we transformed plants with constructs containing either the 4,140-bp or a 2,000-bp promoter region of *AtLAC5* fused to GUS (ProAtLAC5-4140-GUS, ProAtLAC5-2000-GUS). The blue coloration by GUS staining was predominantly observed in the replum, septum, and funiculus and to a lesser extent in the valves of transgenic plants, using either promoter (Figure 10, A, B, E, and F). Staining of the seeds was also observed but it was not clear (Figure 10, C, D, G, and H).

TargetP-2.0 (http://www.cbs.dtu.dk/services/TargetP/) predicts the presence of an N-terminal signal peptide in AtLAC5 (likelihood 0.9998), suggesting that AtLAC5 may sort to compartments in the secretory pathway as is also the case for AtDP1/AtDIR12. Transcriptome data obtained from the Arabidopsis eFP browser showed that *AtLAC5* transcripts accumulate exclusively in seeds and particularly in seed coats of mature green seeds (Supplemental Figure 5). The Arabidopsis Seed Coat eFP Browser showed that the accumulation level of *AtLAC5* transcripts in *ap2-7* mutants is lower compared to wild-type (Supplemental Figure 5). AP2 is a transcription factor required for differentiation of the epidermal and subepidermal palisade layers of the outer integument corresponding to the oi2 and oi1 cell layers, respectively (Western et al., 2001; Dean et al., 2011). These data suggest that *AtLAC5* is expressed in the outer integument of seeds. The accumulation level of *AtDP1* transcripts in the *ap2-7* mutants is also lower than that of wild-type (Supplemental Figure 5). The Arabidopsis Seed Coat eFP browser also suggests that *AtDP1/AtDIR12* is expressed earlier than At*LAC5*, an observation that is somewhat contradictory to our RT-qPCR data showing that the expression of *AtLAC5* is similar to that of *AtDP1/AtDIR12* (Figure 8). AtDP1/AtDIR12 may be ready for precise radical coupling prior to the appearance of AtLAC5 or tissue dissection may trigger additional *AtDP1/AtDIR12* expression.

### The biosynthetic pathway for SC/FC-conjugated neolignans

**Figure 11:**
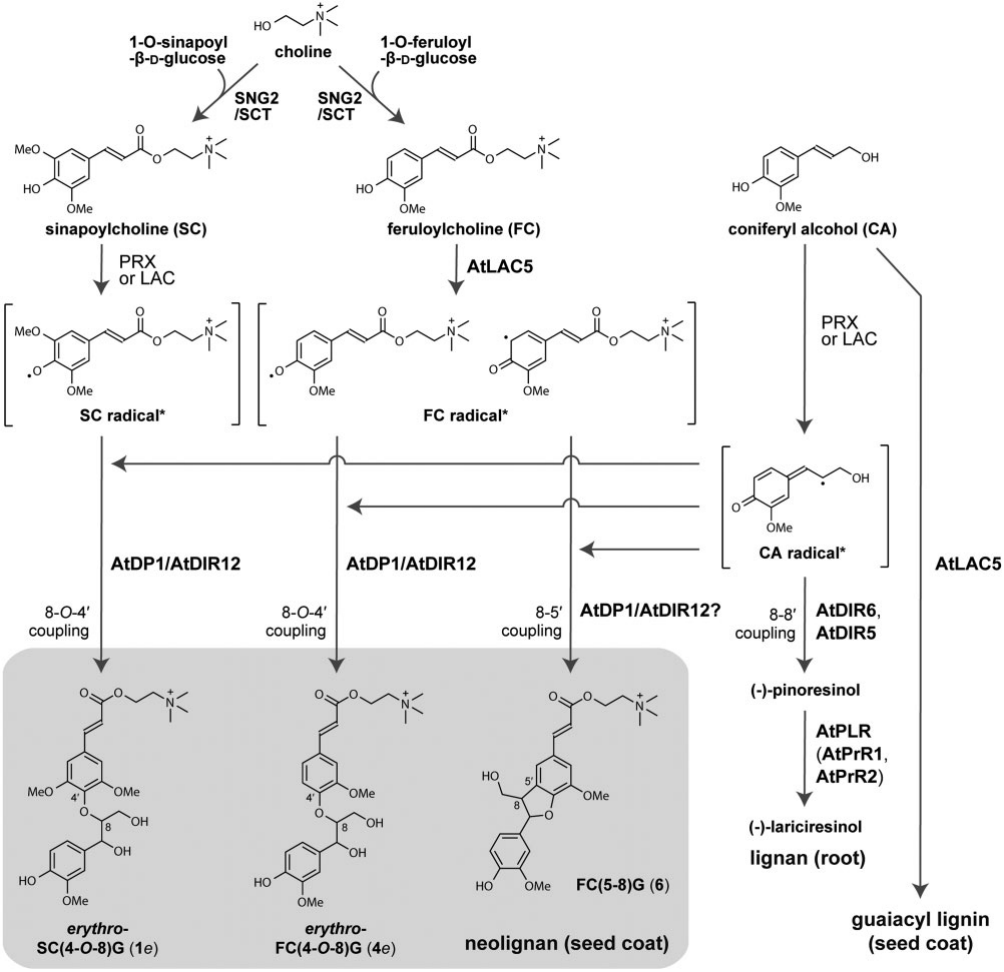
The proposed biosynthetic pathways of neolignans, lignans, and lignins in Arabidopsis. Asterisks indicate the reader’s convenience, each phenolic radical is depicted with its resonance form chosen to allow the radical coupling, 8-*O*-4′ and 8-5′, to be rationalized; the two FC^●^ structures shown, for example, are simply two resonance forms of the same phenolic radical. DIR, dirigent protein; laccase, LAC; PRX, peroxidase; PLR, pinoresinol/lariciresinol reductase; PrR, pinoresinol reductase; G, guaiacyl moiety; SC, sinapoylcholine; FC, feruloylcholine; CA, coniferyl alcohol.

**Figure 12:**
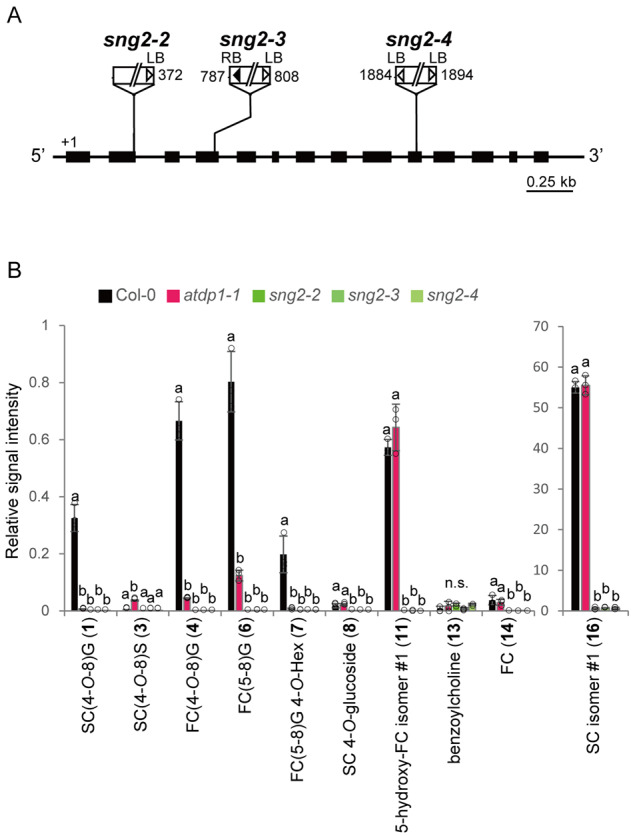
T-DNA insertion mutants of *SCT*, *sng2.* (A) Schematic representation of *SCT* showing the three T-DNA insertion mutants used in this study. The thick line indicates the coding region and the thinner line indicates the 5′- and 3′-untranslated regions. White and black triangles show the left and right borders, respectively. Numbers indicate the position of the T-DNA insertion. LB, left border; RB, right border. (B) UPLC–QTOF–MS analyses of neolignans and choline derivatives in the mutants. The structures of compounds (**1, 3, 4, 6–8, 11, 13, 14, 16**) are shown in Figure 1. Data represent the means ± sd (three biological replicates per sample). The means were compared by a one-way ANOVA. Statistically significant differences (*P* < 0.05) were identified by Tukey’s test and are indicated by lowercase letters to represent differences between groups. G, Guaiacyl moiety; S, Syringyl moiety; SC, sinapoylcholine; FC, feruloylcholine; Hex, hexose; n.s., not significant.

To date, there has been almost no information about the biosynthetic pathway for SC- and FC-conjugated neolignans. As a possible route to synthesize SC- and FC-conjugated neolignans, it is conceivable that choline derivatives (SC and FC) may directly couple with CA via 8-*O*-4′ or 8-5′ couplings (Figure 11). SC is synthesized by sinapoylglucose: choline sinapoyltransferase (SCT), SCT/SCPL19 (At5g09640), an enzyme that transfers a sinapoyl moiety from 1-*O*-sinapoyl-β-d-glucose to choline (Shirley et al., 2001). SCT/SCPL19 also has a feruloylglucose: choline feruloyltransferase activity (Shirley and Chapple, 2003). A mutant defective in the *SCT/SCPL19* locus was designated as *sinapoylglucose accumulator 2* (*sng2*). SC levels are decreased and levels of 1-*O*-sinapoyl-β-d-glucose and choline are increased in ethyl methanesulfonate-induced *sng2* mutant seeds (Shirley et al., 2001). We isolated two homozygous *sng2* T-DNA insertion mutants, *sng2-3* (SALK_053495C) and *sng2-4* (SALK_018120C) to compare with a known *sng2-2* mutant (SALK_002255C; Lee et al., 2012; Figure 12;  Supplemental Data Set 3). The T-DNA in *sng2-3* and *sng2-4* was inserted in the exon of *SNG2* but between positions +787 to +808 and +1884 to +1894 base pairs, respectively, from the start codon of SCT/SCPL19. The analyses of neolignans and choline derivatives showed that SC- and FC-conjugated neolignans were clearly missing in the three *sng2* mutant seeds as with SC and FC (Figure 12;  Supplemental Data Set 3). On the other hand, the accumulation level of benzoylcholine in the three *sng2* mutants was similar to that in Col-0. This finding is consistent with the previously published data using the *sng2-2* mutant (Lee et al., 2012). Collectively, these data support our notions that SCT/SCPL19 also functions as a feruloylglucose: choline feruloyltransferase *in planta*, and SC/FC are coupled to CA to yield the SC- and FC-conjugated neolignans (Figure 11).

### Neolignans affect seed coat permeability

Phenolic compounds such as flavonoids are known to be antioxidants that can prevent the generation of free radicals and subsequent oxidative damage to cells (Sano et al., 2016). Arabidopsis *transparent testa* mutants that are unable to synthesize flavonoids, including PAs, showed a significant increase in seed coat permeability and reduced germination capacity during long-term seed storage (Debeaujon et al., 2000).

**Figure 13:**
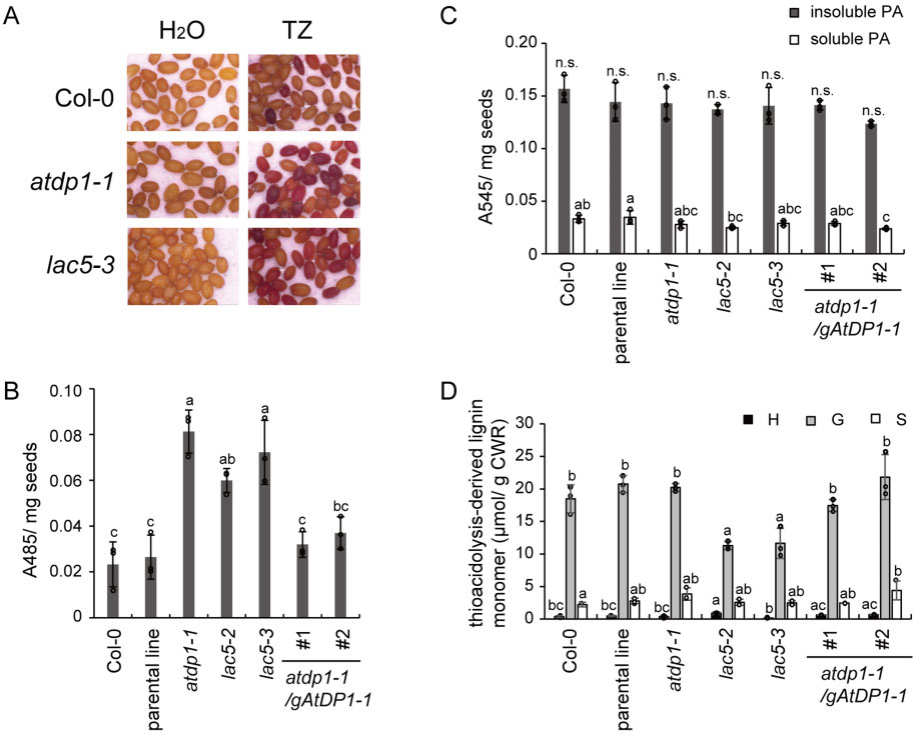
Seed coat permeability of *atdp1* and *lac5* mutants. Seed coat permeability was tested by measuring the conversion of tetrazolium salts to red products called formazans. (A) Seeds of wild type (Col-0) and the mutants (*atdp1-1* and *lac5-3*) stained in a tetrazolium salt (TZ) solution or H_2_O after 48 h incubation. (B) The absorbance of ethanol extracts containing formazan from the seeds stained with tetrazolium salts at 485 nm. The intensity of A_485_ is directly proportional to the permeability of the seed coat. (C) Soluble and insoluble proanthocyanidins were analyzed by acid hydrolysis as described in Materials and methods section. No significant difference (*P* < 0.05) was detected for the insoluble proanthocyanidins. (D) The yield of thioacidolysis-derived *p*-hydroxyphenyl (H)-, guaiacyl (G)-, and syringyl (S)-type trithioethylpropane monomers released from H-, G-, and S-type lignin polymer units. Data represent the means ± sd (three biological replicates per sample). The means were compared by a one-way ANOVA. Statistically significant differences (*P* < 0.05) were identified by Tukey’s test and are indicated by lowercase letters to represent differences between groups. n.s., not significant; CWR, cell wall residue.

To investigate the effect of decreasing the neolignan content of seeds, we tested the permeability of seed coats by the tetrazolium penetration assay (Berridge et al., 1996; Vishwanath et al., 2013, 2014). Tetrazolium salts are colorless compounds, but they are converted to red products called formazans by endogenous NADH-dependent reductases after they penetrate the seed coat (Berridge et al., 1996). The intensity of red coloration is directly proportional to the seed coat permeability (Vishwanath et al., 2014). Quantitative analyses by extraction of the formazans in 95% ethanol showed that accumulation of formazans in *atdp1-1* was the highest and that accumulation in *lac5-2*, *lac5-3*, wild-type, and the parental line was 73.7% ± 6.4%, 88.8% ± 17.2%, 28.7% ± 12.2%, 32.5% ± 11.8% of *atdp1-1*, respectively (Figure 13B;  Supplemental Data Set 4). The permeability of the *atdp1-1* mutant complemented with genomic At*DP1*/*AtDIR12* was similar to that of the wild-type.

We also analyzed the PAs and lignin content to test the possibility that loss of function of AtDP1/AtDIR12 or AtLAC5 might also affect the levels of these compounds, thereby resulting in higher seed coat permeability in the mutants. The PA content as determined by the butanol-HCl assay (Tohge et al., 2005; Kitamura et al., 2010) showed no significant difference between Col-0 and the *atdp1* and *lac5* mutants in which the neolignan content was substantially lower than wild type (Figure 13C;  Supplemental Data Set 4). The relative lignin content of Col-0 and the mutants was evaluated by the yield of lignin monomers released by analytical thioacidolysis, a reaction that cleaves the quantifiable lignin monomers by chemical cleavage of the major β-*O-*4 linkages in lignin polymers (Lapierre et al., 1986). Consequently, we detected no lignin reduction in *atdp1-1*, supporting our notion that the decrease in the neolignan content primarily led to the high permeability of the seed coat in this mutant (Figure 13D;  Supplemental Data Set 4). Additionally, the two *lac5* mutants (*lac5-2* and *lac5-3*), also had reduced yields of guaiacyl-type lignin monomers, whereas yields of syringyl-type lignin monomers in the *lac5* mutants were similar to those of wild type (Figure 13D;  Supplemental Data Set 4). This result suggests that AtLAC5 may be involved not only in FC-conjugated neolignan biosynthesis but also in guaiacyl-type lignin biosynthesis in Arabidopsis seeds. This result supports the hypothesis that the high permeability of the *lac5* seed coats may be attributed to a reduced content of both neolignan and lignin.

## Discussion

### AtDP1/AtDIR12 is essential for neolignan biosynthesis

Our results demonstrated that AtDP1/AtDIR12 is essential for the biosynthesis of SC- and FC-conjugated 8-*O*-4′-type neolignans in Arabidopsis (Figures 3, 5). Although the absolute configurations of the AtDP1/AtDIR12-derived neolignans **1***e* and **4***e* (Figure 1C), i.e. whether they have (8*S*, 7*R*) and/or (7*S*, 8*R*) configurations, are yet to be determined, it is plausible that AtDP1/AtDIR12 guides the regio- and stereo-selective 8-*O*-4′ coupling of CA with SC and FC to form optically active products. Intriguingly, the wild-type seeds appeared to accumulate the *erythro* isomers **1***e* and **4***e* exclusively with no detectable amounts of the corresponding *threo* isomers **1***t* and **4***t* (Figure 4;  Supplemental Figure 1). This observation strongly suggests that AtDP1/AtDIR12 may guide not only the radical coupling step but also that in the subsequent rearomatization step in which the quinone methide intermediates are attacked by water during the formation of neolignans **1***e* and **4***e*. This intriguing stereochemical mechanism, including which enantiomer of *erythro* compounds is accumulated and how these reactions are mediated by AtDP1/AtDIR12, should be further addressed together with a detailed three-dimensional protein structure.

To date, several DIRs involved in the stereoselective (enantioselective) 8-8′ coupling of CA to produce lignan, (+) or (−)-pinoresinol, have been identified (Davin et al., 1997; Kim et al., 2002, 2012, 2015; Ralph et al., 2007; Pickel et al., 2010; Dalisay et al., 2015), but there have been no reports of DIRs involved in the 8-*O*-4′ couplings of monolignols or their analogs for neolignan biosynthesis. Of the six DIR subfamilies, AtDP1/AtDIR12 belongs to the DIR-a family with other DIRs involved in lignan biosynthesis. Phylogenetic analysis of functionally identified DIR-a genes revealed that DIR-a can be classified into two clusters based on their stereoselectivity, (+)- and (−)-pinoresinol-forming DIRs. AtDP1/AtDIR12, AtDIR13, and AtDIR14 belong to a cluster of (−)-pinoresinol-forming DIRs that include AtDIR6 and AtDIR5 (Figure 6). Evolutionary relationships of the Arabidopsis DIR-a genes proposed by Wang et al. (2013) suggest that AtDIR5 was an ancestral gene and AtDP1/AtDIR12 and AtDIR6 were derived from AtDIR5 by the whole-genome duplication event β or AtDP1/AtDIR12 was derived from AtDIR6 by the whole-genome duplication event α (Supplemental Figure 6; Wang et al., 2013). These data suggest that AtDP1/AtDIR12 may have diverged from (−)-pinoresinol-forming DIRs.

### The neolignan biosynthetic pathway in Arabidopsis

Our results using the *sng2* mutants that are defective in the ability to synthesize SC revealed that SC- and FC-conjugated neolignans are synthesized from SC/FC and CA, and AtDP1/AtDIR12 is essential for producing the 8-*O*-4′-type neolignans, *erythro*-SC(4-*O*-8)G (**1***e*) and *erythro*-FC(4-*O*-8)G (**4***e*), in Arabidopsis seeds. Notably, in seeds of the *atdp1-1* mutant, the abundance of other neolignans putatively annotated as FC-monolignol conjugate FC(5-8)G (**6**) and its glycoside FC(5-8)G 4-*O*-Hex (**7**; Figure 1B) were also coordinately reduced (Figure 5), suggesting that AtDP1/AtDIR12 may be also able to guide the 8-5′-coupling of monolignol radicals with FC radicals (Figure 11).

Arabidopsis contains derivatives of lignans, neolignans, and oligolignols in leaves/shoots and lignan derivatives in roots (Nakatsubo et al., 2008; Okazawa et al., 2011; Dima et al., 2015). In leaves, 8-*O*-4′- and 8-5′-type neolignans composed of ferulic acid/feruloyl malate/sinapoyl malate and monolignols have been identified (Dima et al., 2015). In the inflorescence stem of Arabidopsis, a pathway to produce feruloylmalate(4-*O*-8)G from ferulic acid and CA via ferulic acid(4-*O*-8)G and feruloylhexose(4-*O*-8)G has been suggested (Vanholme et al., 2012). Given that AtDP1/AtDIR12 is specifically expressed in seeds, other DIR(s) may guide 8-*O*-4′ and/or 8-5′ radical coupling of monolignols and sinapoyl/feruloyl derivatives in organs other than seeds. The enzyme responsible for the final step in the synthesis of sinapoylmalate, sinapoylglucose:malate SMT, was identified in Arabidopsis previously (Lehfeldt et al., 2000). Future reciprocal studies focusing on chemical characterization and genetic identification of other AtDIRs and the neolignan analyses of the *smt* mutants will further elucidate the neolignan biosynthetic pathway in Arabidopsis.

In addition, a variety of (neo)lignans, including aglycones and glycosides, are reportedly stored in Arabidopsis leaf vacuoles (Dima et al., 2015). On the other hand, in flaxseed (*Linum usitatissimum*), lignans are mainly localized in the secondary walls of sclerite cells in the seed outer integument (Attoumbre et al., 2010). AtDP1/AtDIR12, AtLAC5, and SCT/SCPL19 that are all involved in seed neolignan biosynthesis were predicted to have signal peptides, suggesting that these proteins may be secreted and sorted into compartments such as vacuoles, tonoplasts, plasma membranes, and cell walls by the secretory pathway; however, the intracellular localization patterns of AtDP1/AtDIR12, AtLAC5, and SCT/SCPL19 are unknown. All three flaxseed DIRs involved in lignan biosynthesis, LuDIR1, LuDIR5, and LuDIR6, also have signal peptides (Corbin et al., 2018). In *Forsythia intermedia* stems, DIR(s) are localized to the cell wall (Burlat et al., 2001). Arabidopsis LACs for lignin biosynthesis, AtLAC4 and AtLAC17, are reportedly localized to secondary cell walls (Schuetz et al., 2014), and AtLAC15/TT10 for proanthocyanidin biosynthesis is localized to vacuoles (Pang et al., 2013). Serine carboxypeptidase-like (SCPL) proteins are known to localize in vacuoles (Hause et al., 2002; Mugford et al., 2013). Further investigation is required to elucidate the intracellular localization of AtDP1/AtDIR12, AtLAC5, and SCT/SCPL19 and the sites for neolignan biosynthesis and storage.

### AtLAC5 is required for the biosynthesis of FC-conjugated neolignans

Peroxidases and/or LACs have been reported to be probably involved in monolignol oxidation in lignin and lignan biosynthesis, but no oxidases for neolignan biosynthesis have been identified. Here, we revealed that AtLAC5 is also essential for neolignan biosynthesis. Interestingly, AtLAC5 is involved in the synthesis of the FC-conjugated 8-*O*-4′-type as well as the 8-5′-type neolignans but is not involved in the synthesis of SC-conjugated neolignans (Figure 5). This finding suggests that AtLAC5 is responsible for the oxidation of FC but not for SC; other oxidases may oxidize SC to form SC-conjugated neolignans with CA. LACs are frequently able to catalyze the oxidation of a wide range of substrates *in vitro* (Sterjiades et al., 1992; Bao et al., 1993); however, the substrate specificity may be more strict *in vivo*. A seed coat-specific LAC from *Cleome hassleriana*, ChLAC8, is capable of oxidizing caffeoyl alcohol and sinapyl alcohol but not CA (Wang et al., 2020). This finding is consistent with our hypothesis.

In addition, our lignin analysis data based on thioacidolysis suggested that AtLAC5 is also involved in the oxidation of CA to form guaiacyl-type lignins but is not involved in the oxidation of sinapyl alcohol for syringyl-type lignins in Arabidopsis seed coats (Figure 13D). These data suggest that AtLAC5 preferentially oxidizes guaiacyl-type substrates, e.g. FC and CA, over syringyl-type substrates, e.g. SC and sinapyl alcohol, although further biochemical studies are needed to support this hypothesis.

The *in vitro* assays using developing seeds showed that *tt10* mutant seeds had a reduced polymerization activity to produce possible 8-*O*-4′, 8-8′, and/or 8-5′ dimers of CA compared to wild-type seeds (Pourcel et al., 2005; Liang et al., 2006). TT10 is localized in oi1 cells (Pourcel et al., 2005); however, no significant changes in the neolignan content were observed in the *tt10* (*atlac15*) mutants. Our results indicated that TT10/AtLAC15 is not involved in neolignan biosynthesis and suggest that the substrate specificity of TT10/AtLAC15 may be also strict *in vivo*, or TT10/AtLAC15 may be separated spatially from monolignols in the cells.

### Physiological roles of neolignans in Arabidopsis seeds

*AtDP1/AtDIR12* is specifically expressed in seeds. Analyses using transgenic plants harboring an *AtDP1/AtDIR12* promoter fused to *AtDP1/AtDIR12* cDNA with YFP or GUS showed that AtDP1/AtDIR12 is localized in the oi1 cells of developing seeds (Figure 8). These results suggest that AtDP1/AtDIR12-guided neolignans are synthesized in oi1 cells that surround the embryo. The tetrazolium salt assay showed that higher seed coat permeability was observed in the *atdp1-1* mutant that is deficient in neolignans (Figure 5) but has comparable accumulation levels of PAs and lignins as the wild type (Figure 13). This result suggests that neolignans play a role in protecting seeds against environmental factors. Furthermore, considering the remarkable biological activities of neolignans and lignans as toxins and deterrents for insects and microorganisms (Nitao et al., 1992; Schroeder et al., 2006; Kulik et al., 2014), neolignans in Arabidopsis seeds may also contribute to chemical defense against herbivores and pathogenic microorganisms by their toxicity.

How neolignans affect seed coat permeability is unknown. Arabidopsis mutants unable to synthesize PAs also had a significant increase in seed coat permeability to tetrazolium salts (Debeaujon et al., 2000). PAs accumulate in the innermost layer of the inner integument (endothelium or inner integument 1 layer; Debeaujon et al., 2003). Seeds are firmly protected by phenolic compounds such as neolignans and PAs in at least two cell layers. These compounds, along with lignin in testa cell walls, may form the barriers to penetration.

Our findings herein shed light on new aspects of DIRs and LACs in neolignan biosynthesis. By regulating the stereoselective radical coupling of phenolic compounds, DIRs and LACs are able to produce structurally diverse, specialized phenolic metabolites for chemical defense.

## Materials and methods

### Plant materials

*Arabidopsis thaliana* (accession Columbia-0, Lehle seeds) was used as the wild type in this study. The T-DNA inserted mutants, SAIL_60_D04 (*atdp1-1*) and SALK_062238 (*atdp1-2*) for *AtDP1/AtDIR12*, SALK_002972 (*tt10-4*) and SALK_128292 (*tt10-5*) for *AtLAC15/TT10*, SALK_092440 (*lac5-2*) and SALK_093534 (*lac5-3*) for *AtLAC5*, SALK_002255C (*sng2-2*), SALK_053495C (*sng2-3*), and SALK_018120C (*sng2-4*) for *SNG2* were obtained from the Arabidopsis Biological Resource Center (ABRC, https://abrc.osu.edu/). As the parental line of SAIL_60_D04 (*atdp1-1*), CS8846 was used for comparison. T-DNA insertion lines, SALK_062238 (*atdp1-2*), SALK_092440 (*lac5-2*), SALK_093534 (*lac5-3*), SALK_053495C (*sng2-3*), and SALK_018120C (*sng2-4*) were screened by PCR using specific primers for *AtDP1/AtDIR12* (At4g11180-6818953f and At4g11180-6819803r), *AtLAC5* (At2g40370/886f and At2g40370/1826r), *SNG2* (At5g09640_SCPL19_-8F and At5g09640_SCPL19_2519R), and T-DNA (LBa1 and RBa1; Supplemental Table 2). PCR products were sequenced to determine the exact insertion points. RT-qPCR was performed as described previously (Yonekura-Sakakibara et al., 2007) with primers At4g11180-14f and At4g11180-607r for the *atdp1* mutants, and At2g40370/875f and At2g40370/1826r for the *lac5* mutants (Supplemental Table 2). Homozygous knockout lines of SAIL_60_D04 (*atdp1-1*), SALK_002972 (*tt10-4*), SALK_128292 (*tt10-5*), and SALK_002255C (*sng2-2*), were isolated as described previously (Matsuda et al., 2010; Lee et al., 2012; Pang et al., 2013). Plants were grown in soil at 22°C under 16/8 h light (40–75 μmol m^−2^ s^−1^ of fluorescent lamp)/dark cycle.

### Evaluation of T-DNA insertion mutants

For complementation tests, ca. 3.0-kb and 1.9-kb genomic fragments covering 1.8 kb and 0.72 kb of the promoter region, the entire *AtDP1/AtDIR12* coding region and 0.6 kb of the 3′-noncoding region were amplified by PCR using primers, gDP1-Rv2 and CACC-gDP1-Fw1 or CACC-gDP1-Fw2 (Supplemental Table 2). Amplified fragments were cloned into the pENTR/D-TOPO vector (Invitrogen) as the entry vector and sequenced to confirm the absence of PCR errors. pGWB1 was used as the destination vector, and the LR reactions for the binary vectors *gDP1-1* and *gDP1-2* were catalyzed using the Gateway LR Clonase^TM^II enzyme mix (Invitrogen). *gDP1-1* (pGWB1/3.0-kb *AtDP1/AtDIR12* genomic fragment, pSPB3566) and *gDP1-2* (pGWB1/1.9 kb *AtDP1/AtDIR12* genomic fragment, pSPB3567) were transformed into *Agrobacterium tumefaciens* strain EHA105, and Arabidopsis plants were transformed by the floral-dip method (Clough and Bent, 1998). T_2_ plants were selected on half-strength Murashige and Skoog medium containing 50 µM glufosinate ammonium (Sigma cat#45520), 50 mg L^−1^ kanamycin sulfate, and 50 mg L^−1^ carbenicillin, sodium salt.

#### Neolignan analysis using LC–ESI–Q-Tof–MS

The seeds (3.0 mg) were homogenized in 50 volumes of the extraction solvent, 50% (v/v) MeOH with 0.5-mg L^−1^ lidocaine, and 10-camphor sulfonic acid, using a mixer mill (MM300, Retsch) with zirconia beads for 10 min at 20 Hz. After centrifugation at 15,000*g* and filtration (HLB µElution plate, Waters), the extracts (3 µL) were applied to a liquid chromatography (LC)–mass spectrometry (MS) system with an electrospray ionization (ESI) interface (LC, Waters Aquity UPLC system; MS, Waters Q-Tof Premier). For analyses of the putative neolignan compounds (*m*/*z *=* *668 or 506), LC system conditions (positive analysis) were as described previously (Matsuda et al., 2009).

For analyses of neolignans and choline derivatives, the dried samples were extracted with 50 µL of 80% (v/v) MeOH containing 2.5 mM lidocaine and 10-camphor sulfonic acid per mg dry weight using a mixer mill with zirconia beads for 7 min at 18 Hz and 4°C. After centrifugation for 10 min, the supernatant fraction was filtered using an HLB µElution plate (Waters). The extracts (1 µL) were analyzed using an LC–Q-Tof–MS (LC, Waters Acquity UPLC system; MS, Waters Xevo G2 Q-Tof). Analytical conditions were as follows: LC column, Acquity bridged ethyl hybrid (BEH) C18 (1.7 µm, 2.1 mm × 100 mm, Waters); solvent system, solvent A [water including 0.1% (v/v) formic acid] and solvent B [acetonitrile including 0.1% (v/v) formic acid]; gradient program, 99.5%A/0.5%B at 0 min, 99.5%A/0.5%B at 0.1 min, 75%A/25%B at 10 min, 0.5%A/99.5%B at 10.1 min, 0.5%A/99.5%B at 12.0 min, 99.5%A/0.5%B at 12.1 min, and 99.5%A/0.5%B at 15.0 min; flow rate, 0.3 mL/min at 0 min, 0.3 mL/min at 10 min, 0.4 mL/min at 10.1 min, 0.4 mL/min at 14.4 min, and 0.3 mL/min at 14.5 min; column temperature, 40°C; MS detection: capillary voltage, +3.0 keV, cone voltage, 25.0 V, source temperature, 120°C, desolvation temperature, 450°C, cone gas flow, 50 L/h; desolvation gas flow, 800 L/h; collision energy, 6 V; mass range, *m/z* 50‒1,500; scan duration, 0.5 s; interscan delay, 0.014 s; data acquisition, centroid mode; polarity, positive; Lockspray (Leucine enkephalin): scan duration, 1.0 s; interscan delay, 0.1 s. MS/MS data were acquired in the ramp mode with the following analytical conditions: (1) MS: mass range, *m/z* 50–1,500; scan duration, 0.5 s; inter-scan delay, 0.014 s; data acquisition, centroid mode; polarity, positive and (2) MS/MS: mass range, *m/z* 50–1,500; scan duration, 0.1 s; inter-scan delay, 0.014 s; data acquisition, centroid mode; polarity, positive; collision energy, ramped from 10 to 50 V. In this mode, MS/MS spectra of the top 10 ions (>1,000 counts) in an MS scan were automatically obtained. If the ion intensity was less than 1,000, MS/MS data were not collected and the instrument moved to the next top 10 ions. Original peak intensity values were divided with those of the internal standard (lidocaine: *m/z *=* *235.181, retention time, 6.497 min) to normalize the peak intensity values and were shown as relative peak intensities.

#### Chemical synthesis of authentic standards for SC(4-*O*-8)G and FC(4-*O*-8)G

The authentic, racemic standards for neolignans SC(4-*O*-8)G (**1**) and FC(4-*O*-8)G (**4**) were synthesized as shown in Figure 1C. The starting compounds, i.e. ethyl sinapate **8SC** and ethyl ferulate **8FC** were synthesized according to methods described in the literature (Sakakibara et al., 2007), and 1-(4-acetoxy-3-methoxyphenyl)-1-ethanone was prepared from acetovanillone using standard acetylation procedures with pyridine and acetic anhydride. Other chemicals were purchased from Wako Pure Chemical (Osaka, Japan) or Nacalai Tesque (Kyoto, Japan) and were used as received. Flash chromatography was performed with Redi Sep Rf silica cartridges on a Combi Flash Rf system (Teledyne ISCO, Lincoln, NE, USA) using an ethyl acetate (EtOAc)/hexane gradient as the eluent and a UV detector (at 254 nm). Preparative HPLC was performed with an XBridge BEH C18 OBD Prep column (130 Å, 5 mm, 10 mm × 150 mm; Waters Co., Milford, USA) on a Shimadzu 20A HPLC system (Shimadzu Co. Ltd., Kyoto, Japan) using an acetonitrile/water gradient as the eluent and a UV detector (at 254 nm). NMR spectra were recorded on a JEOL JNM-LA400MT FT-NMR system (400 MHz, JEOL, Tokyo, Japan) or a Bruker Biospin AVANCE III 800US system (800 MHz, Bruker Biospin, Billerica, MA, USA). The central solvent peaks were used as internal references (δ_C_/δ_H_, acetone-*d*_6_, 29.84/2.04; chloroform-*d*, 77.00/7.26; methanol-*d*_4_, 49.00/3.31). Standard 1D and 2D (COSY, HSQC, and HMBC) NMR experiments were used for structural assignments. Determination of *erythro* and *threo* configurations was based on the comparison of NMR chemical shifts and coupling constant data reported for identical or analogous *erythro* and *threo* 8-*O*-4-type dimer models as reported by Ralph and Helm (1991) and Helm and Ralph (1992).

#### Compound 9

To a stirred solution of 1-(4-acetoxy-3-methoxyphenyl)-1-ethanone (9.6 mmol) in EtOAc (10 mL), pyridinium bromide perbromide (9.6 mmol) was added. After 2 h of stirring at room temperature, the reaction mixture was neutralized with 1 M NaHCO_3_ on ice and extracted three times with EtOAc over distilled water. The combined organic layer was washed with brine (a saturated aqueous solution of NaCl), dried over Na_2_SO_4_, and evaporated *in vacuo*. The crude product was then purified by flash chromatography to produce a white solid of compound **9** (48.9% yield). ^1^H-NMR (400 MHz, acetone-*d*_6_): *δ *= 7.74 (1H, d, *J* 8.8 Hz, H-6), 7.73 (1H, s, H-2), 7.27 (1H, d, *J* 8.8 Hz, H-5), 4.82 (2H, s, H-8), 3.95 (3H, s, OMe), 2.31 (3H, s, OAc).

#### Compound 10SC

To a suspension of compound **8SC** (3.48 mmol) and K_2_CO_3_ (3.48 mmol) in 10 mL acetone, compound **9** (3.87 mmol) in 20 mL acetone was added dropwise at room temperature and then refluxed for 16 h. After cooling to room temperature, the reaction mixture was filtered and added to EtOAc/hexane (1:2, v/v). The organic phase was washed with distilled water and brine, dried over Na_2_SO_4_, and evaporated *in vacuo* to a residue that was purified by flash chromatography to produce a colorless solid of compound **10SC** (57.4% yield). ^1^H-NMR (400 MHz, chloroform-*d*): *δ *= 7.70 (1H, s, H-2), 7.63 (1H, d, *J* 7.2 Hz, H-6) 7.60 (1H, d, *J* 15.6 Hz, H-7′), 7.13 (1H, d, *J* 8.0 Hz, H-5), 6.75 (2H, s, H-2′/6′), 6.35 (1H, d, *J* 15.8 Hz, H-8′), 5.24 (2H, s, H-8), 4.27 (2H, d, *J* 7.1 Hz, H-10′), 3.90 (3H, s, C-3OMe), 3.84 (6H, s, C-3′/5′OMe), 2.33 (3H, s, OAc), 1.34 (3H, t, *J* 7.1 Hz, H-11′).

#### Compound 10FC

Compound **10FC** was synthesized from compounds **8FC** and **9** using the above described procedure for compound **10SC** and was isolated as a colorless solid (73.3% yield). ^1^H-NMR (400 MHz, chloroform-*d*): *δ *= 7.66 (1H, s, H-2), 7.64-7.56 (2H, overlapped, H-6, H-7′), 7.16 (1H, d, *J* 7.8 Hz, H-5), 7.08 (1H, s, H-2′), 7.04 (1H, d, *J* 8.8 Hz, H-6′), 6.78 (1H, d, *J* 8.8 Hz, H-5′), 6.32 (1H, d, *J* 16.6 Hz, H-8′), 5.37 (1H, s, H-8), 4.26 (2H, q, *J* 7.1 Hz, H-10′), 3.93 (3H, s, C-3OMe), 3.90 (3H, s, C-3′OMe), 2.35 (3H, s, OAc), 1.34 (3H, t, *J* 7.1 Hz, H-11′).

#### Compound 11SC

To a stirred suspension of compound **10SC** (1.96 mmol) in 30 mL of dioxane, K_2_CO_3_ (14 mmol) and 37% (v/v) formaldehyde (3.92 mmol) were added. After 5 h of stirring at room temperature, the reaction mixture was filtered and concentrated *in vacuo* to a residue that was purified by flash chromatography to produce a colorless solid of compound **11SC** (76.2% yield). ^1^H-NMR (400 MHz, chloroform-*d*): *δ *= 7.72 (1H, s, H-2), 7.67 (1H, d, *J* 8.8 Hz, H-6), 7.58 (1H, d, *J* 15.6 Hz, H-7′), 7.12 (1H, d, *J* 8.0 Hz, H-5), 6.75 (2H, s, H-2′/6′), 6.35 (1H, d, *J* 15.6 Hz, H-8′), 5.15–5.16 (1H, m, H-8), 4.26 (2H, q, *J* 7.4 Hz, H-10′), 3.98–4.04 (2H, br m, H-9), 3.89 (3H, s, C-3OMe), 3.76 (6H, s, 3.76, C-3′/5′OMe), 2.34 (3H, s, OAc), 1.33 (3H, t, *J* 7.4 Hz, H-11′).

#### Compound 11FC

Compound **11FC** was synthesized from compound **10FC** using the above described procedure for compound **11SC** and was isolated as colorless solid (86.3% yield). ^1^H-NMR (400 MHz, chloroform-*d*): *δ *= 7.70 (1H, d, *J* 7.8 Hz, H-6), 7.68 (1H, s, H-2), 7.58 (1H, d, *J* 16.6 Hz, H-7′), 7.14 (1H, d, *J* 7.8 Hz, H-5), 7.06 (1H, s, H-2′), 7.00 (1H, d, *J* 8.8Hz, H-6′), 6.75 (1H, d, *J* 7.8 Hz, H-5′), 6.30 (1H, d, *J* 16.6 Hz, H-8′), 5.52 (1H, d, *J* 5.9Hz, H-8), 4.25 (2H, q, *J* 7.1 Hz, H-10′), 4.08–4.16 (2H, m, H-9), 3.87 (3H, s, C-3OMe), 3.86 (3H, s, C-3′OMe), 2.34 (3H, s, OAc), 1.33 (3H, t, *J* 7.1 Hz, H-11′).

#### Compound 12SC

To a solution of compound **12SC** (1.45 mmol) in 25 mL ethanol, NaBH_4_ (10.5 mmol) was added at room temperature. The reaction mixture was stirred under nitrogen gas at room temperature for 5 min, and then the excessive NaBH_4_ was quenched by adding acetic acid (1 mL). The reaction mixture was concentrated *in vacuo* and extracted three times with EtOAc (10 mL) over water (10 mL). The combined organic layer was washed with brine, dried over Na_2_SO_4_, and evaporated *in vacuo* to a crude oil that was purified by flash chromatography to produce a colorless solid of compound **12SC** as a mixture of *erythro* and *threo* isomers (95.8% yield, *erythro*/*threo* isomer ratio = 7:3 as determined by ^1^H-NMR). **12SC** (*erythro* isomer): ^1^H-NMR (400 MHz, acetone-*d*_6_): *δ*  =  7.65 (1H, d, *J* 15.6 Hz, H-7′), 7.25 (1H, s, H-2), 7.13 (2H, s, H-2′/6′), 7.08–7.11 (1H, br d, H-6), 7.01–7.04 (1H, br d, H-5), 6.53 (1H, d, *J* 15.6 Hz, H-8′), 5.12 (1H, br m, H-7), 4.29–4.34 (1H, m, H-8), 4.25 (2H, q, *J* 6.8 Hz, H-10′), 3.96 (6H, s, C-3′/5′OMe), 3.87 (3H, s, C-3OMe), 3.76–3.82 (1H, m, H-9A), 3.49–3.58 (1H, m, H-9B), 2.27 (3H, s, OAc), 1.33 (3H, t, *J* 6.8 Hz, H-11′). **12SC** (*threo* isomer): ^1^H-NMR *δ*_H_ (acetone-*d*_6_) *δ*  =  7.65 (1H, d, *J* 15.6 Hz, H-7′), 7.27 (1H, s, H-2), 7.13 (2H, s, H-2′/6′), 7.08–7.11 (1H, br, H-6), 7.01–7.04 (1H, br, H-5), 6.58 (1H, d, *J* 15.6 Hz, H-8′), 5.16 (1H, br m, H-7), 4.25 (2H, q, *J* 6.8 Hz, H-10′), 4.16–4.20 (1H, m, H-8), 3.99 (6H, s, C-3′/5′OMe), 3.84 (3H, s, C-3OMe), 3.61–3.70 (1H, m, H-9A), 3.40–3.48 (1H, m, H-9B), 2.27 (3H, s, OAc), 1.33 (3H, t, *J* 6.8 Hz, H-11′).

#### Compound 12FC

Compound **12FC** was synthesized from **11FC** using the above described procedure for compound **12SC** and was isolated as a mixture of *erythro* and *threo* isomers in the form of a colorless solid (86.3% yield, *erythro*/*threo* isomer ratio = 6:4 as determined by ^1^H-NMR). **12FC** (*erythro* isomer): ^1^H-NMR (400 MHz, acetone-*d*_6_): *δ*  =  7.65 (1H, d, *J* 15.6 Hz, H-7′), 7.37 (1H, s, H-2′), 7.33 (1H, s, H-2), 7.07–7.25 (overlapped, 4H, H-5, H-6, H-5′, and H-6′), 6.47 (1H, d, *J* 16.4 Hz, H-8′), 5.07 (1H, m, H-7), 4.49–4.53 (2H, br, H-8), 4.21–4.29 (2H, q, *J* 6.8 Hz, H-10′), 3.98 (3H, s, C-3′OMe), 3.94 (3H, s, C-3OMe), 3.82–3.90 (1H, m, H-9A), 3.61–3.65 (1H, m, H-9B), 2.27 (3H, s, OAc), 1.33 (3H, t, *J* 6.8 Hz, H-11′). **12FC** (*threo* isomer): ^1^H-NMR (400 MHz, acetone-*d*_6_): *δ*  =  7.63 (1H, d, *J* 15.6 Hz, H-7′), 7.41 (1H, s, H-2′), 7.33 (1H, s, H-2), 7.07–7.25 (overlapped, 4H, H-5, H-6, H-5′, and H-6′), 6.49 (1H, d, *J* 16.4 Hz, H-8′), 5.07 (1H, m, H-7), 4.54–4.58 (2H, m, H-8), 4.21–4.29 (2H, q, *J* 6.4 Hz, H-10′), 3.98 (3H, s, C-3′OMe), 3.94 (3H, s, C-3OMe), 3.82–3.90 (1H, m, H-9A), 3.61–3.65 (1H, m, H-9B), 2.27 (3H, s, OAc), 1.33 (3H, t, *J* 6.4 Hz, H-11′).

#### Compound 13SC

Compound **12SC** (1.12 mmol) was dissolved in 20 mL of 2 M NaOH. After stirring for 10 min at room temperature, the reaction mixture was acidified with 3 M HCl, and extracted three times with CH_2_Cl_2_-EtOAc (1:1, v/v). The combined organic layer was washed with brine, dried over Na_2_SO_4_, and evaporated *in vacuo*. The crude product was purified by flash chromatography to give a colorless oil of compound **13SC** as a mixture of *erythro* and *threo* isomers (48.2% yield, *erythro*/*threo* isomer ratio = 7:3 as determined by ^1^H-NMR). **13SC** (*erythro* isomer): ^1^H-NMR (400 MHz, methanol-*d*_4_): *δ*  =  7.63 (1H, d, *J* 15.6, H-7′), 7.02 (1H, s, H-2), 6.96 (2H, s, H-2′/6′), 6.83 (1H, d, *J* 8.7 Hz, H-6), 6.78 (1H, d, *J* 8.7 Hz, H-5), 6.47 (1H, d, *J* 15.6 Hz, H-8′), 5.04 (1H, br d, H-7), 4.36–4.39 (1H, m, H-8), 3.95–3.98 (1H, m, H-9A), 3.90 (3H, s, C-3OMe), 3.86 (6H, s, C-3′/5′OMe), 3.63–3.67 (1H, m, H-9B). **13SC** (*threo* isomer): ^1^H-NMR (400 MHz, methanol-*d*_4_): *δ*  =  7.64 (1H, d, *J* 15.6 Hz, H-7′), 7.06 (1H, s, H-2), 7.02 (2H, s, H-2′/6′), 6.91 (1H, d, *J* 8.8 Hz, H-6), 6.79 (1H, d, *J* 8.8 Hz, H-5), 6.49 (1H, d, *J* 15.6 Hz, H-8′), 5.04 (1H, br d, H-7), 4.18–4.22 (1H, m, H-8), 3.82–3.85 (1H, m, H-9A), 3.93 (3H, s, C-3OMe), 3.87 (6H, s, C-3′/5′OMe), 3.40–3.42 (1H, m, H-9B).

#### Compound 13FC

Compound **13FC** was synthesized from **12FC** using the above described procedure for compound **7SC** and was isolated as a mixture of *erythro* and *threo* isomers in the form of a colorless oil (97.0% yield, *erythro*/*threo* isomer ratio = 6:4 as determined by ^1^H-NMR). **13FC** (*erythro* isomer): ^1^H-NMR (400 MHz, methanol-*d*_4_): *δ*  =  7.62 (1H, d, *J* 15.6 Hz, H-7′), 7.36 (1H, s, H-2′), 7.21 (1H, d, *J* 8.8 Hz, H-6′), 7.12–7.14 (overlapped, 2H, H-2, and H-5′), 6.92 (1H, d, *J* 8.8 Hz, H-6), 6.78 (1H, d, *J* 8.8 Hz, H-5), 6.44 (1H, d, *J* 15.6 Hz, H-8′), 4.91–4.92 (1H, m, H-7), 4.37–4.41 (1H, m, H-8), 3.95 (3H, s, C-3′OMe), 3.83 (3H, s, C-3OMe), 3.72–3.77 (1H, m, H-9A), 3.53–3.58 (1H, m, H-9B). **13FC** (*threo* isomer): ^1^H-NMR (400 MHz, methanol-*d*_4_): *δ*  =  7.58 (1H, d, *J* 15.6 Hz, H-7′), 7.31 (1H, s, H-2′), 7.19 (1H, d, *J* 8.8 Hz, H-6′), 7.12–7.14 (1H, br s, H-2), 7.05 (1H, d, *J* 8.8 Hz, H-5’), 6.92 (1H, d, *J* 8.8 Hz, H-6), 6.76 (1H, br d, H-5), 6.42 (1H, d, *J* 15.6 Hz, H-8′), 4.91–4.92 (1H, m, H-7), 4.46–4.49 (1H, m, H-8), 3.95 (3H, s, C-3′OMe), 3.83 (3H, s, C-3OMe), 3.72–3.77 (1H, m, H-9A), 3.53–3.58 (1H, m, H-9B).

#### 3-{4-[2-Hydroxy-2-(4-hydroxy-3-methoxyphenyl)-1-hydroxymethylethoxy]-3′,5′-dimethoxyphenyl}acryloylcholine [SC(4-*O*-8)G (1)]

Choline esterification of compound **13SC** was carried out according to the method of Böttcher et al. (2008). Briefly, to a solution of compound **13SC** (0.116 mmol) in DMF (220 µL), 1 M NaOH (110 µL), and 1 M bromocholine bromide in water (220 µL) were successively added at room temperature. The reaction mixture was then heated to 90°C for 24 h. After cooling to room temperature, the reaction mixture was diluted with water (2 mL), eluted on a Strata-X-CW solid-phase extraction cartridge (Phenomenex, Torrance, USA), and concentrated *in vacuo*, to give a crude mixture of *erythro* and *threo* isomers **1***e* and **1***t* as a colorless oil (yield 65.6%). A portion of this crude mixture was dissolved in H_2_O-MeOH (9:1, v/v), and subjected to preparative HPLC to isolate pure compounds **1***e* and **1***t*. *erythro*-SC(4-*O*-8)G (**1***e*): ^1^H-NMR (800 MHz, methanol-*d*_4_): *δ*  =  7.70 (1H, d, *J* 15.9 Hz, H-7′), 6.99 (1H, d, *J* 1.9 Hz H-2), 6.95 (2H, s, H-2′/6′), 6.79 (1H, dd, *J* 8.2, 1.9 Hz, H-6), 6.73 (1H, d, *J* 8.2 Hz, H-5), 6.55 (1H, d, *J* 15.9 Hz, H-8′), 4.90 (1H, d, *J* 5.2 Hz, H-7), 4.69-4.65 (2H, m, H-10′), 4.37–4.34 (1H, m, H-8), 3.91 (1H, dd, *J* 12.0, 5.4 Hz, H-9A), 3.85 (6H, s, C-3′/5′OMe), 3.83 (3H, s, C-3OMe), 3.79–3.77 (2H, m, H-11′), 3.62 (1H, br dd, H-9B), 3.26 (9H, s, H-12′NMe); ^13^C-NMR (200 MHz, methanol-*d*_4_): *δ*  =  167.47 (C-9), 154.80 (C-3′/5′), 148.68 (C-3), 147.53 (C-7′), 146.96 (C-4), 139.57 (C-4′), 133.85 (C-1), 131.27 (C-1′), 120.80 (C-6), 117.11 (C-8′), 115.68 (C-5), 111.58 (C-2), 107.01 (C-2′/6′), 87. 52 (C-8), 74.18 (C-7), 66.25 (C-11′), 61.80 (C-9), 58.89 (C-10′), 56.80 (C-3′/5′OMe), 56.37 (C-3OMe), 54.48 (C-12′NMe). *threo*-SC(4-*O*-8)G (**1***t*): ^1^H-NMR (800 MHz, methanol-*d*_4_): *δ*  =  7.71 (1H, d, *J* 15.9 Hz, H-7′), 7.01 (1H, d, *J* 1.9 Hz, H-2), 6.98 (2H, s, H-2′/6′), 6.87 (1H, dd, *J* 8.4, 1.9 Hz, H-6), 6.75 (1H, d, *J* 8.2 Hz, H-5), 6.57 (1H, d, *J* 15.9 Hz, H-8′), 4.99 (1H, d, *J* 6.6 Hz, H-7), 4.69–4.65 (2H, m, H-10′), 4.22–4.19 (1H, m, H-8), 3.99 (6H, s, C-3′/5′OMe), 3.83 (3H, s, C-3OMe), 3.80–3.76 (3H, overlapped, H-9A and H-11′), 3.37 (1H, dd, *J* 11.9, 3.5 Hz, H-9B), 3.26 (9H, s, H-12′NMe); ^13^C-NMR (200 MHz, methanol-*d*_4_): *δ*  =  167.49 (C-9), 154.60 (C-3′/5′), 148.75 (C-3), 147.21 (C-7′), 147.21 (C-4), 139.96 (C-4′), 133.54 (C-1), 131.43 (C-1′), 120.83 (C-6), 117.30 (C-8′), 115.84 (C-5), 111.77 (C-2), 106.99 (C-2′/6′), 88. 88 (C-8), 74.41 (C-7), 66.24 (C-11′), 61.92 (C-9), 58.92 (C-10′), 56.83 (C-3′/5′OMe), 56.38 (C-3OMe), 54.51 (C-12′NMe).

#### 3-{4-[2-Hydroxy-2-(4-hydroxy-3-methoxyphenyl)-1-hydroxymethylethoxy]-3′-methoxyphenyl}acryloylcholine [FC(4-*O*-8)G (4)]

Compound **4** as a crude mixture of *erythro* and *threo* isomers **4***e* and **4***t* (colorless oil, 46.3% yield) was prepared from **13FC** using the above described procedure for compound **1.** Pure isomers **4***e* and **4***t* were then isolated by preparative HPLC. *erythro*-FC(4-*O*-8)G (**4***e*): ^1^H-NMR (800 MHz, methanol-*d*_4_): *δ*  =  7.66 (1H, d, *J* 15.9 Hz, H-7′), 7.18 (1H, d, *J* 2.0 Hz, H-2′), 7.10 (1H, dd, *J* 8.4, 2.0 Hz, H-6′), 7.03 (1H, d, *J* 2.0 Hz, H-2), 6.97 (1H, dd, *J* 8.2, 2.0 Hz, H-5′), 6.84 (1H, dd, *J* 8.2, 2.0 Hz, H-6), 6.70 (1H, d, *J* 8.2 Hz, H-5), 6.45 (1H, d, *J* 15.9, H-8′), 4.81 (1H, d, *J* 6.0 Hz, H-7), 4.67–4.64 (2H, m, H-10′), 4.51 (1H, m, H-8), 3.84 (1H, br, H-9A), 3.83 (1H, br, H-9B), 3.82 (3H, s, C-3′OMe), 3.80 (3H, s, C-3OMe), 3.79–3.76 (2H, m, H-11′), 3.25 (9H, s, H-12′NMe); ^13^C-NMR (200 MHz, methanol-*d*_4_): *δ*  =  167.70 (C9′), 152.24 (C-4′), 151.82 (C-3′), 148.67 (C-3), 147.58 (C-7′), 147.10 (C-4), 133.99 (C-1), 129.14 (C-1′), 123.81 (C-6′), 121.17 (C-6), 117.40 (C-5′), 115.61 (C-5), 115.46 (C-8′), 112.54 (C-2′), 112.06 (C-2), 85.47 (C-8), 74.11 (C-7), 66.27 (C-11′), 62.48 (C-9), 58.78 (C-10′), 56.63 (C-3′OMe), 56.34 (C-3OMe), 54.47 (C-12′NMe). *threo*-FC(4-*O*-8)G (**4***t*): ^1^H-NMR (800 MHz, methanol-*d*_4_): *δ*  =  7.69 (1H, d, *J* 15.9 Hz, H-7′), 7.25 (1H, d, *J* 2.0 Hz, H-2′), 7.14 (1H, dd, *J* 8.4, 2.0 Hz, H-6′), 7.06 (1H, d, *J* 8.4 Hz, H-5′), 7.03 (1H, d, *J* 2.0 Hz, H-2), 6.85 (1H, dd, *J* 8.1, 2.0 Hz, H-6), 6.75 (1H, d, *J* 8.1 Hz, H-5), 6.47 (1H, d, *J* 15.9 Hz, H-8′), 4.89 (1H, d, *J* 5.4 Hz, H-7), 4.68–4.63 (2H, m, H-10′), 4.48–4.45 (1H, m, H-8), 3.90 (3H, s, C-3′OMe), 3.82 (3H, s, C-3OMe), 3.79–3.75 (3H, overlapped, H-9A and H-11′), 3.53 (1H, dd, *J* 11.9, 5.8 Hz, H-9B), 3.25 (9H, s, H-12′NMe); ^13^C-NMR (200 MHz, methanol-*d*_4_): *δ*  =  167.69 (C9′), 152.49 (C-4′), 151.74 (C-3′), 148.85 (C-3), 147.53 (C-7′), 147.22 (C-4), 133.81 (C-1), 129.27 (C-1′), 123.96 (C-6′), 120.66 (C-6), 117.40 (C-5′), 115.85 (C-5), 115.60 (C-8′), 112.51 (C-2′), 111.74 (C-2), 86. 21 (C-8), 73.91 (C-7), 66.27 (C-11′), 62.09 (C-9), 58.80 (C-10′), 56.68 (C-3′OMe), 56.35 (C-3OMe), 54.48 (C-12′NMe).

#### Phylogenetic analysis

Deduced amino acid sequences of DIRs or LACs were aligned using the CLUSTAL W program in MEGA X (version 10.1, https://www.megasoftware.net/; Kumar et al., 2018). A phylogenetic tree was constructed using the Neighbor-Joining method (Saitou and Nei, 1987) in MEGA X with the following parameters: bootstrap test (500 replicates), Poisson model, uniform rates, and complete deletion. Machine-readable files of the phylogenetic analyses used to for Figures 6 and 9 are provided as Supplemental Files 1 and 2, respectively.

#### Reverse transcription quantitative PCR

RNA extraction, cDNA synthesis, and reverse transcription quantitative PCR (RT-qPCR) were performed as described previously (Yonekura-Sakakibara et al., 2014). The developmental stages of leaves, stems, roots, floral buds, and flowers used for analyses were as described previously (Yonekura-Sakakibara et al., 2007). The developmental stages of siliques were as follows: stage 3, 24–36 h after flowering (HAF); stage 4, 36–48 HAF; stage 5, 48–72 HAF; stage 6, 72–96 HAF; stage 7, 96–108 HAF; stage 8, 108–120 HAF; stage 9, 120–132 HAF; stage 10, 132–144 HAF; stage 11, 144–192 HAF. Primers used are described in Supplemental Table 2 (At4g11180cDNA-RT101f and At4g11180cDNA-RT186r for *AtDP1/AtDIR12*, At1g61720pda14333_RT107f andAt1g61720pda14333_RT179r for *BANYULS*, At5g48100pda12625_RT478f and At5g48100pda12625_RT552r for *AtLAC15/TT10*, and At2g40370qPCRNo2_1090F and At2g40370qPCRNo2_1107R for *AtLAC5*) and were checked for specific product formation using a dissociation program. Plasmid DNAs containing the corresponding genes were used as templates for calibration. RT-qPCR was performed in triplicate.

#### Generation and analysis of YFP/GUS reporter lines

A 720-bp fragment of the *AtDP1/AtDIR12* promoter region or the promoter region including the *AtDP1/AtDIR12* coding region was amplified by PCR using primers, At4g11180_CCAC+promoter F and At4g11180_promoter R or At4g11180_R (Supplemental Table 2). Amplified fragments were cloned into the pENTR/D-TOPO vector (Invitrogen) as the entry vector. The resulting plasmid was sequenced to confirm the absence of PCR errors. pHGY, pH35GY derivatives from which the CaMV 35S promoter sequence is deleted (Endo et al., 2009) and pBGGUS (Funakoshi, Japan) were used as the destination vectors. The LR reactions for the binary vectors pKYS405 (pHGY/ProAtDP1-AtDP1CDS-YFP) and pKYS415 (pBGGUS/ProAtDP1-GUS) were catalyzed using the Gateway LR Clonase^TM^II enzyme mix (Invitrogen).

The 4,140-bp or 2,000-bp fragments of the *AtLAC5* promoter region were amplified by PCR using primers, At2g40370pro_CCAC + 4140F or At2g40370pro_CCAC + 2000F and At2g40370ATG_R (Supplemental Table 2). Amplified fragments were cloned into the pENTR/D-TOPO vector (Invitrogen) as the entry vector. The binary vectors, pKYS540 (pBGGUS/ProAtLAC5-4140-GUS) and pKYS541 (pBGGUS/ProAtLAC5-2000-GUS), were constructed using pBGGUS (Funakoshi, Japan) as the destination vector as described above.pKYS405 (pHGY/ProAtDP1-AtDP1CDS-YFP), pKYS415 (pBGGUS/ProAtDP1-GUS), pKYS540 (pBGGUS/ProAtLAC5-4140-GUS), and pKYS541 (pBGGUS/ProAtLAC5-2000-GUS) were transformed into *Agrobacterium* and subsequently into Arabidopsis plants as described above. Transgenic T2 plants for pKYS405 were selected on half-strength Murashige and Skoog medium containing 25 mg L^−1^ hygromycin B and 50 mg L^−1^ carbenicillin disodium salt and those for pKYS415, pKYS540, and pKYS541 were selected on Murashige and Skoog medium containing 50 μM glufosinate ammonium and 50 mg L^−1^ carbenicillin disodium salt.

#### Confocal laser scanning microscopy, light microscopy, and electron microscopy

Fluorescent samples were observed using a confocal laser scanning microscope system (Model LSM510 META, Axioplan2 imaging, a Plan-Apochromat lens (63X/1.4 oil DIC; optical slice, 1 μm); Carl Zeiss) with 488-nm excitation and a 505–530 nm band-pass filter for YFP, 488-nm excitation and a 650-nm long-pass filter for chlorophyll autofluorescence and a 25-mV argon laser. Composite figures were prepared using the Zeiss LSM Image Browser software.

For histochemical GUS assays, tissues were treated with ice-cold 90% (v/v) acetone for 20 min on ice and stained in 100 mM sodium phosphate buffer, pH 7.0, 10 mM EDTA, 0.1% (v/v) Triton X-100, 0.5 mg mL^−1^ 5-bromo-4-chloro-3-indolyl glucuronide, 2 mM potassium ferricyanide, and 2 mM potassium ferrocyanide. After 60 min vacuum infiltration, samples were incubated overnight at 37°C and subsequently de-stained by a series of washes in 70% (v/v) ethanol, ethanol/acetic acid (6:1 v/v ratio), 70% (v/v) ethanol. For sectioning, tissue samples were fixed with 2% (v/v) glutaraldehyde and 1% (v/v) formaldehyde in 0.1 M phosphate buffer, pH 7.0, for 24 h at 4°C, washed in phosphate buffer, dehydrated using a series of graded ethanol solutions [30%, 40%, 60%, 70%, 85%, 95%, 100% (v/v)] and embedded in Technovit 7100 resin (Heraeus Kulzer, Wehrheim, Germany) according to the manufacturer’s instructions. Tissue sections were sliced into 5-µm transverse sections using a rotary microtome (Leica RM2125) and tungsten carbide disposable blades (Leica TC-65). Sections were observed using an Olympus BX53 microscope.

Surfaces of dry seeds were observed using a scanning electron microscope (Hitachi TM-1000).

#### Coexpression analyses

Coexpression analyses were conducted as described previously (Yonekura-Sakakibara et al., 2007, 2008; Saito et al., 2008). The genes encoding LACs (*r *>* *0.3) were extracted from the list of genes co-expressed with *AtDP1/AtDIR12*.

#### Seed coat permeability test

Seed coat permeability was tested by a tetrazolium penetration assay (Vishwanath et al., 2013, 2014). Dried seeds (10 mg) were incubated in 250 μL of an aqueous solution with or without 1% (w/v) tetrazolium red (2,3,5-triphenyltetrazolium chloride, Wako, Japan) at 30°C for 48 h in the dark. After incubation, the samples were washed with water, resuspended in 1 mL 95% (v/v) ethanol, and finely ground with a mortar and pestle to extract the formazans. After centrifugation at 15,000*g* for 3 min, the supernatant fraction was recovered and the absorbance at 485 nm was measured. Three biological replicates were used for the analyses.

#### Proanthocyanidin analysis

PA extraction and acid hydrolysis were performed in triplicate as described previously (Tohge et al., 2005; Kitamura et al., 2010). Mature seeds (10 mg) were homogenized in 0.75 mL of 70% acetone containing 5.26 mM Na_2_S_2_O_5_ in a mixer mill (Qiagen Retsch MM300 TissueLyser) for 1 min at 20 Hz, followed by sonication for 20 min. After centrifugation at 15,000*g* for 5 min, the supernatant fraction was evaporated and resuspended in 1 mL of HCl: butanol: 70% acetone (2:10:3). The absorbance of the solutions before/after hydrolysis at 95°C for 60 min was measured at 545 nm and the difference was treated as the soluble PA fraction. The pellet after extraction with 70% acetone was also evaporated, suspended in the HCl: butanol: 70% acetone solution and hydrolyzed as the insoluble PA fraction.

#### Lignin analysis

Mature seeds (40 mg) were pulverized, extracted successively with chloroform–methanol (2:1, v/v), 100% methanol, and water, followed by freeze-drying to obtain the seed cell-wall fractions (Tobimatsu et al., 2013). The seed cell-wall fractions were then subjected to analytical thioacidolysis (Lapierre et al., 1986) using the modified method described by Yamamura et al., 2012. The released lignin monomers were derivatized with *N*,*O*-bis(trimethylsilyl)acetamide and quantified by gas chromatography/mass spectrometry using 4,4′-ethylenebisphenol as an internal standard (Yue et al., 2012).

#### Accession numbers

Sequence data from this article can be found in the Arabidopsis Genome Initiative or GenBank/EMBL libraries under the following accession numbers: AtDP1/AtDIR12 (At4g11180, BT004016), AtDIR5 (At1g64160, BT010486), AtDIR6 (At4g23690, BT002439), AtDIR13 (At4g11190, BT015628), AtDIR14 (At4g11210, BT015626), FiDIR1 (AF210061), LuDIR1 (KM433751), LuDIR5 (KM433753), LuDIR6 (KM433752), PsDRR206 (U11716), ScDIR (HQ428029), TpDIR5 (AF210067), TpDIR6 (AF210070), AtLAC1 (At1g18140, NP_173252); AtLAC2 (At2g29130, NP_180477); AtLAC3 (At2g30210, NP_180580); AtLAC4 (At2g38380, NP_565881); AtLAC5 (At2g40370, NP_181568); AtLAC6 (At46570, NP_182180); AtLAC7 (At3g09220, NP_187533); AtLAC8 (At5g01040, NP_195724); AtLAC9 (At5g01050, NP_195725); AtLAC10 (At5g01190, NP_195739); AtLAC11 (At5g03260, NP_195946); AtLAC12 (At5g05390, NP_196158); AtLAC13 (At5g07130, NP_196330); AtLAC14 (At5g09360, NP_196498); AtLAC15 (At5g48100, NP_199621); AtLAC16 (At5g58910, NP_200699); AtLAC17 (At5g60020, NP_200810); BdLAC5 (Bradi1g66720, XP_003558240); BnTT10 (NP_001302959); GaLAC1 (NP_001316972); PtLAC3 (CAC14719); SofLAC (SCVPRZ3027A08.g and SCUTST3084C11.g); ZmLAC3 (CAJ30499).

### Supplemental data

The following materials are available in the online version of this article.

**Supplemental Figure 1**. Identification of neolignan FC(4-*O*-8)G (**4**) in Arabidopsis seeds.

**Supplemental Figure 2.** Multiple alignment of DIRs in the DIR-a family.

**Supplemental Figure 3**. Multiple alignment of Arabidopsis LACs and functionally identified LACs.

**Supplemental Figure 4**. T-DNA insertion mutants of *AtLAC5*.

**Supplemental Figure 5**. Expression pattern of *AtLAC5* and *AtDP1/AtDIR12* using the Arabidopsis eFP browser (Winter et al., 2007) and Arabidopsis Seed Coat eFP browser (Dean et al., 2011).

**Supplemental Figure 6**. A proposed model for gene duplication of the Arabidopsis DIR-a gene family.

**Supplemental Table 1.** Metabolites detected in seeds of wild-type and the tested mutants.

**Supplemental Table 2.** Primers used in this study.

**Supplemental Data Set 1.**  ^1^H and ^13^C NMR spectra of synthetic neolignans SC(4-*O*-8)G and FC(4-*O*-8)G.

**Supplemental Data Set 2.** One-way analysis of variance for Figure 5.

**Supplemental Data Set 3.** One-way analysis of variance for Figure 12.

**Supplemental Data Set 4.** One-way analysis of variance for Figure 13.

**Supplemental File 1.** Alignment corresponding to the phylogenetic analysis in Figure 6.

**Supplemental File 2.** Alignment corresponding to the phylogenetic analysis in Figure 9.

## Supplementary Material

koaa014_Supplementary_Data

## Notes

These authors contributed equally to this work (K.Y.-S., M.Y.).

K.Y.-S., T.U., and K.S. designed research. K.Y.-S., M.Y., F.M., E.O., R.N., S.S., T.M., and Y.T. performed research. K.Y.-S., M.Y., F.M., R.N., T.M., and Y.T. analyzed the data. K.Y.-S., M.Y., Y.T., T.U., and K.S. wrote the article.

The authors responsible for distribution of materials integral to the findings presented in this article in accordance with the policy described in the Instruction for Authors (www.plantcell.org) are: Kazuki Saito (kazuki.saito@riken.jp), and Toshiaki Umezawa (tumezawa@rish.kyoto-u.ac.jp).
