# Supplementary material for: Seed-coat protective neolignans are produced by the dirigent protein AtDP1 and the laccase AtLAC5 in Arabidopsis
Source: Plant Cell. 2020 Nov 27;33(1):129–52. doi: 10.1093/plcell/koaa014 (PMC8136895; doi:10.1093/plcell/koaa014)
Supplement: koaa014_Supplementary_Data [file koaa014_supplementary_data.zip › tpc.00658.2020-s04.pdf]

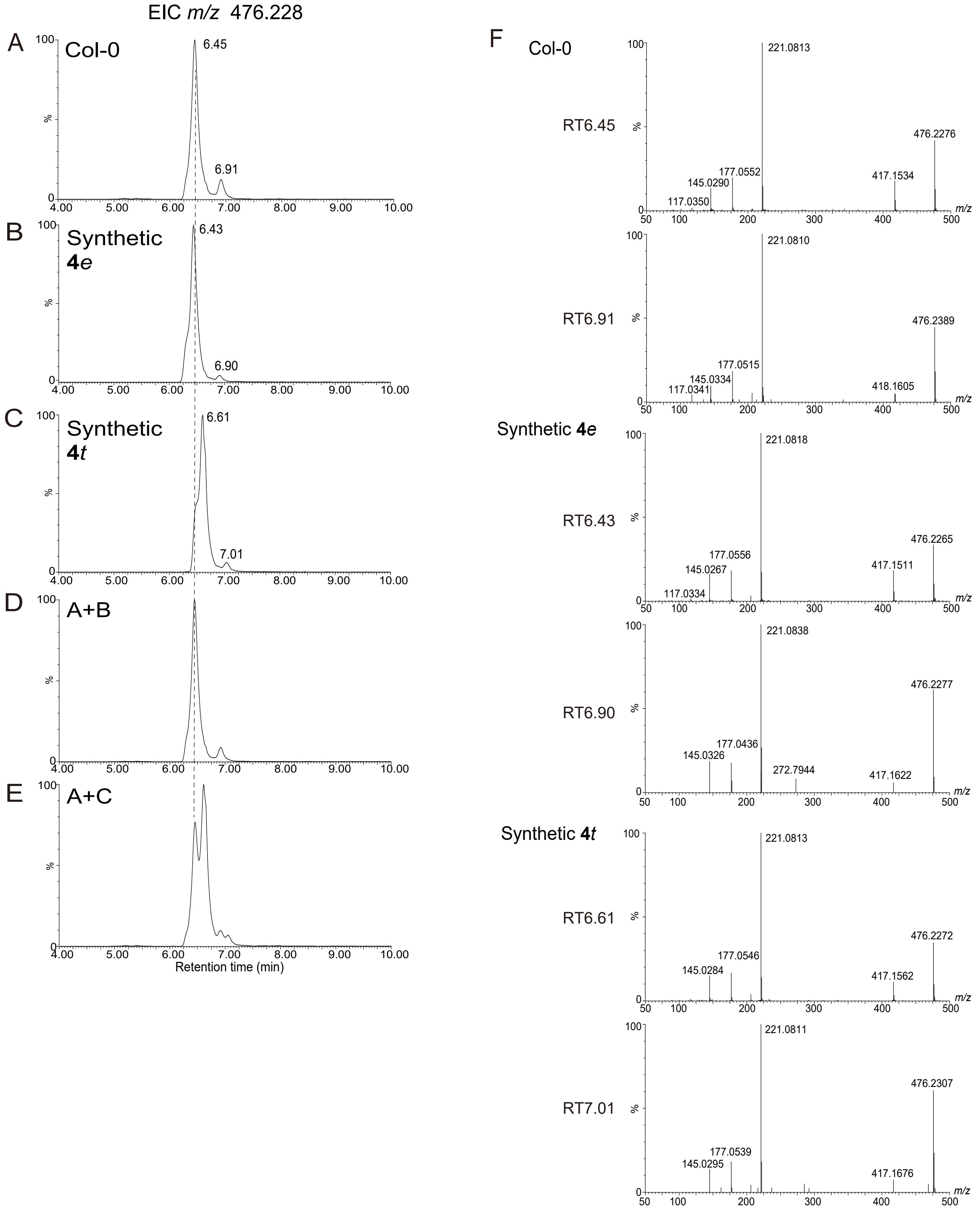


Supplemental Figure 1. Identification of neolignan FC(4-*O*-8)G (**4**) in Arabidopsis seeds.

(A-C) Extracted ion chromatogram (EIC, 476.228 m/z) of synthetic standards of *erythro*-FC(4-*O*-8)G (**4***e*) (B) and *threo*-FC(4-*O*-8)G (**4***t*) (C) and aqueous methanol extracts from the Arabidopsis Col-0 seeds (A). (D) A co-eluted with B. (E) A co-eluted with C. (F) MS/MS spectra of synthetic **4***e* and **4***t* standards and the corresponding product from Col-0. The structural interpretation of fragment ions was previously reported (Böttcher et al., 2008). RT, retention time. Supporting information for Figs. 4 and 5.

```
DP1_AtDIR12       M--------- -TNQIYKQVF SFFLSVLLLQ SSTVSYVP-- --KSFDL--- ----KKPCKH FVLYLHNIAY DG-DNAANAT  58
AtDIR13           M--------- -ANQIYIISL -IFLSVLLYQ STTVLSFR-- --QPFNL--- ----AKPCKR FVFYLHNVAY DG-DNTDNAT  57
AtDIR14           M--------- -ANQIYLFSL -ICLSVLLCQ SYTVSSFQ-- --KSLDL--- ----AKPCKR FVLHLHDIAY DG-DNAANAT  57
AtDIR5            M--------- ----VGQMKS FLFLFVFLVL TKTVISAR-- --KPSKS--- --QP-KPCKN FVLYYHDIMF -GVDDVQNAT  56
AtDIR6            M------AFL VEKQLFKALF SFFLLVLLFS -DTVLSFR-- --KTIDQ--- ----KKPCKH FSFYFHDILY DG-DNVANAT  61
LuDIR5            MKHSSSHSSS CLPFLLTTTT PIFLLLLSLI CPAAATWR-- --TPTHH-QH GRNPNKPCKQ LVLYYHDILF HGNGDQGNAT  75
LuDIR6            MKHTSS---- -FHFLLTTTT LIFLLLLSLI SPGDATWR-- --TPSHHLQH AKHP-KPCKQ LVLYYHDILF HGNGDQGNAT  70
ScDIR             M--------E GRKLIITIPL LLFFIAFFSV PPAAFGRKVT LPRKRMP--- -----QPCMN LVFYFHDILY NG-KNAANAT  63
FiDIR             M--------- ---VSKTQIV ALFLCFLTST SSATYGRK-- -PRPRR---- ------PCKE LVFYFHDVLF KG-NNYHNAT  54
PsDRR206          M--------- -----GSKLL VLFVFVMLFA LSSAIP---- -NKRKPY--- -----KPCKN LVFYFHDILY NG-KNAANAT  52
LuDIR1            M--------- ----AISRSN IALFFIFFIC LSSANSS--- -AKKKQH--- -----TPCKE LVLFFHDIIY NG-HNKANAT  54
TpDIR5            M--------- -KAIRVLHLC FLCLLVSAIL LKSADCHS-- -WKKKLP--- -----KPCKN LVLYFHDIIY NG-KNAENAT  58
TpDIR8            M--------A IWNGRVLNLC ILWLLVSIVL LNGIDCHS-- -RKKKLP--- -----KPCRN LVLYFHDIIY NG-KNAGNAT  60

DP1_AtDIR12       AATIVKPLG- -----LGDHS FGELIIINNP VTLDQNYLSK PVARAQGFYF YNMKTNYNAW VAWTLVFNST KHKGTFTIMD 132
AtDIR13           SAAIVNPLG- -----LGDFS FGKFVIMDNP VTMDQNMLSE QVARVQGFFF YHGKTKYDTW LSWSVVFNST QHKGALNIMG 131
AtDIR14           SAAIVNPLG- -----LGDFS FGKFVIMDDP VTMDQNYLSK PVARVQGFFC YHGKATYDAW IAWTVVFNST QHKGAFTIMG 131
AtDIR5            SAAVTNPPG- -----LGNFK FGKLVIFDDP MTIDKNFQSE PVARAQGFYF YDMKNDYNAW FAYTLVFNST QHKGTLNIMG 130
AtDIR6            SAAIVSPPG- -----LGNFK FGKFVIFDGP ITMDKNYLSK PVARAQGFYF YDMKMDFNSW FSYTLVFNST EHKGTLNIMG 135
LuDIR5            SAAAANATK- -----LGDYK FGMLVVFDDP VTKDGHLKSK AVARAQGFYF YDMKSTYNAW FAYTLVFNST EHKGTINIMG 149
LuDIR6            SAAAANATK- -----LGDYK FGMLVVFDDP VTKDGHLKSK AVARAQGFYF YDMKSTYNAW FAYTLVFNST DHKGTLNIMG 144
ScDIR             SAIVGSPAWG NRTILAGQSN FGDMVVFDDP ITLDNNLHSP PVGRAQGFYF YDRKDVFTAW LGFSFVFNNS DYRGSINFAG 143
FiDIR             SAIVGSPQWG NKTAMAVPFN YGDLVVFDDP ITLDNNLHSP PVGRAQGMYF YDQKNTYNAW LGFSFLFNST KYVGTLNFAG 134
PsDRR206          SAIVAAPEGV SLTKLAPQSH FGNIIVFDDP ITLSHSLSSK QVGRAQGFYI YDTKNTYTSW LSFTFVLNST HHQGTITFAG 132
LuDIR1            AAIVAAPEGA NRTILAGEFH FGNIAVFDDP ITLDNNLHSP PVGRAQGMYL YDTKNTFTAW LGFTFSLNST EHQGTINFMG 134
TpDIR5            SALVAAPEGA NLTIMTGNNH FGNLAVFDDP ITLDNNLHSP PVGRAQGFYF YDMKNTFSAW LGFTFVLNST DHKGTITFNG 138
TpDIR8            STLVAAPQGA NLTIMTGNYH FGDLAVFDDP ITVDNNLHSP PVGRAQGFYF YDMKNTFSAW LGFTFVLNST DYKGTITFGG 140

DP1_AtDIR12       ANPFGLQPAR DLSIVGGTGD FLMTRGIATF KTKLTQGSKY FCVEMNIKLY ECY--  185
AtDIR13           ENAF-MEPTR DLPVVGGTGD FVMTRGIATF MTDLVEGSKY FRVKMDIKLY ECYY-  184
AtDIR14           ENPF-MEPTR DLPIVGGTGD FIMTRGIATL TTDHIDGSKY FRVKLDIKLY ECYH-  184
AtDIR5            ADLM-MVQSR DLSVVGGTGD FFMSRGIVTF ETDTFEGAKY FRVKMDIKLY ECY--  182
AtDIR6            ADLM-MEPTR DLSVVGGTGD FFMARGIATF VTDLFQGAKY FRVKMDIKLY ECY--  187
LuDIR5            ADMM-SEKTR DLSVVGGTGD FFMARGIATF RTDTFQGDNY FRLEMDIKLY DCYKY  203
LuDIR6            ADMM-SEETR DLSVVGGTGD FFMARGIATF RTDTFQGDAY FRLEMDIKLY ECY--  196
ScDIR             ADPL-LIKTR DISVIGGTGD FFMARGIATL MTDAFEGEVY FRLRTDIKLY ECY--  195
FiDIR             ADPL-LNKTR DISVIGGTGD FFMARGVATL MTDAFEGDVY FRLRVDINLY ECW--  186
PsDRR206          ADPI-VAKTR DISVTGGTGD FFMHRGIATI TTDAFEGEAY FRLGVYIKFF ECW--  184
LuDIR1            ADPL-MNKTR DVSIVGGTGD FFMHRGVATI MTDSYEGEVY FRLRVDMKFY DCW--  186
TpDIR5            ADPI-LTKYR DISVVGGTGD FLMARGIATI STDSYEGEVY FRLRVNITLY ECY--  190
TpDIR8            ADPI-LAKYR DISVVGGTGD FLMARGIATI DTDAYEGDVY FRLRVNITLY ECY--  192
```

**Supplemental Figure 2.** Multiple alignments of DIRs in DIR-a family.
Conserved amino acid residues in all DIRs were shown in white with black background. Residues conserved in (-)-pinoresinol forming DIRs (magenta) and (+)-pinoresinol forming DIRs (blue) and residues unique to AtDP1 but differentially conserved in (+)-and/or (-)-pinoresinol forming DIRs (green) are also shown. Supporting information for Fig. 6.

```
AtLAC4          M--------- --GSHMVWFL FLVSFFSVFP APSESMVRHY KFNVVMKNVT RLCSSKPTVT
AtLAC10         M--------- ---VFPIRIL VLFALL-AFP ACVHGAIRKY TFNVVTKQVT RICSTKQIVT
AtLAC16         M--------- ---------- ---------- ---------- ------TNTT KLCSSKPIVT
PtLAC3          M--------- ----EYYQAR TMLLVIFIFP ALVECKVRLY NFRVVLTNTT KLCSSKSIPT
AtLAC11         M--------- ---KMGFLFL FCYLLAFLGY SPVDAAVKKY QFDVQVKNIS RICNAKPIVT
AtLAC2          M--------V TWVLNYLLVA FLFAISYNID AASAGITRHY QFDIQLKNIT RLCKTKTIVT
AtLAC17         M--------- --ALQLLLAV FSCVL--LLP QPAFGITRHY TLEIKMQNVT RLCHTKSLVS
SofLAC          M--------- -EAPCLALLL FFGTLLVLPQ SSHG-ATRYY TFNVTLQKVT RLCTTRAIPT
BdLAC5          M--------- -GAKCLSLLV FLGTSLLLPQ LLLAAMTRYY TFNVTMKKVT RLCNTRAIPT
AtLAC1          M--------- ENLGFLIIST FLLLFTTLLP YSSASTTRRF HFNVEWKKVT RLCHTKQLLT
AtLAC3          M--------- -ESFRRFSLL SFIALLAYFA FLASAEHHVH QFVITPTPVK RLCRTHQSIT
AtLAC13         M--------- -EQLRPF--- -FLLLAIFVA SLVNAEVHFH EFVIQETPVK RLCRVHNSIT
AtLAC5          M--------- DVTKSLLCFI SFVAFLLFSS VAEANKAHHH EFIIQATKVK RLCETHNSIT
AtLAC12         M--------- -TTVHTFSIL LFFCSLFSAS LIIA-KVQHH DFVIQETPVK RLCKTRNAIT
AtLAC6          M------TSS AVPSLFRLSF LLFTLQVMNI GRIGAATRFY QFKVQTIRLT RLCQTNEIVT
AtLAC7          M--------- -EGVRVPIAC ALILLAISSI T--SASIVEH TFNVQNLTVS RLCKRQVITV
AtLAC8          M--------P RLHHYLSNQA FLVLLLFSSI A--SAAVVEH VLHIQDVVVK PLCKEQIIPA
AtLAC9          M--------P RVHHSLSNQA FLVLLLFSSI A--SAAIVEH VLHVKDVVVT PLCKEQMIPI
GaLAC1          M--------- -GLQQGLVTW FVGVLFLSTL LLSNADVHHY EFFVRESNFT KLCNTTTLLV
ZmLAC3          MGGGGGGVAK MPAGQLWLLL LGVLLLAFGV PAQASRNTHY DFVITETKVT RLCHEKTILA
AtLAC14         M--EFKLNIP NTIIKTLQTI VFFLFVLLAF QIAEAEIHHH TFKIKSKAYT RLCNTNKILT
AtLAC15/TT10    M--------- -------SHS FFNLFLISLF LYNNCIAHHY TFTVREVPYT KLCSTKAILT
BnTT10          M--------- -------SHP LFIYFLISLS LYSSCTAHRH TFTVKEVPYK KLCSTKKILT

AtLAC4          VNGRYPGPTI YAREDDTLLI KVVNHVKYNV SIHWHGVRQV RTGWADGPAY ITQCPIQPGQ
AtLAC10         VNGKFPGPTI YANEDDTILV NVVNNVKYNV SIHWHGIRQL RTGWADGPAY ITQCPIKPGH
AtLAC16         VNGQFPGPTI VAREGDTILI KVVNHVKYNV SIHWT----- --GWADGPAY ITQCPIQPGQ
PtLAC3          INGKFPGPTI YAREGDNVNI RLTNQVQYNV TVHWHGVSSC FTGWADGPAY ITQCPIQPGQ
AtLAC11         VNGMFPGPTV YAREGDRVII NVTNHVQYNM SIHWHGLKQY RNGWADGPAY ITQCPIQTGQ
AtLAC2          VNGKFPGPRV TAREGDNLQI KVVNHVSNNI SIHWHGIRQL RSGWADGPSY VTQCPIRMGQ
AtLAC17         VNGQFPGPKL IAREGDQVLI KVVNQVPNNI SLHWHGIRQL RSGWADGPAY ITQCPIQTGQ
SofLAC          VNGKFPGPKI VTREGDRVVV KVVNSVKDNI TIHWHGVRQL RTGWSDGPAY VTQCPIRTGQ
BdLAC5          VNGKFPGPKI VTREGDRVVV KVVNNVKHNV TIHWHGVRQL RTGWSDGPAY ITQCPIQTGQ
AtLAC1          VNGQYPGPTV AVHEGDIVEI KVTNRIAHNT TIHWHGLRQY RTGWADGPAY ITQCPIRSKQ
AtLAC3          VNGQYPGPTL VVRNGDSLAI TVINRARYNI SIHWHGIRQL RNPWADGPEY ITQCPIRPGQ
AtLAC13         VNGQFPGPTL EVRNGDSLVI TAINKARYNI SLHWHGIRQM RNPWADGPEY ITQCPIQPGG
AtLAC5          VNGMFPGPML VVNNGDTLVV KVINRARYNI TIHWHGVRQM RTGWADGPEF VTQCPIRPGS
AtLAC12         VNGMFPGPTL EVNNGDTLEV KVHNRARYNI TIHWHGVRQI RTGWADGPEF VTQCPIRPGK
AtLAC6          VNKKFPGPAI SAQEDDRIVI KVINMTPYNT TIHWHGIKQK RSCWYDGPSY ITQCPIQSGQ
AtLAC7          VNGSLPGPTI RVKEGDSLVI HVLNHSPHNI TIHWHGIFHK LTVWADGPSM ITQCPIQPGQ
AtLAC8          ANGSLPGPTI NVREGDTLVV NVINNSTYNV TIHWHGVFQL KSVWMDGANM ITQCPIQPGY
AtLAC9          VNGSLPGPTI NVREGDTLVV HVINKSTYNV TIHWHGVFQL KSVWMDGANM ITQCPIQPSN
GaLAC1          VNDSYPGPEI RVHRGDTVFV NVHNQGNYGF TIHWHGVKQP RNPWSDGPEF VTQCPIQPGT
ZmLAC3          VNGQFPGPTI YARKDDVVIV NVYNQGYKNI TLHWHGVDQP RNPWSDGPEY ITQCPIQPGA
AtLAC14         VNGEFPGPTL KAYRGDKLIV NVINNANYNI TLHWHGARQI RNPWSDGPEY VTQCPIRPGE
AtLAC15/TT10    VNSQFPGPII KVHKGDTIYV NVQNRASENI TMHWHGVEQP RNPWSDGPEY ITQCPIRPGS
BnTT10          VNGRFPGQTL KVYKGDTIYV NVRNRASENI TMHWHGVEQP RNPWSDGPEY ITQCPIRPGS

AtLAC4          VYTYNYTLTG QRGTLWWHAH ILWLRATVYG ALVILPKRGV P-YPFP--KP DNEKVIVLGE
AtLAC10         SYVYNFTVTG QRGTLWWHAH VLWLRATVHG AIVILPKLGL P-YPFP--KP HREEVIILGE
AtLAC16         NYLHNFTLTG QRGTLWWHAH ILWLRATVHG AIVILPKLGV P-YPFP--KP YKEKTIVLSE
PtLAC3          SYLYNFTLTG QRGTLLWHAH ISWLRATIHG AIVIFPKKGV P-YPFP--KP DKEKIIILSE
AtLAC11         SYLYDFNVTG QRGTLWWHAH ILWLRATVYG AIVILPAPGK P-YPFP--QP YQESNIILGE
AtLAC2          SYVYNFTVTG QRGTLWWHAH IQWMRATVYG PLIILPKLHQ P-YPFP--KP YKQVPILFGE
AtLAC17         SYVYNYTIVG QRGTLWYHAH ISWLRSTVYG PLIILPKRGV P-YPFA--KP HKEVPMIFGE
SofLAC          SYVYNFTITG QRGTLFWHAH VSWMRATLYG PIIILPKRGV P-YPFPV-KP YKEVPIIFGE
BdLAC5          SYVYNFTVTG QRGTLFWHAH VSWMRATLYG PIVILPKLGV P-YPFP--KP FKDVPIMFGE
AtLAC1          SYTYRFKVED QRGTLLWHAH HSWQRASVYG AFIIYPRQ-- P-YPFSGSHI QSEIPIILGE
AtLAC3          TYTYRFKIED QEGTLWWHAH SRWLRATVYG ALIIYPRLGS P-YPFS--MP KRDIPILLGE
AtLAC13         SYTYRFTMED QEGTLWWHAH SRWLRATVYG ALIIRPPLSS PHYPFPV-IP KREITLLLGE
AtLAC5          SYTYRFTIQG QEGTLWWHAH SSWLRATVYG SLLVFPPAGS S-YPFT--KP HRNVPLLLGE
```

```
AtLAC12       SYTYRFTIQG QEGTLWWHAH SSWLRATVYG ALIIHPTPGS S-FPFP--KP DRQTALMLGE
AtLAC6        SFTYNFKVAQ QKGTFLWHAH FSWLRATVYG PLIVYPKASV P-YPFK--KP FNEHTILLGE
AtLAC7        RYAYRFNITG QEGTLWWHAH ASFLRATVYG ALVIRPKSGH S-YPFP--KP HKEVPILFGE
AtLAC8        NFTYQFDITG QEGTLLWHAH VVNLRATLHG ALVIRPRSGR P-YPFP--KP YKEVPIVFQQ
AtLAC9        NFTYQFDITG QEGTLLWHAH VVNLRATIHG ALIIRPRSGR P-YPFP--KP YKEVPLIFQQ
GaLAC1        NFTYEIVLSD EIGTLWWHAH SDWTRGSVHG AFVILPAKKE T-YPFP--TP DADQTIILES
ZmLAC3        NFTYKIIFTE EEGTLWWHAH SEFDRATVHG AIVIHPKRGT V-YPYP--KP HKEMPIILGE
AtLAC14       SYVYRIDLKV EEGTIWWHAH SQWARATVHG AFIVYPKRGS S-YPFP--KP HREIPLILGE
AtLAC15/TT10  DFLYKVIFSI EDTTVWWHAH SSWTRATVHG LIFVYPRPPQ I-LPFP--KA DHEVPIILGE
BnTT10        DFIYEVIFST EETTVWWHAH SSWTRATVHG LIFVYPRPPK S-LPFP--KS DHEVPLIFGE

AtLAC4        WWKS-DTENI INEALKSGLA PNVSDSHMIN GHPGPVRNCP SQG-YKLSVE NGKTYLLRLV
AtLAC10       WWKS-DTETV VNEALKSGLA PNVSDAHVIN GHPGFVPNCP SQGNFKLAVE SGKTYMLRLI
AtLAC16       WWKS-DVEEL INEASRIGTA PSASDAHTIN GHSGSISNCP SQSSYGLPVR AGKTYMLRII
PtLAC3        WWKA-DVEAV VNQATMTGLP PNISDAHTVN GHTGAVPGCT SPG-FTLHVE SGKTYLLRII
AtLAC11       WWNK-DVETA VNQANQLGAP PPMSDAHTIN GKPGPLFPCS EKHTFVIEAE AGKTYLLRII
AtLAC2        WFNA-DPQAV VQQALQTGAG PNASDAHTFN GLPGPLYNCS TKDTYKLMVK PGKTYLLRLI
AtLAC17       WFNA-DTEAI IRQATQTGGG PNVSDAYTIN GLPGPLYNCS AKDTFRLRVK PGKTYLLRLI
SofLAC        WFNA-DPEAI IAQALKTGAG PNISDAFTIN GLPGPLYNCS SKDTFKLKVL PGKWYLLRLI
BdLAC5        WFNV-DPEAI IAQALQTGGG PNVSDAYTIN GLPGPLYNCS SRDTFKLKVQ PGKWYLLRLI
AtLAC1        WWND-DVDNV EKAMMKTGAG AKVSDAYTLN GLPGPLYPCS TKDTFTATVD AGKTYILRII
AtLAC3        WWDR-NPMDV LKQAQFTGAA ANVSDAYTIN GQPGDLYRCS RAGTIRFPIF PGETVQLRVI
AtLAC13       WWDR-NPMDV LNLAQFTGAA PNISDAFTIN GQPGDLYRCS SQETLRFLVG SGEIVLLRVI
AtLAC5        WWDA-NPVDV LRESIRTGGA PNNSDAYTIN GQPGDLYKCS SQDTTVVPIN VGETILLRVI
AtLAC12       WWNA-NPVDV INQATRTGAA PNISDAYTIN GQPGDLYNCS TKETVVVPIN SGETSLLRVI
AtLAC6        YWLK-NVVEL EQHVLESGGP PPPADAFTIN GQPGPNYNCS SKDVYEIQIV PRKIYLLRLI
AtLAC7        WWNT-DVVAL EEAAIATGVP PNNSDAYTIN GRPGNLYPCS KDRMFSLNVV KGKRYLLRII
AtLAC8        WWDT-DVRLL QLR------P APVSDAYLIN GLAGDSYPCS ENRMFNLKVV QGKTYLLRIV
AtLAC9        WWDT-DVRLL ELR------P APVSDAYLIN GLAGDSYPCS KNRMFNLKVV QGKTYLLRII
GaLAC1        WYDG-DYKQI IDDALAAGVS PRQPSAYAIS GHVGDTYGCP NDTIFRMQVD SEKIYLLRII
ZmLAC3        WWNA-DVEQI LLESQRTGGD VNISDANTIN GQPGDFAPCS KEDTFKMSVE HGKTYLLRVI
AtLAC14       WWKKENIMHI PGKANKTGGE PAISDSYTIN GQPGYLYPCS KPETFKITVV RGRRYLLRII
AtLAC15/TT10  WWKR-DVREV VEEFVRTGGA PNVSDALTIN GHPGFLYPCS KSDTFHLTVE KGKTYRIRMV
BnTT10        WWKK-DVREV VELFMRTGGD PNVSDALTIN GHPGFLYPCS KSDTFELMVE KSKTYRIRMV

AtLAC4        NAALNEELFF KVAGHIFTVV EVDAVYVKPF KTDTVLIAPG QTTNVLLTAS K----SAGK-
AtLAC10       NAALNEELFF KIAGHRFTVV EVDAVYVKPF NTDTILIAPG QTTTALVSAA R----PSGQ-
AtLAC16       NAALNEELFF KIAGHVLTVV EVDAVYTKPY KTDTVFIAPG QTTNVLLTAN A----NAGSN
PtLAC3        NAALNDELFF KIAGHNITVV EVDATFTKPF STDTIFIGPG QTTNALLTAD K----SIGK-
AtLAC11       NAALNDELFF GIAGHNMTVV EIDAVYTKPF TTKAILLGPG QTTNVLVKTD R----SPNR-
AtLAC2        NAALNDELFF TIANHTLTVV EADACYVKPF QTNIVLLGPG QTTNVLLKTK P--I-YPNAT
AtLAC17       NAALNDELFF SIANHTVTVV EADAIYVKPF ETETILIAPG QTTNVLLKTK S--S-YPSAS
SofLAC        NAALNDELFF SIANHTLTVV DVDAAYVKPF HTDVVLITPG QTTNVLLRAE PDAG-CPAAT
BdLAC5        NAALNDELFF SIANHTLTIV DVDASYVKPF DTDVVLVTPG QTTNVLLHAK PDEG-CQPAT
AtLAC1        NAALNNELFV AVANHTLTVV EVDAVYTKPV HTKAIMIAPG QTTTLLLRAD Q----LSGGE
AtLAC3        NAGMNQELFF SVANHQFTVV ETDSAYTKPF TTNVIMIGPG QTTNVLLTAN Q----RPG-R
AtLAC13       NSALNQELFF GVANHKLTVV AADASYTKPF STNVIMLGPG QTTDVLLTAD Q----PPA-H
AtLAC5        NSALNQPLFF TVANHKLTVV GADASYLKPF TTNVIVLGPG QTTDVLITGD Q----PPN-R
AtLAC12       NAALNQPLFF TVANHKLTVV GADASYLKPF TTKVLMLGPG QTTDVLLTAD Q----PPK-R
AtLAC6        NAGINMETFF TIANHRLTIV EVDGEYTKPY TTERVMLVPG QTMNILVTAD Q----TVG-R
AtLAC7        NAAMNIQLFF KIANHRLTVV AADAVYTAPY VTDVIVIAPG QTIDALLFAD Q----SVDTS
AtLAC8        NAALNTHLFF KIANHNVTVV AVDAVYSTPY LTDVMILTPG QTVDALLTAD Q----AIG-K
AtLAC9        NAALNTHLFF KIANHNVTVV AVDAVYTTPY LTDVMILTPG QTIDAILTAD Q----PIG-T
GaLAC1        NAAMNEHFFF TIANHTLTVV AQDASYVRRF TRDYILISPG QTMDVLVSAN R----NVG-Q
ZmLAC3        NAGLTNEMFF AVAGHRLTVV GTDGRYLRPF TVDYILISPG QTMNMLLEAN CATDGSANSR
AtLAC14       NAVMDEELFF AIANHTLTVV AKDGFYLKHF KSDYLMITPG QSMDVLLHAN Q----RPN-H
AtLAC15/TT10  NAAMNLPLFF AIANHSLTVV SADGHYIKPI KATYITISPG ETLDMLLHAD Q----DPERT
BnTT10        NAALNLILFF AIAKHNLTVV AADGHYTKPI NATYITISPG QTLDLLLHAD Q----NPKST

AtLAC4        YLVTASPFMD -APIAVDNVT ATATVHYSG- --------TL S----SSPTI LTLPPPQN--
AtLAC10       YLIAAAPFQD SAVVAVDNRT ATATVHYSG- --------TL S----ATPTK TTSPPPQN--
```

```
AtLAC16         YMVAATTFTD -AHIPYDNVT ATATLHYIG- --------HT STVSTSKKTV LASLPPQN--
PtLAC3          YLIAVSPFMD -TVVAVDNVT AIAFLRYNE- --------PL H----SPHLS LTTTPAIN--
AtLAC11         YFMAASPFMD -APVSVDNKT VTAILQYKG- --------VP N----TVLPI LPKLPLPN--
AtLAC2          FYMLARPYFT G-QGTIDNTT VAGILQYQ-- --------HH TKSSKNLSII KPSLPPIN--
AtLAC17         FFMTARPYVT G-QGTFDNST VAGILEYEPP KQTKG--AHS RTSIKNLQLF KPILPALN--
SofLAC          HLMLARPYGT GQPGTFDNTT VAAVLEYA-- ---------P PGHIKSLPLF RPSLPALN--
BdLAC5          HLMLARPYAT SRPGTYDNTT VAAVLEYS-- ---------P SGQIKSRPLF RPTLPVFN--
AtLAC1          FLIAATPYVT -SVFPFNNST TVGFIRYTGK TKPENSVNTR RRRRLTAMST VVALPNML--
AtLAC3          YYMAARAYNS AN-APFDNTT TTAILQYVNA PTRRG----- -RGRGQIAPV FPVLPGFN--
AtLAC13         YYMAAHAYNS AN-AAFDNTT TTAILKYKDA SCVTL----- -QAKSQARAI PAQLPGFN--
AtLAC5          YYMAARAYQS AQNAPFGNTT TTAILQYKSA PCCGVGGGSG TKKGNSFKPI MPILPAYN--
AtLAC12         YYIAARAYQS AQNAPFDNTT TTAILQYK-- ---------- -KTTTTSKPI MPVLPAFN--
AtLAC6          YSMAMGPYES AKNVKF--QN TSAIANFQYI G--------- --ALPNNVTV PAKLPIFN--
AtLAC7          YYMAAHPYAS APAVPF-PNT T-TRGVIHYG G--------- --ASKTGRSK PVLMPKLPSF
AtLAC8          YYMATLPYIS AIGIPT-PDI KPTRGLIVYQ G--------- --ATSSSSPA EPLMPVPN--
AtLAC9          YYMAIIPYFS AIGVPASPDT KPTRGLIVYE G--------- --ATSSSSPT KPWMPPAN--
GaLAC1          YYMAIRPFSD SSAAPVDNIT T-GIFEYTN- ---------- --SEGGLNAS LITLPVMN--
ZmLAC3          YYMAARPFFT NTAVNVDDKN TTAIVEYTDA P--------- -PSASAGPPD SPDLPAMD--
AtLAC14         YFVAARAYSS AFGAGFDKTT TTAILQYKGD T--------- ---LNRIKPI LPYLPPYN--
AtLAC15/TT10    YYMAARAYQS G-NIDFNNST TIGILSYTSS CK-------- -AKTSSFSGY YPTLPFYN--
BnTT10          YYMAARAYHS NPNINFNNST TIGILRYTSS ---------- ---TSSSSKR YPNLPYYN--

AtLAC4          -ATSIANNFT NSLR-SLNSK KYPALVP--T TIDHHLFFTV GLGLNACPT- -----CKAGN
AtLAC10         -ATSVANTFV NSLR-SLNSK TYPANVP--I TVDHDLLFTV GLGINRCHS- -----CKAGN
AtLAC16         -ATWVATKFT RSLR-SLNSL EYPARVP--T TVEHSLFFTV GLGANPCQS- -----CNNG-
PtLAC3          -ATPATSTFM DKLR-SLNSK KYPANVP--L TVDHDLYFTI GVGIDPCAT- -----CTNG-
AtLAC11         -DTSFALDYN GKLK-SLNTP NFPALVP--L KVDRRLFYTI GLGINACPT- -----CVNG-
AtLAC2          -STSYAANFT KMFR-SLASS TFPANVP--K VVDKQYFFAI GLGTNPCPK- -NQTCQGPTN
AtLAC17         -DTNFATKFS NKLR-SLNSK NFPANVP--L NVDRKFFFTV GLGTNPCNHK NNQTCQGPTN
SofLAC          -DTAFAANYS ARLR-SLATP DYPANVP--R GVDRSFFFAV GLGTNPCPA- -NQTCQGP-N
BdLAC5          -DTSFAANYS AKHR-SLASS EYPANVP--R RIDRPFFFAV GLGTTPCPT- -HQGCNGPTN
AtLAC1          -DTKFATKFS DSIK-SLGSA KYPCKVP--T KIDKRVITTI SLNLQDCPLN ---QTCDGYA
AtLAC3          -DTATATAFT NRLR-YWK-- --RAPVP--Q QVDENLFFTV GLGLINCANP N-SPRCQGPN
AtLAC13         -DTATAAAFT AQMK-SPS-- --KVKVP--L EIDENLFFTV GLGLFNCPTP N-TQRCQGPN
AtLAC5          -DTNTVTRFS QSFR-SLR-- --RAEVP--T EIDENLFVTI GLGLNNCPKN FRSRRCQGPN
AtLAC12         -DTNTVTSFS RKFK-SLR-- --NVVVP--K TIDDNLFFTI GLGLDNCPKK FPKSRCQGLN
AtLAC6          -DNIAVKTVM DGLR-SLN-- --AVDVP--R NIDAHLFITI GLNVNKCNSE NPNNKCQGPR
AtLAC7          FDTLTAYRFY SNLT-ALVNG PHWVPVP--R YVDEEMLVTI GLGLEACAD- --NTTCP---
AtLAC8          -DMSTAHRFT SNIT-SLVGG PHWTPVP--R HVDEKMFITM GLGLDPCPA- --GTKCIGPL
AtLAC9          -DIPTAHRFS SNIT-SLVGG PHWTPVP--R HVDEKMFITM GLGLDPCPS- --NAKCVGPL
GaLAC1          -DTDAMINFL NQIRNTKVSQ NPRINVPADK DIKRRVFMTL AVNNLPCNT- -------CVV
ZmLAC3          -DIAAATAYT AQLR-SLVTK EHPIDVP--M EVDEHMLVTI SVNTIPCEPN ---KTCAGPG
AtLAC14         -RTEASTRFT NQFR-SQR-- --PVNVP--V KINTRLLYAI SVNLMNCSDD R---PCTGPF
AtLAC15/TT10    -DTSAAFGFF TKIK-CLF-- --SGQVP--V QISRRIITTV SINLRMCPQN ----SCEGPN
BnTT10          -DTSAAFRFF TSIK-CLY-- --SGQVP--V KISRRIISTV SINNLMCPNN ----LCEGPN

AtLAC4          GSRVVASINN VTFIMPKT-A LLPAHYF-NT SGVFTTDFPK NPPHVFNYSG -------GSV
AtLAC10         FSRVVAAINN ITFKMPKT-A LLQAHYF-NL TGIYTTDFPA KPRRVFDFTG -------KPP
AtLAC16         -VRLVAGINN VTFTMPKT-A LLQAHFF-NI SGVFTDDFPA KPSNPYDYTA P-----VKLG
PtLAC3          -SKAVADINN VSFIMPTT-A LLQAHYY-NI SGVFTDDFPA KPPISFNYTG ------NNTA
AtLAC11         -TNLAASINN ITFIMPKT-A LLKAHYS-NI SGVFRTDFPD RPPKAFNYTG ------VPLT
AtLAC2          TTKFAASINN VSFILPNKTS LLQSYFVGKS KNVFMTDFPT APIIPFNYTG -------TPP
AtLAC17         TTMFAASISN ISFTMPTK-A LLQSHYSGQS HGVYSPKFPW SPIVPFNYTG -------TPP
SofLAC          GSMFTASMNN VSFDMPTT-A LLQAHYN-NI AGVYTTDFPV APLEPFNYTG -------TPP
BdLAC5          DTKFSASMNN VSFNMPTT-A LLKAHYDGNT AGVYTADFPA MPTQPFNYTG -------TPP
AtLAC1          GKRFFASMNN ISFVRPPI-S ILESYYKKQS KGVFSLDFPE KPPNRFDFTG V-----DPVS
AtLAC3          GTRFAASMNN MSFVLPRSNS VMQAYYQGTP -GIFTTDFPP VPPVQFDYTG ------NVSR
AtLAC13         GTRFTASINN VSFVFPKQNS IMQAYYQGTP TGVFTTDFPP TPPVTFDYTG ------NVSR
AtLAC5          GTRFTASMNN VSFALPSNYS LLQAHHHGIP -GVFTTDFPA KPPVKFDYTG N-----NISR
AtLAC12         GTRFTASMNN VSFVLPSNFS LLQAHSNGIP -GVFTTDFPS KPPVKFDYTG N-----NISR
AtLAC6          KGRLAASMNN ISFIEPK-VS ILEAYYKQLE -GYFTLDFPT TPEKAYDFVN GAPNDIANDT
```

```
AtLAC7        --KFSASMSN HSFVLPKKLS ILEAVFHDVK -GIFTADFPD QPPVKFDYTN --PNVTQTNP
AtLAC8        GQRYAGSLNN RTFMIPERIS MQEAYFYNIS -GIYTDDFPN QPPLKFDYTK FEQR-TNNDM
AtLAC9        DQRLAGSLNN RTFMIPERIS MQEAYFYNIT -GVYTDDFPD QPPLKFDFTK FEQHPTNSDM
GaLAC1        GSRLVASLNN VSYVSPS-ID ILQAYYNRNM SGVYTEDFPL NPPVIYDFTG ------NLTN
ZmLAC3        NNRLAASLNN VSFMNPTI-D ILDAYYDSIS -GVYEPDFPN KPPFFFNFTA P-----NPPQ
AtLAC14       GKRFSSSINN ISFVNPS-VD ILRAYYRHIG -GVFQEDFPR NPPTKFNYTG E-----NLPF
AtLAC15/TT10  GSRLAASMNN ISFVTPSHVD ILKAYYYHIK -GVYGTRFPE FPPLIFNFTA E-----NQPL
BnTT10        GSRLAASMNN ISFVTPSHVD ILKAYYYHIR -GVYGTRFPE FPPLVFNFTA D-----DQPL

AtLAC4        TNMATETGTR LYKLPYNATV QLVLQDTGVI APEN-HPVHL HGFNFFEVGR GLGNFNSTK-
AtLAC10       SNLATMKATK LYKLPYNSTV QVVLQDTGNV APEN-HPIHL HGFNFFVVGL GTGNYNSKK-
AtLAC16       VNAATMKGTK LYRLPYNATV QIVLQNTAMI LSDN-HPFHL HGFNFFEVGR GLGNFNPEK-
PtLAC3        MNLKTTNGTR AYRLAFNSAV QVVLQGTTII APES-HPFHL HGFNFFVVGK GIGNFDPDN-
AtLAC11       ANLGTSTGTR LSRVKFNTTI ELVLQDTNLL TVES-HPFHL HGYNFFVVGT GVGNFDPKK-
AtLAC2        NNTMVSRGTK VVVLKYKTTV ELVLQGTSIL GIEA-HPIHL HGFNFYVVGQ GFGNFNPAR-
AtLAC17       NNTMVSNGTN LMVLPYNTSV ELVMQDTSIL GAES-HPLHL HGFNFFVVGQ GFGNFDPNK-
SofLAC        NNTNVSSGTK VVVLEYNTSV EVVLQGTSIL GAES-HPLHL HGFDFFVVGQ GFGNYDSSK-
BdLAC5        NNTNVSNGTK VAVLPYNASV EVVLQDTSIQ GAES-HPLHL HGFDFFVVGQ GVGNYNASM-
AtLAC1        ENMNTEFGTK LFEVEFGSRL EIVFQGTSFL NIEN-HPLHV HGHNFFVVGR GFGNFDPEK-
AtLAC3        GLWQPIKGTK AYKLKYKSNV QIVLQDTSIV TPEN-HPMHL HGYQFYVVGS GFGNFNPRT-
AtLAC13       GLWQPTRGTK AYKLKFNSQV QIILQDTSIV TTEN-HPMHL HGYEFYVVGT GVGNFNPNT-
AtLAC5        SLYQPDRGTK LYKLKYGSRV QIVLQDTGIV TPEN-HPIHL HGYDFYIIAE GFGNFNPKK-
AtLAC12       ALFQPVKGTK LYKLKYGSRV QVVLQDTNIV TSEN-HPIHL HGYDFYIVGE GFGNFNPKK-
AtLAC6        QAAN---GTR AIVFEYGSRI QIIFQNTGTL TTEN-HPIHL HGHSFYVIGY GTGNYD--Q-
AtLAC7        GLLFTQKSTS AKILKFNTTV EVVLQNHALI AAES-HPMHL HGFNFHVLAQ GFGNYDPSR-
AtLAC8        KMMFPERKTS VKKIRFNSTV EIVLQNTAII SPES-HPMHL HGFNFYVLGY GFGNYDPIR-
AtLAC9        EMMFPERKTS VKTIRFNSTV EIVLQNTGIL TPES-HPMHL HGFNFYVLGY GFGNYDPIR-
GaLAC1        LNTPVEEGTR VIVVNYGEGV EMVLQATQMG AGGS-HPIHL HGSSFYWVGT GFGNFNNKT-
ZmLAC3        DLWFTKRGTK VKVVEYGTIL EVVFQDTAIL GAES-HPMHL HGFSFYVVGR GFGNFDKDK-
AtLAC14       P---TRFGTK VVVLDYNSSV ELILQGTTVW ASNI-HPIHL HGYNFYVVGS GFGNFDRRK-
AtLAC15/TT10  FLETPRLATE VKVIEFGQVV ELVIQGTSLV GGGLDHPMHL HGFSFYVVGV GFGNYNISEE
BnTT10        FLQTPRFATE VKILKFGESV EIVLQGTSLV GGGIDHPMHL HGFSFYEVGV GFGNYNVTE-

AtLAC4        DPKN-FNLVD PVERNTIGVP SGGWVVIRFR ADNPGVWFMH CHLEVHTTWG LKMAFLVENG
AtLAC10       DSNK-FNLVD PVERNTVGVP SGGWAAIRFR ADNPGVWFMH CHLEVHTTWG LKMAFLVENG
AtLAC16       DPKA-FNLVD PVERNTVGVP AGGWTAIRFI ADNPGVWFMH CHLELHTTWG LKMAFVVDNG
PtLAC3        DPKK-FNLAD PVERNTVSVP TAGWIAIRFK ADNPGVWFLH CHLEVHTTWG LKMAFVVDNG
AtLAC11       DPAK-FNLVD PPERNTVGVP TGGWAAIRFR ADNPGVWFMH CHLEVHTMWG LKMAFVVENG
AtLAC2        DPKH-YNLVD PVERNTINIP SGGWVAIRFL ADNPGVWLMH CHIEIHLSWG LTMAWVVLDG
AtLAC17       DPRN-FNLVD PIERNTVGVP SGGWAAIRFL ADNPGVWFMH CHLEVHTSWG LRMAWLVLDG
SofLAC        DPAN-FNLVD PVQRNTVGVP SAGWVAIRFF ADNPGVWFMH CHLEVHTAWG LKMAWVVNDG
BdLAC5        HPAG-FNLLD PVQRNTVGVP AGGWVAIRFY ADNPGVWFMH CHLEVHTSWG LKMAWVVNDG
AtLAC1        DPKR-YNLVD PPERNTFAVP TGGWAAIRIN ADNPGVWFIH CHLEQHTSWG LAMGFIVKDG
AtLAC3        DPAR-FNLFD PPERNTIGTP PGGWVAIRFV ADNPGAWFMH CHIDSHLGWG LAMVFLVENG
AtLAC13       DTSS-FNLID PPRRNTIGTP PGGWVAIRFV ANNPGAWLMH CHIDSHIFWG LAMVFLVENG
AtLAC5        DTAK-FNLED PPLRNTVGVP VNGWAVIRFI ADNPGVWIMH CHLDAHISWG LAMAFLVENG
AtLAC12       DTSK-FNLVD PPLRNTVAVP VNGWAVIRFV ADNPGVWLMH CHLDVHIKWG LAMAFLVDNG
AtLAC6        QTAK-FNLED PPYLNTIGVP VGGWAAIRFV ANNPGLWLLH CHFDIHQTWG MSTMFIVKNG
AtLAC7        DRSK-LNLVD PQSRNTLAVP VGGWAVIRFT ANNPGAWIFH CHIDVHLPFG LGMIFVVKNG
AtLAC8        DARK-LNLFN PQMHNTVGVP PGGWVVLRFI ANNPGVWLFH CHMDAHLPYG IMSAFIVQNG
AtLAC9        DARK-LNLFN PQMHNTVGVP PGGWVVLRFI ANNPGIWLFH CHMDAHLPLG IMMAFIVQNG
GaLAC1        DPRT-YNLVD PPLINTVHVP GRRWVAIRFF ATNPGVWFMH CHLERHSSWG MDTVLIVRNG
ZmLAC3        DPAT-YNLVD PPYQNTVSVP TGGWAAMRFR AANPGVWFMH CHFDRHTVWG MDTVFIVKNG
AtLAC14       DPLR-YNLVD PPEETTVGVP RNGWTAVRFV ANNPGVWLLH CHIERHATWG MNTVFIVKDG
AtLAC15/TT10  DPSSRYNLYD PPYKNTMTVP RNGWIAIRFV ADNPGVWFMH CHLDRHQTWG MNVVFIVKNG
BnTT10        DPSN-YNLKD PPYINTATVP RNGWVAIRFI ADNPGVWFMH CHFDRHLTWG MKVVFIVMNG

AtLAC4        KGPNQSILPP PKDLPKC--- ---------- ---------- ---------- ----------
AtLAC10       KGPNQSIRPP PSDLPKC--- ---------- ---------- ---------- ----------
AtLAC16       HGPDQSLLPP PADLPKC--- ---------- ---------- ---------- ----------
PtLAC3        KGPNESILPP PSDLPTC--- ---------- ---------- ---------- ----------
```

```
AtLAC11         ETPELSVLPP PKDYPSC--- ---------- ---------- ---------- ----------
AtLAC2          DLPNQKLLPP PSDFPKC--- ---------- ---------- ---------- ----------
AtLAC17         DKPDQKLLPP PADLPKC--- ---------- ---------- ---------- ----------
SofLAC          PLPEQKLMPP PPDLPKC--- ---------- ---------- ---------- ----------
BdLAC5          PLPDQKLMPP PSDLPKCVPA GGWVAIRFFA DNPGVWFMHC HLEVHTSWGL KMAWVVNDGP
AtLAC1          PLPSQTLLPP PHDLPQC--- ---------- ---------- ---------- ----------
AtLAC3          RGQLQSVQAP PLDLPRC--- ---------- ---------- ---------- ----------
AtLAC13         EGHLQSVQSP PLDLPQC--- ---------- ---------- ---------- ----------
AtLAC5          NGVLQTIEQP PHDLPVC--- ---------- ---------- ---------- ----------
AtLAC12         VGELETLEAP PHDLPIC--- ---------- ---------- ---------- ----------
AtLAC6          KKVQESLPHP PADLPKC--- ---------- ---------- ---------- ----------
AtLAC7          PTKSTTLPPP PPDLPKC--- ---------- ---------- ---------- ----------
AtLAC8          PTPETSLPSP PSNLPQCTRD PTIYDSRTTN IDLSY----- ---------- ----------
AtLAC9          PTRETSLPSP PSNLPQCTRD PTIYDSRTTN VDMSY----- ---------- ----------
GaLAC1          KTKKTSIRPP PSTMPRCPGT ---------- ---------- ---------- ----------
ZmLAC3          KGPDAQMMPR PPNMPKC--- ---------- ---------- ---------- ----------
AtLAC14         PTKSSRMVKP PPDLPSC--- ---------- ---------- ---------- ----------
AtLAC15/TT10    REPNQQILPP PDDLPPCYE- ---------- ---------- ---------- ----------
BnTT10          RGLNQQILPP PPNLPPCY-- ---------- ---------- ---------- ----------

AtLAC4          ---------- ------
AtLAC10         ---------- ------
AtLAC16         ---------- ------
PtLAC3          ---------- ------
AtLAC11         ---------- ------
AtLAC2          ---------- ------
AtLAC17         ---------- ------
SofLAC          ---------- ------
BdLAC5          LPEQKLMPPP ADLPMC
AtLAC1          ---------- ------
AtLAC3          ---------- ------
AtLAC13         ---------- ------
AtLAC5          ---------- ------
AtLAC12         ---------- ------
AtLAC6          ---------- ------
AtLAC7          ---------- ------
AtLAC8          ---------- ------
AtLAC9          ---------- ------
GaLAC1          ---------- ------
ZmLAC3          ---------- ------
AtLAC14         ---------- ------
AtLAC15/TT10    ---------- ------
BnTT10          ---------- ------
```

**Supplemental Figure 3**. Multiple alignments of LACs.

Conserved amino acid residues in all DIRs are shown in white with a black background.

Supporting information for Fig. 9.

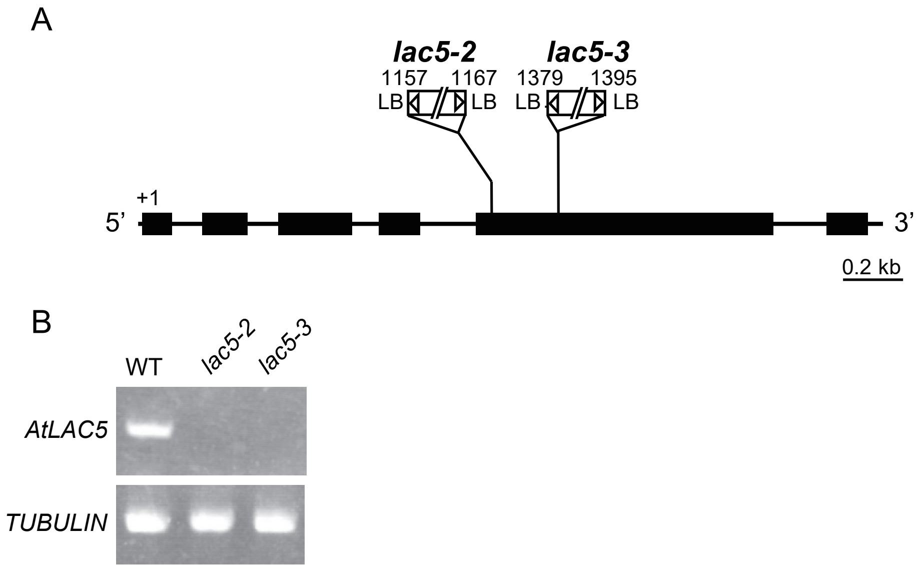


Supplemental Figure 4. T-DNA insertion mutants of *AtLAC5*

(A) Schematic representation of *AtLAC5* with two T-DNA insertion mutants used in this work. The thick line indicates exons and the thinner line indicates introns and 5'- and 3'-untranslated regions. White triangles show left borders (LBs). Numbers indicate the position of the T-DNA insertion. (B) Reverse transcription polymerase chain reaction (RT-PCR) analysis of transcripts in wild type (WT, Col-0) and two independent homozygous mutant lines (*lac5-2* and *lac5-3*). Supporting information for Fig. 5.

At2g40370 *AtLAC5*

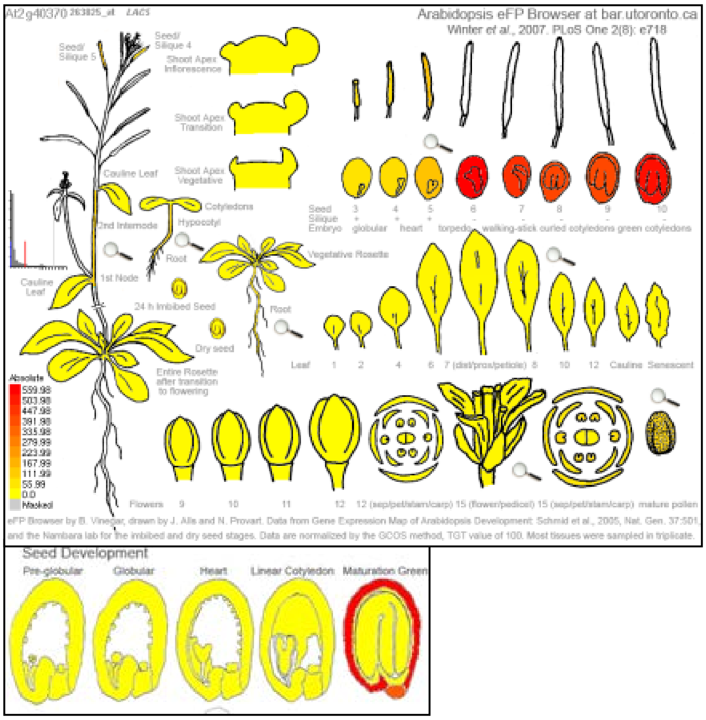


At2g40370 *AtLAC5*

At4g11180 *AtDP1/AtDIR12*

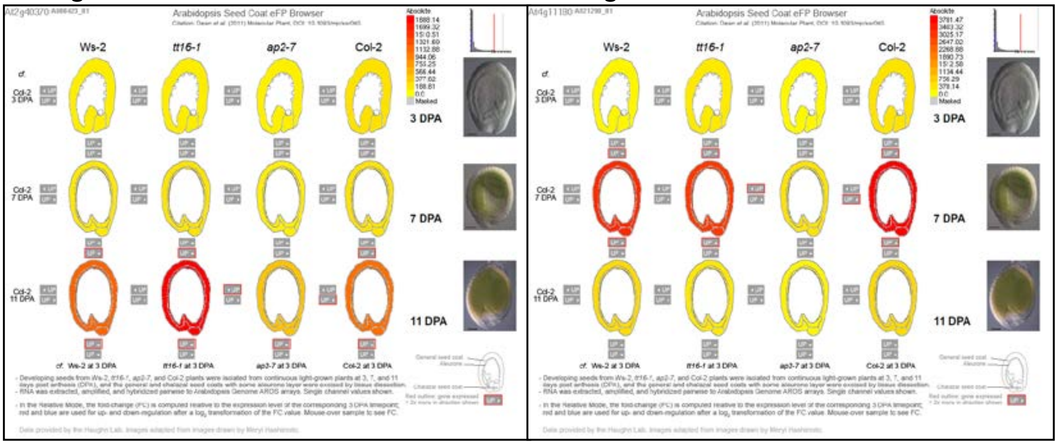


**Supplemental Figure 5**. Expression pattern of *AtLAC5* and *AtDP1/AtDIR12* using the Arabidopsis eFP browser (Winter et al., 2007) and Arabidopsis Seed Coat eFP browser (Dean et al., 2011). Supporting information for Fig. 8.

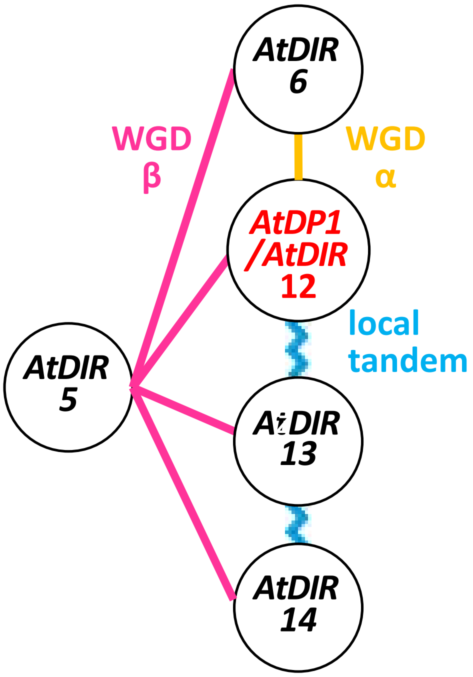


Supplemental Figure 6. A proposed model for gene duplication of Arabidopsis DIR-a gene family. The gene duplication modes were proposed by Wang *et al*., 2013. WGD, whole genome duplication. Supporting information for Fig. 6.

Supplemental Table 1. Metabolites detected in seeds of wild type and the tested mutants

| No. | Metabolite | Annotation Level[a] | Elemental Composition | Retention Time[d] min | Quantifer Ion *m/z* |
|---|---|---|---|---|---|
| 1 | SC(4-*O*-8)G | 1[b] | $C_{26}H_{36}NO_9^+$ | 7.6252 | 506.2386 |
| 2 | SC(4-*O*-8)G 4-*O*-Hex | 2[c] | $C_{32}H_{46}NO_{14}^+$ | 6.1173 | 668.2938 |
| 3 | SC(4-*O*-8)S | 2[c] | $C_{27}H_{38}NO_{10}^+$ | 7.5309 | 536.2493 |
| 4 | FC(4-*O*-8)G | 2[c] | $C_{25}H_{34}NO_8^+$ | 6.5656 | 476.2277 |
| 5 | FC(4-*O*-8)S | 2[c] | $C_{26}H_{36}NO_9^+$ | 6.4285 | 506.2386 |
| 6 | FC(5-8)G | 2[c] | $C_{25}H_{32}NO_7^+$ | 9.2729 | 458.2174 |
| 7 | FC(5-8)G 4-*O*-Hex | 2[c] | $C_{31}H_{42}NO_{12}^+$ | 7.2137 | 620.2712 |
| 8 | SC 4-*O*-glucoside | 2[c] | $C_{22}H_{34}NO_{10}^+$ | 4.2665 | 472.2176 |
| 9 | vanilloylcholine 4-*O*-Hex isomer#1 | 2[c] | $C_{19}H_{30}NO_9^+$ | 1.0079 | 416.1915 |
| 10 | vanilloylcholine 4-*O*-Hex isomer#2 | 2[c] | $C_{19}H_{30}NO_9^+$ | 2.8700 | 416.1916 |
| 11 | 5-hydroxy-FC isomer #1 | 2[c] | $C_{15}H_{22}NO_5^+$ | 4.6977 | 296.1497 |
| 12 | 5-hydroxy-FC isomer #1 | 2[c] | $C_{15}H_{22}NO_5^+$ | 5.0834 | 296.1498 |
| 13 | benzoylcholine | 2[c] | $C_{12}H_{18}NO_2^+$ | 5.3346 | 208.1337 |
| 14 | FC | 2[c] | $C_{15}H_{22}NO_4^+$ | 5.8858 | 280.1549 |
| 15 | SC isomer #1 | 2[c] | $C_{16}H_{24}NO_5^+$ | 5.9887 | 310.1658 |
| 16 | SC isomer #2 | 2[c] | $C_{16}H_{24}NO_5^+$ | 6.3685 | 310.1653 |

[a]Annotation level according to Sumner et al. (2007). [b]Compound indentified with a chemically synthesized standard. [c]Compound annnotated based on Clauß et al. (2011). [d]LC condition for analyses of neolignans and choline derivatives. G, Guaiacyl moiety; S, Syringyl moiety; SC, sinapoylcholine; FC, feruloylcholine; Hex, hexose.

**Sumner, L.W., Amberg, A., Barrett, D., Beale, M.H., Beger, R., Daykin, C.A., Fan, T.W.M., Fiehn, O., Goodacre, R., Griffin, J.L., Hankemeier, T., Hardy, N., Harnly, J., Higashi, R., Kopka, J., Lane, A.N., Lindon, J.C., Marriott, P., Nicholls, A.W., Reily, M.D., Thaden, J.J., Viant, M.R.** (2007). Proposed minimum reporting standards for chemical analysis Chemical Analysis Working Group (CAWG) Metabolomics Standards Initiative (MSI). Metabolomics **3:** 211–221.

**Clauß, K., von Roepenack-Lahaye, E., Böttcher, C., Roth, M.R., Welti, R., Erban, A., Kopka, J., Scheel, D., Milkowski, C., Strack, D.** (2011). Overexpression of Sinapine Esterase *BnSCE3* in Oilseed Rape Seeds Triggers Global Changes in Seed Metabolism. Plant Physiol. **155:** 1127-1145.

**Supplemental Table 2.** Primers used in this study.

| Primer name | Sequence |
|---|---|
| At4g11180-6818953f | 5′- GCATATTGGCATGTCTGTATC-3′ |
| At4g11180-6819803r | 5′- TGGTTCAACTATCATTCGTATTAC-3′ |
| At2g40370/886f | 5′-AATAACTCAGACGCTTACACCATC-3′ |
| At2g40370/1826r | 5′-GCTTATGTTGTTACCAGTGTAATC-3′ |
| LBa1 | 5′-GTGATGGTTCACGTAGTGGGCCATC-3′ 5′- |
| RBa1 | TTGGATTGAGAGTGAATATGAGACTCT-3′ 5′- |
| At4g11180-14f | ACAATGACAAATCAAATCTACAAAC-3′ 5′- |
| At4g11180-607r | GCCAACACACGAAGATCAATC-3′ |
| At2g40370/875f | 5′-GACGCTTACACCATCAATGGTC-3′ |
| gDP1-Rv2 | 5′-TTATGCAGCGCTCTTGGTTGAAGA-3′ |
| CACC-gDP1-Fw1 | 5′-CACCGTAAGTTCTCAATTTCACACCACT-3′ |
| CACC-gDP1-Fw2 | 5′-CACCTAGTAGTTGCTGAAATCTTTAGGT-3′ |
| At4g11180cDNA-RT101f | 5′-GACCTAAAGAAACCATGCAAGCA-3′ |
| At4g11180cDNA-RT186r | 5′-GCATTTGCCGCGTTATCAC-3′ |
| At1g61720pda14333_RT107f | 5′-AGCTGCTGCTGTTTCCATCA-3′ |
| At1g61720pda14333_RT179r | 5′-AACGTCAGTCCAGTTTTCTTCGT-3′ |
| At5g48100pda12625_RT478f | 5′-CCAAAGGCAGACCATGAAGTC-3′ |
| At5g48100pda12625_RT552r | 5′-CTCCTCAACGACCTCTCTCACA-3′ |
| At2g40370qPCRNo2_1090F | 5′-GAGCTGAGGTTCCGACAGAAA -3′ |
| At2g40370qPCRNo2_1107R | 5′-GACCGAGTCCGATGGTTACG-3′ |
| At4g11180_CACC+promoterF | 5′-CACCTAGTTGCTGAAATCTT-3′ |
| At4g11180_promoter R | 5′-TGTTAGAGTGTTAAGTAGAAATG-3′ |
| At4g11180_R | 5′-GTAACATTCATAAAGTTTAATATTC-3′ |
| At2g40370pro_CACC+4140F | 5′-CACCGAAGCTCGAATTTGATGATT-3′ |
| At2g40370pro_CACC+2000F | 5′-CACCTGGAAAGTTGACCTTACGGT-3′ |
| At2g40370ATG_R | 5′-CATTGATGTGATTTCTGTAAGCTTCTG-3′ |
| At5g09640_SCPL19_-8F | 5′-GTAGCAACACTAAGAAGAAG-3′ |
| At5g09640_SCPL19_2519R | 5′-TATACTCCGCTGTGTGTCCT-3′ |
