# Supplementary material for: Seed-coat protective neolignans are produced by the dirigent protein AtDP1 and the laccase AtLAC5 in Arabidopsis
Source: Plant Cell. 2020 Nov 27;33(1):129–52. doi: 10.1093/plcell/koaa014 (PMC8136895; doi:10.1093/plcell/koaa014)
Supplement: koaa014_Supplementary_Data [file koaa014_supplementary_data.zip › tpc.00658.2020-s06.pdf]

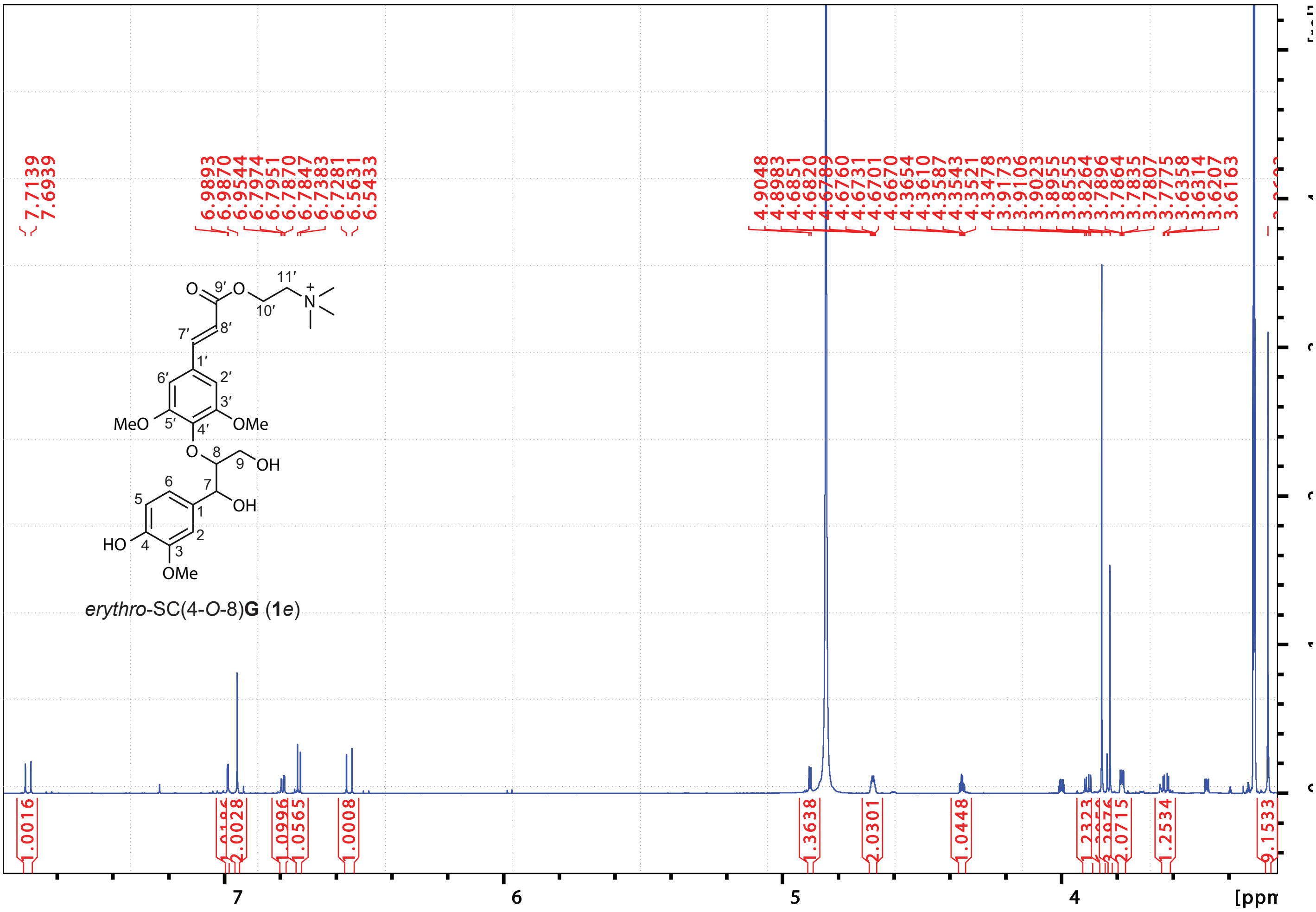

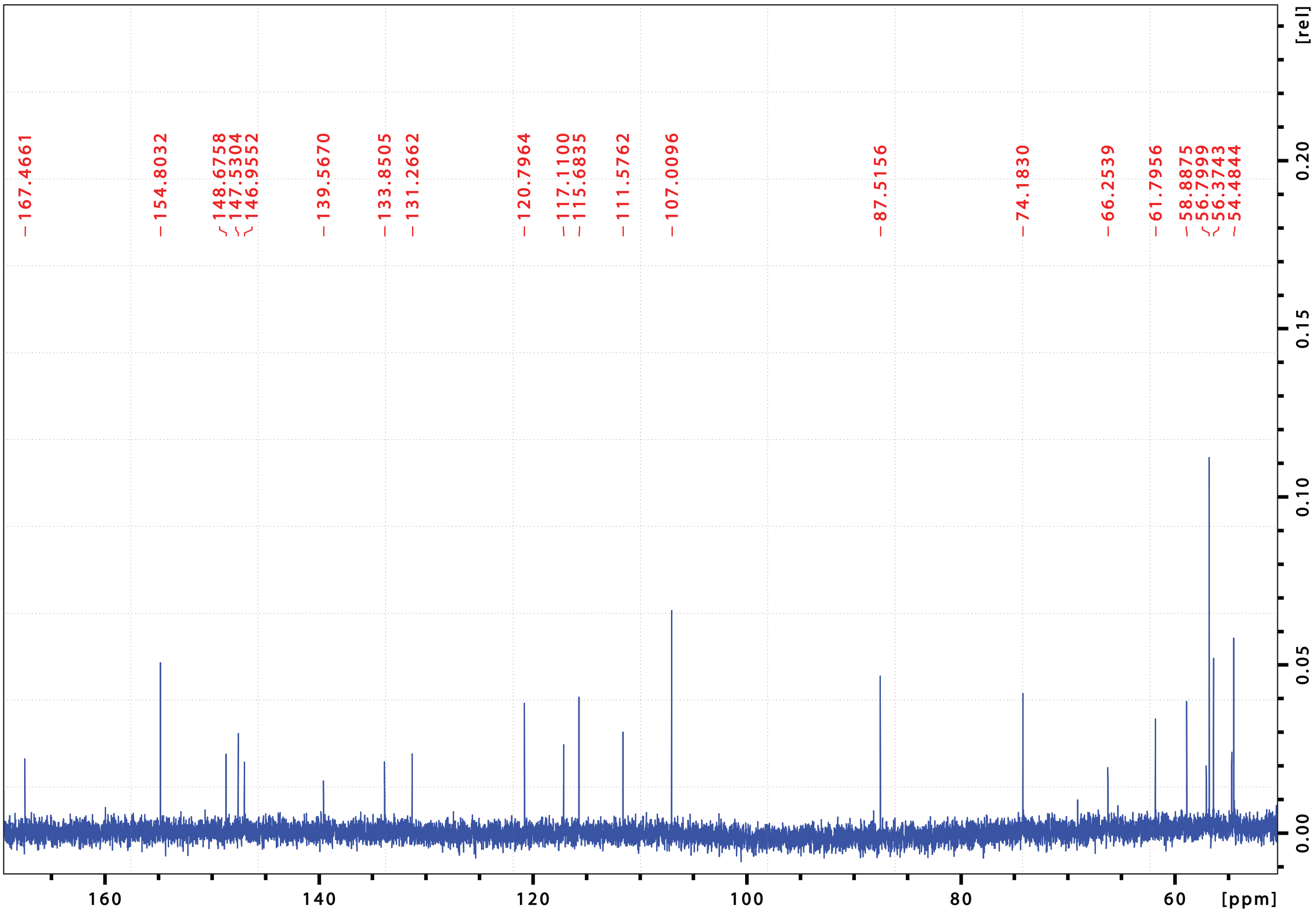

^{1}H (upper) and ^{13}C (lower) NMR spectra of neolignan **1***e* (in methanol-d_4; 800/200 MHz for $^{1}H/^{13}C$)

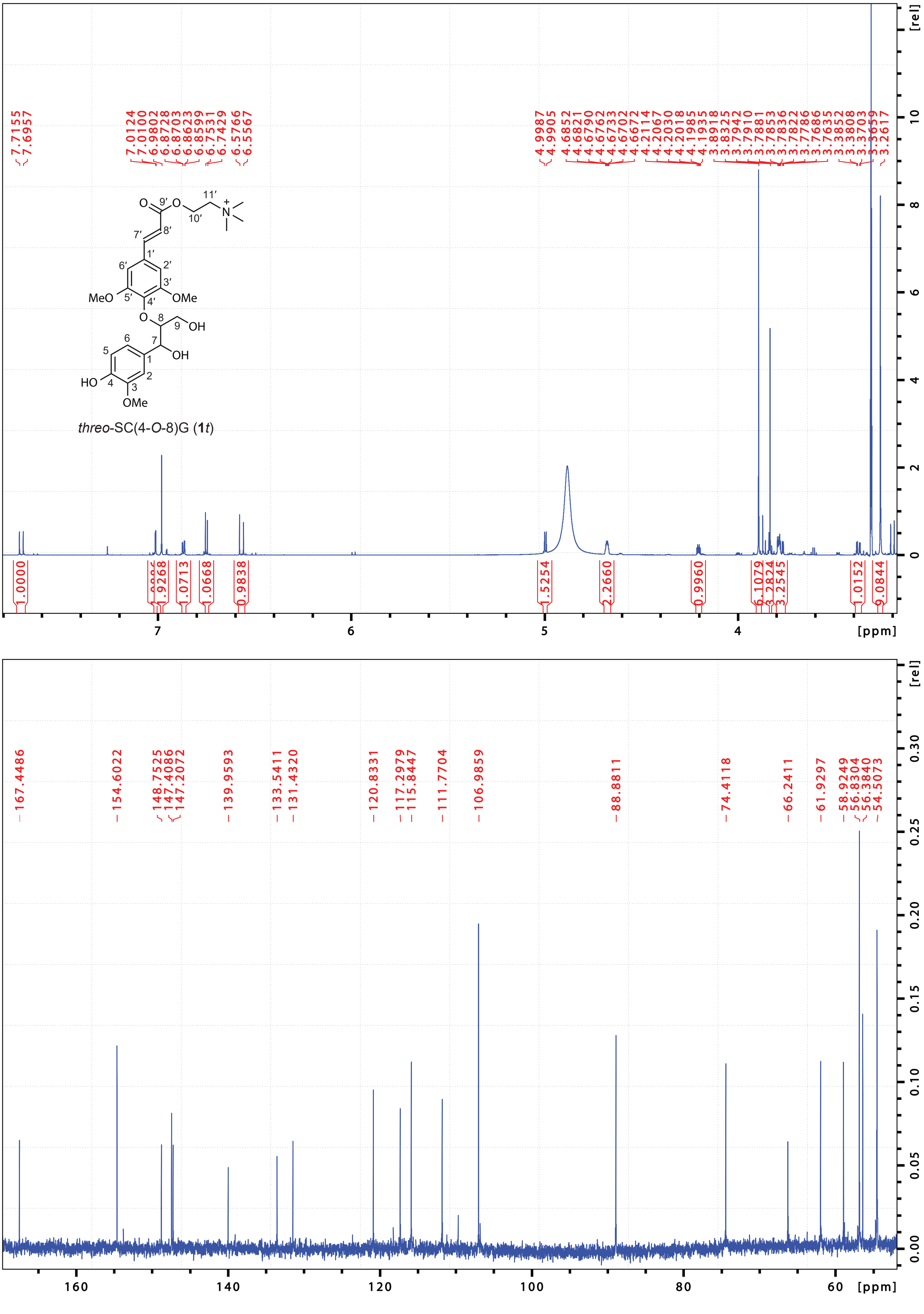

^{1}H (upper) and ^{13}C (lower) NMR spectra of neolignan **1***t* (in methanol-d_4; 800/200 MHz for ^{1}H/^{13}C)

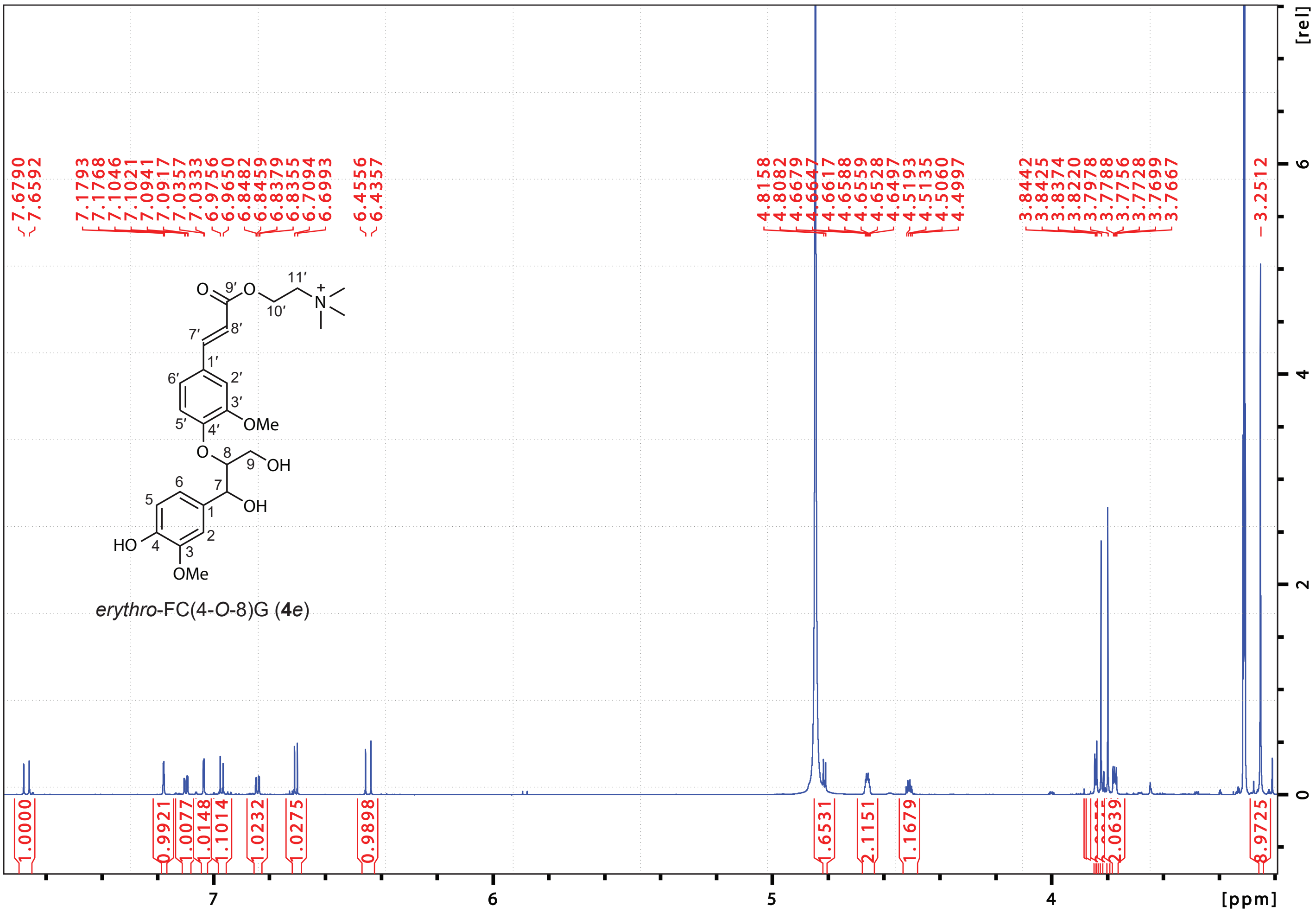

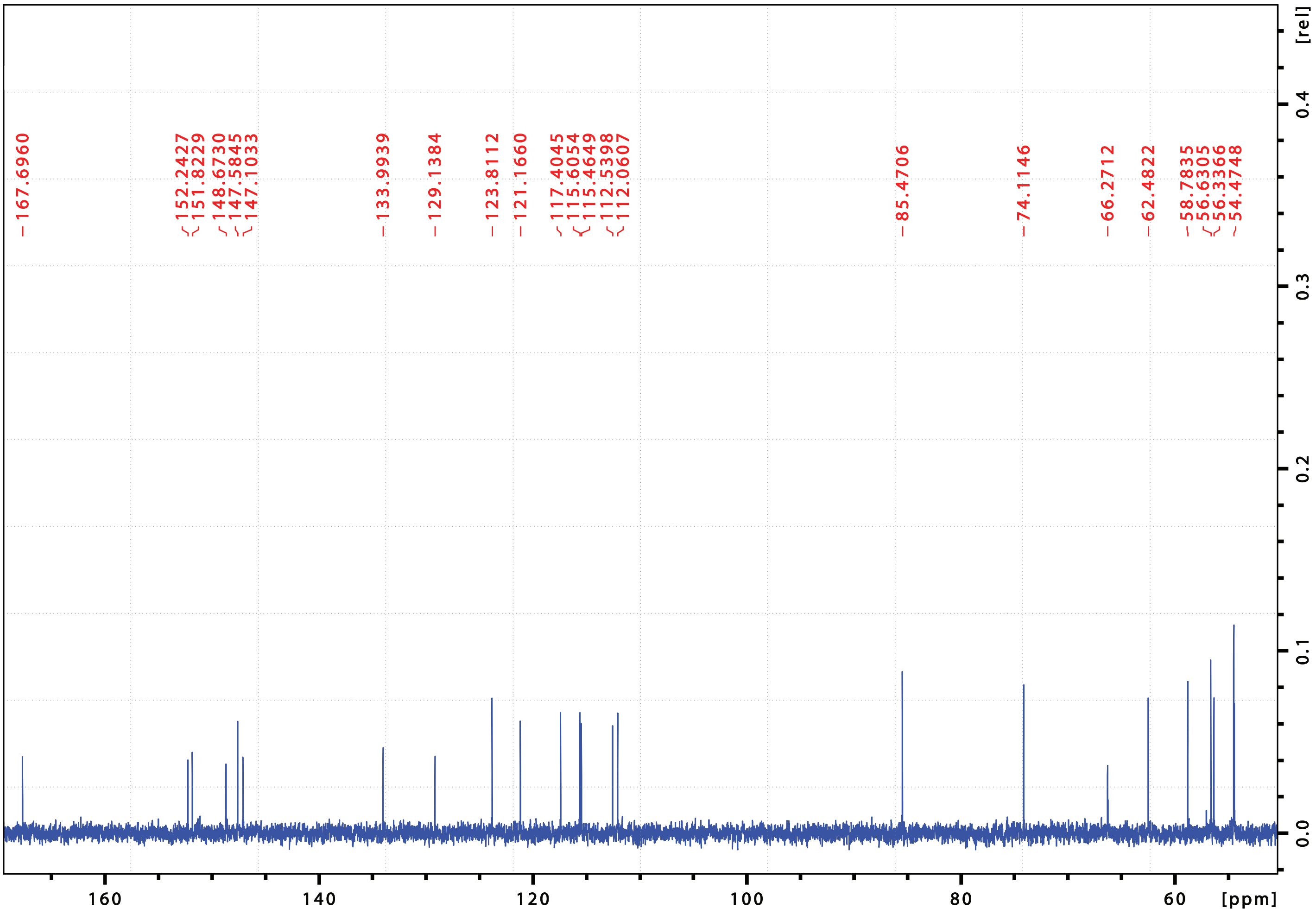

^{1}H (upper) and ^{13}C (lower) NMR spectra of neolignan **4e** (in methanol-d_4; 800/200 MHz for $^{1}H/^{13}C$)

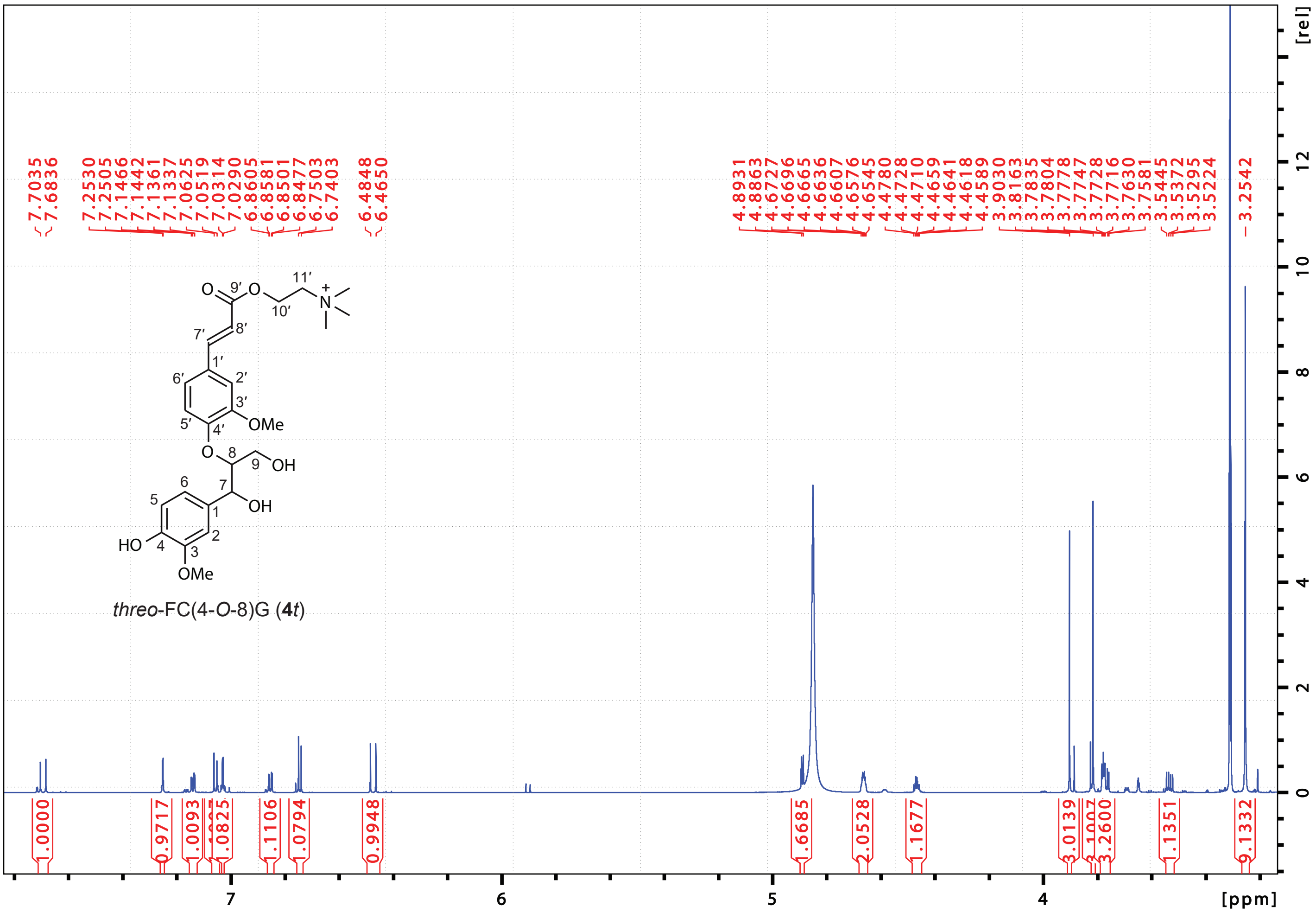

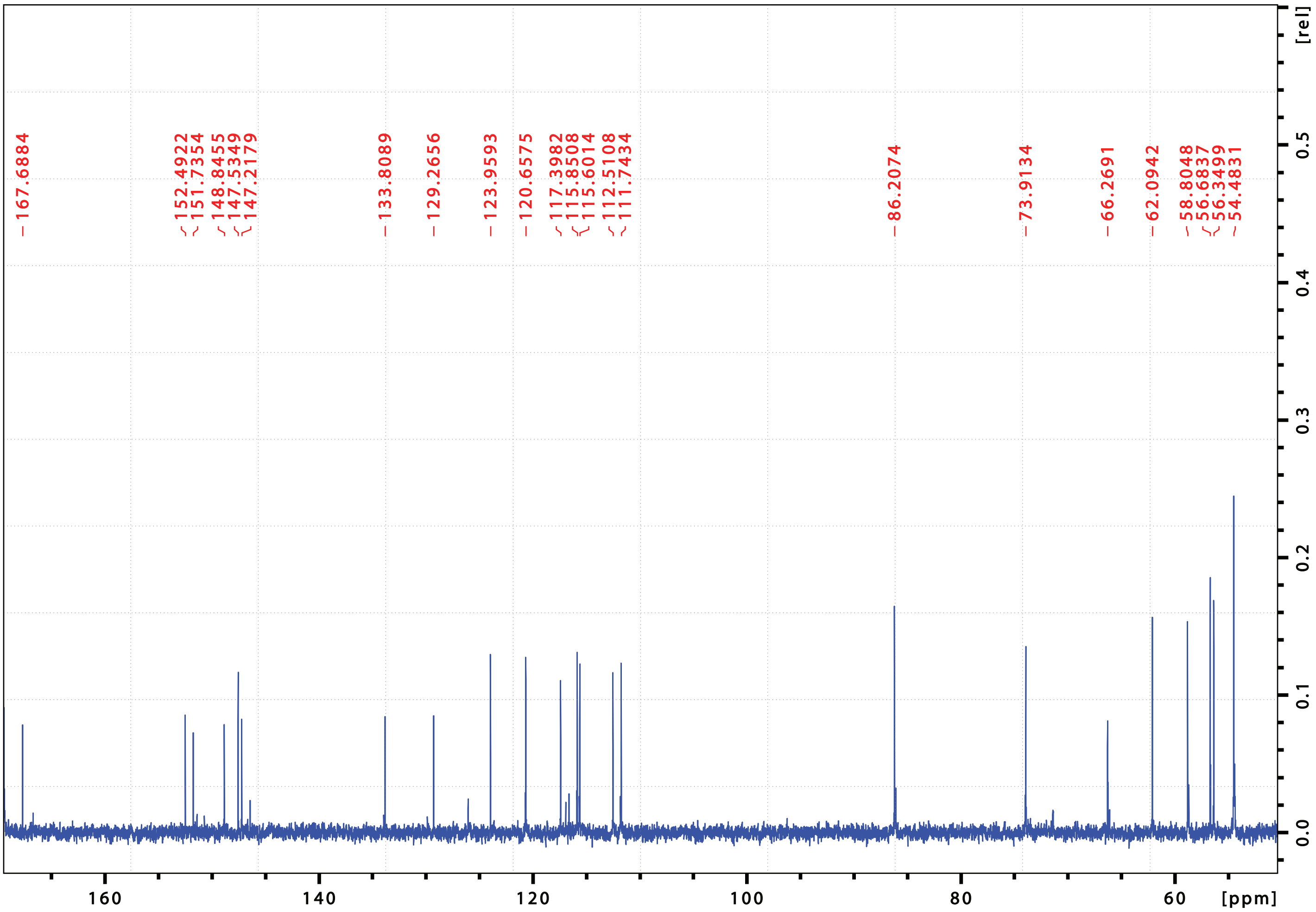

^{1}H (upper) and ^{13}C (lower) NMR spectra of neolignan **4t** (in methanol-d_4; 800/200 MHz for ^{1}H/^{13}C)
