# Supplementary figures and images for: Seed-coat protective neolignans are produced by the dirigent protein AtDP1 and the laccase AtLAC5 in Arabidopsis
Source: Plant Cell. 2020 Nov 27;33(1):129–52. doi: 10.1093/plcell/koaa014 (PMC8136895; doi:10.1093/plcell/koaa014)

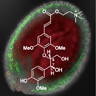

Supplement: koaa014_Supplementary_Data [file koaa014_supplementary_data.zip › tpc.00658.2020-s05.jpg]
